# Oriental freshwater mussels arose in East Gondwana and arrived to Asia on the Indian Plate and Burma Terrane

**DOI:** 10.1038/s41598-022-05257-0

**Published:** 2022-01-27

**Authors:** Ivan N. Bolotov, Rajeev Pasupuleti, Nalluri V. Subba Rao, Suresh Kumar Unnikrishnan, Nyein Chan, Zau Lunn, Than Win, Mikhail Y. Gofarov, Alexander V. Kondakov, Ekaterina S. Konopleva, Artyom A. Lyubas, Alena A. Tomilova, Ilya V. Vikhrev, Markus Pfenninger, Sophie S. Düwel, Barbara Feldmeyer, Hasko F. Nesemann, Karl-Otto Nagel

**Affiliations:** 1 N. Laverov Federal Center for Integrated Arctic Research of the Ural Branch of the Russian Academy of Sciences, Northern Dvina Emb. 23, 163000 Arkhangelsk, Russia; 2 Northern Arctic Federal University, Northern Dvina Emb. 17, 163002 Arkhangelsk, Russia; 3 SSC/IUCN – Mollusc Specialist Group, Species Survival Commission, International Union for Conservation of Nature, Cambridge, CB2 3QZ UK; 4 Institute of Molecular Biotechnology (IMBT), Technical University of Graz, Petersgasse 14, 8010 Graz, Austria; 5 Hyderabad, India; 6 Regional Facility for DNA Fingerprinting (RFDF), Rajiv Gandhi Centre for Biotechnology (RGCB), Trivandrum, 695014 Kerala India; 7 Fauna & Flora International – Myanmar Programme, 34 D/9 San Yae Twin Street, Kaba Aye Pagoda Road, Bahan Township, 11201 Yangon, Myanmar; 8 Biology Department, University of New Brunswick, 100 Tucker Park Road, PO Box 5050, Saint John, NB E2L 4L5 Canada; 9 Department of Zoology, Dawei University, 14043 Dawei, Tanintharyi Region Myanmar; 10 Molecular Ecology Group, Senckenberg Biodiversity and Climate Research Centre (BiK-F), Georg-Voigt-Str. 14-16, 60325 Frankfurt am Main, Germany; 11 Hofheim am Taunus, Germany; 12 Malacological Section, Senckenberg Research Institute and Natural History Museum Frankfurt/M., Senckenberganlage 25, 60325 Frankfurt am Main, Germany

**Keywords:** Biodiversity, Taxonomy, Biogeography

## Abstract

Freshwater mussels cannot spread through oceanic barriers and represent a suitable model to test the continental drift patterns. Here, we reconstruct the diversification of Oriental freshwater mussels (Unionidae) and revise their taxonomy. We show that the Indian Subcontinent harbors a rather taxonomically poor fauna, containing 25 freshwater mussel species from one subfamily (Parreysiinae). This subfamily most likely originated in East Gondwana in the Jurassic and its representatives arrived to Asia on two Gondwanan fragments (Indian Plate and Burma Terrane). We propose that the Burma Terrane was connected with the Indian Plate through the Greater India up to the terminal Cretaceous. Later on, during the entire Paleogene epoch, these blocks have served as isolated evolutionary hotspots for freshwater mussels. The Burma Terrane collided with mainland Asia in the Late Eocene, leading to the origin of the Mekong’s Indochinellini radiation. Our findings indicate that the Burma Terrane had played a major role as a Gondwanan “biotic ferry” alongside with the Indian Plate.

## Introduction

Freshwater mussels (order Unionida) are a diverse and widespread group of large aquatic invertebrates^1,2^, providing a variety of ecosystem services^3,4^. These animals are highly sensitive to human impacts and climate changes^5–9^, revealing dramatically high rates of global decline and regional extinctions^10,11^. Natural dispersal of freshwater mussels mostly occurs at the larval stage together with their fish hosts, and usually requires direct connections between freshwater basins, because they are unable to cross oceanic barriers^2,12–15^. Hence, freshwater mussels are considered to be among the best model organisms for biogeographic and paleogeographic reconstructions^16–21^. Most freshwater mussel species are endemic to a certain faunal region, and multiple single-basin and intra-basin endemics do occur, especially in species-rich faunas such as those of Southeast Asia^22–28^, North America and Mesoamerica^17,29,30^, and tropical Africa^31^. Furthermore, even widespread species share some kind of phylogeographic structure throughout their continuous ranges, e.g. *Anodonta anatina* (Linnaeus, 1758) in Eurasia^32,33^ and *Megalonaias nervosa* (Rafinesque, 1820) in North America^34^.

Recently, freshwater mussels were used as a model group to perform an updated freshwater biogeographic division of South and Southeast Asia^19,23,35^. Based on the Unionidae phylogeny and endemism patterns, this area could be delineated to the Oriental, Sundaland, and East Asian freshwater biogeographic regions^23^. The Oriental Region contains two subregions, i.e. the Indian (Indian Subcontinent from the Indus Basin in Pakistan through Bangladesh, Bhutan, India, Nepal, and Sri Lanka to the coastal basins of the Rakhine State of Myanmar) and Western Indochina (Myanmar from the Irrawaddy [Ayeyarwady] Basin to the Salween [Thanlwin], Tavoy [Dawei], and the Great Tenasserim [Tanintharyi] rivers) subregions^23^. A significant geographic barrier associated with the Indo-Burma Ranges in Western Myanmar and Northeastern India (Naga Hills, Chin Hills, and Rakhine Mountains) separates these entities^23^. The Sundaland Region covers the Mekong, Chao Phraya, and Mae Klong rivers, the drainages of the Thai-Malay Peninsula, and the Greater Sunda Islands^23,28,35,36^. Finally, the massive East Asian Region expands from coastal basins of Vietnam through eastern China, Korea, and Japan to the Russian Far East^20,35,37^.

**Figure 1:**
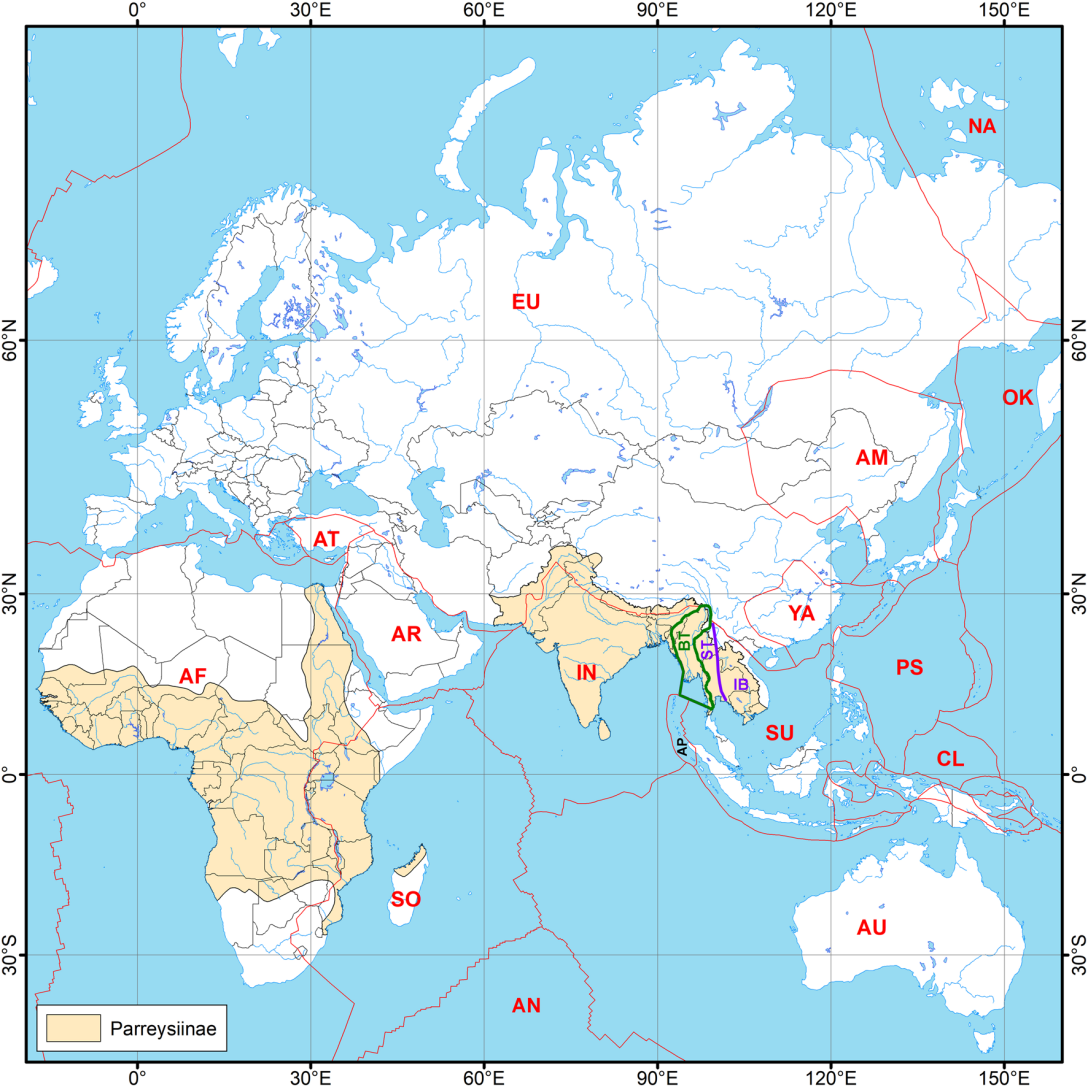
Global distribution of the subfamily Parreysiinae and tectonic plate boundaries. The subfamily range based on available published sources^23,239^ and our own data. The red lines indicate tectonic plate boundaries^240^. The red abbreviations indicate the names of larger tectonic plates: *AF* African; *AM* Amurian; *AN* Antarctic; *AR* Arabian; *AT* Anatolian; *AU* Australian; *CL* Caroline; *IN* Indian; *NA* North American; *OK* Okinawa; *PS* Philippine Sea Plate; *SO* Somalia; *SU* Sunda (with Indochina Block and Sibumasu Terrane); *YA* Yangtze. The boundaries of the Burma Terrane (BT), Sibumasu Terrane (ST), Indochina Block (IB), and the Andaman Platelet (AP) are given based on a series of modern tectonic works^38–40^. The Mogok–Mandalay–Mergui Belt^40^ is placed here within the boundary of the Burma Terrane. The map was created using ESRI ArcGIS 10 software (https://www.esri.com/arcgis). The topographic base of the map was created with Natural Earth Free Vector and Raster Map Data (https://www.naturalearthdata.com) and Global Self-consistent Hierarchical High-resolution Geography (https://www.soest.hawaii.edu/wessel/gshhg). (Map: Mikhail Yu. Gofarov).

At first glance, the freshwater biogeographic division, outlined above, corresponds well to the boundaries of tectonic blocks such as the Indian Plate (Indian Subregion), Burma Terrane or West Burma Block (Western Indochina Subregion), and the Sunda Plate, containing the Indochina Block and Sibumasu Terrane (Sundaland Region)^38–41^ (Fig. 1). Dramatic tectonic movements during the Mesozoic and Cenozoic shaped the modern configuration of these blocks^42,43^. The Indian Plate was a part of East Gondwana and drifted northward as an insular landmass carrying Gondwanan biota^39,44,45^. Furthermore, the body of modern geological, tectonic, paleomagnetic, and paleontological research indicates that the Burma Terrane most likely represents a Gondwanan fragment that rafted to Asia together with the Indian Plate or as a part of a Trans-Tethyan island arc^38,40,41,46–50^. However, it is still unclear whether the continental drift could explain the biogeographic patterns in freshwater mussel distribution throughout the Oriental and Afrotropical regions and whether the disjunctive range of several Unionidae clades could reflect Mesozoic tectonic events^19,31,51,52^. While our knowledge on the taxonomy and evolutionary biogeography of freshwater mussels from tropical Africa, Western Indochina, and Sundaland has largely been improved during the last decade^2,19,22–28,31,35,51,53–56^, the Unionidae fauna of the Indian Subcontinent^57,58^ is still waiting for an integrative taxonomic research and thorough biogeographic modeling.

This study (1) presents a taxonomic review of freshwater mussels from the Indian Subcontinent based on the most comprehensive morphological and DNA sequence datasets sampled to date; (2) reconstructs the origins, macroevolution patterns, and diversification of the Parreysiinae based on a multi-locus time-calibrated phylogeny; and (3) postulates a novel hypothesis on a possible role of the Burma Terrane as a separate “biotic ferry” carrying a derivative of the Gondwanan biota to Asia through continental drift processes. Furthermore, we present a complete reappraisal of Mesozoic freshwater mussel species that were described from the Deccan Intertrappean Beds (Upper Cretaceous) on the Indian Subcontinent and an overview of a few doubtful and uncertain recent taxa that were linked to India.

## Results

### Freshwater mussel fauna of the Indian Subcontinent

**Figure 2:**
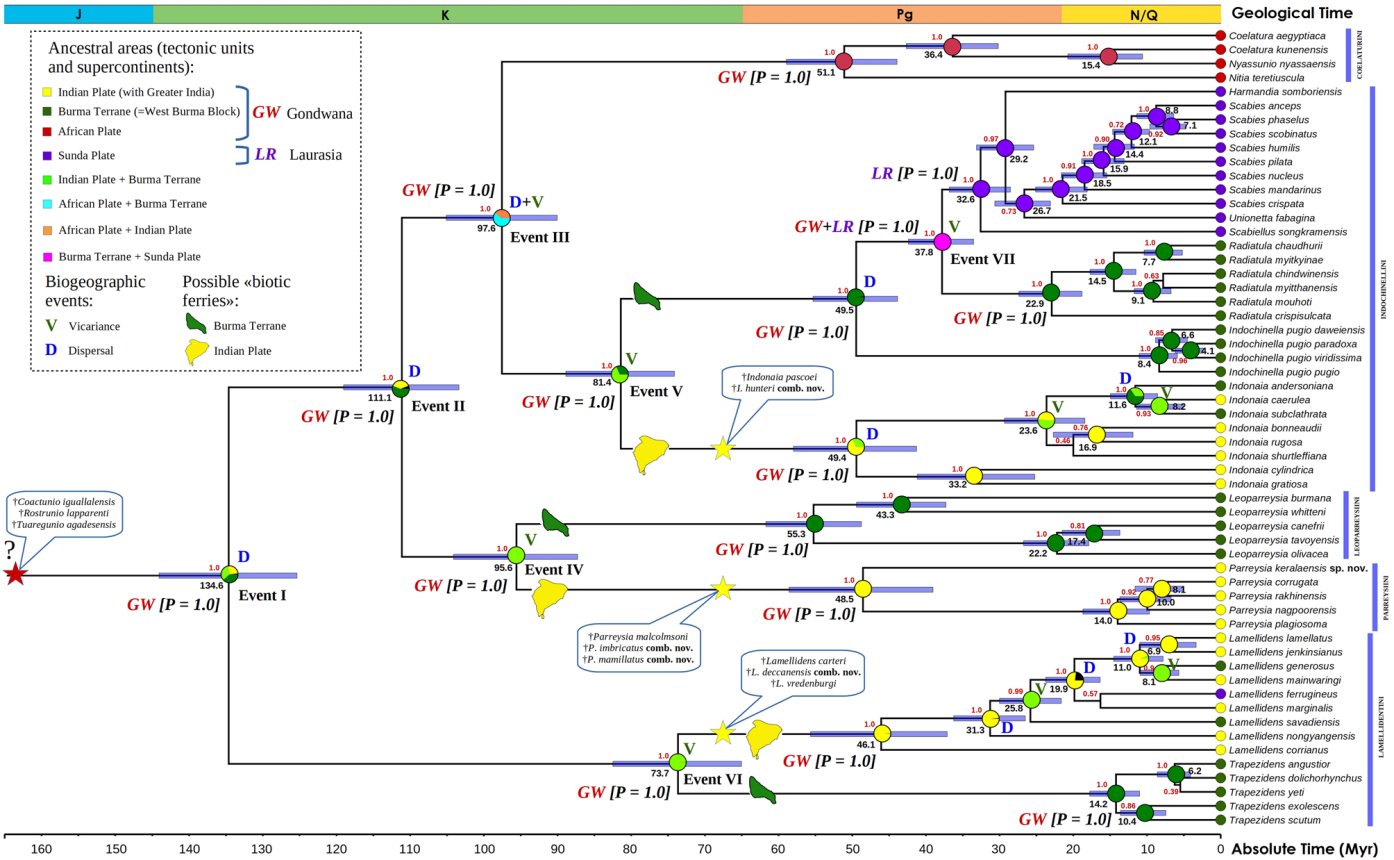
Time-calibrated phylogeny of the Parreysiinae based on the complete data set of mitochondrial and nuclear sequences (five partitions: three codons of *COI* + *16S rRNA* + *28S rRNA*). Events I-VII indicate a series of key biogeographic events, shaping the recent distribution of the subfamily (see “Results”). Nodal circle charts indicate the probabilities of certain ancestral areas based on the combined “tectonic plates” scenario (S-DIVA + DIVALIKE). The Sunda Plate contains the Indochina Block and Sibumasu Terrane^39^. Black color indicates an unexplained origin. Color symbols *GW* (Gondwana) and *LR* (Laurasia) indicate the results of the combined “supercontinents” scenario (S-DIVA + DIVALIKE) with the probabilities (*P*) of each ancestral area being given in square brackets. Stars at branches indicate reliable fossil record of the Mesozoic Parreysiinae in Africa (red) and India (yellow) with available fossil taxa being listed in the corresponding callouts. Taxonomic information on the Mesozoic fossil species from the Indian Subcontinent is given in Table 2. Red numbers near nodes are Bayesian posterior probability (BPP) values of BEAST v. 2.6.3. Black numbers near nodes are the mean node ages. Node bars are 95% HPD of divergence time. Time and biogeographic reconstructions for weakly supported nodes (BPP < 0.70) are not shown. Outgroup taxa are omitted. Stratigraphic chart according to the International Commission on Stratigraphy, 2021 (https://stratigraphy.org/chart).

**Figure 3:**
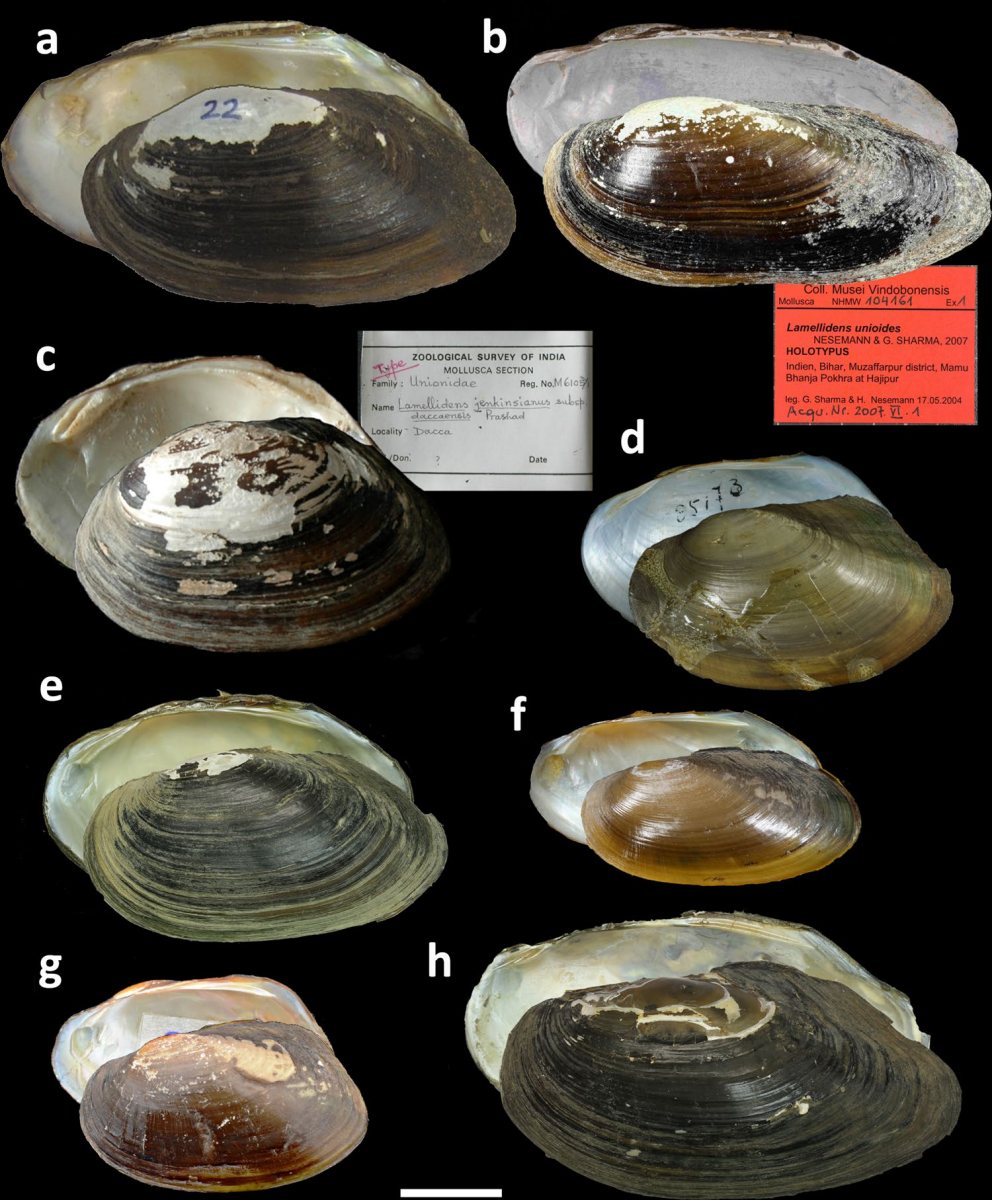
Shell examples of *Lamellidens* species from the Indian Subcontinent. (**a**) *L. corrianus* (Lea, 1834), Gokak, Gatprabha River, Krishna River basin, Western Ghats, Karnataka, India. (**b**) *L. unioides* Nesemann & Sharma in Nesemann et al., 2007, Bihar, Muzaffarpur District, Mamu Bhanja Pokhra at Hajipur, India (holotype NHMW 104161). (**c**) *L. jenkinsianus* (Benson, 1862), Dacca, Bangladesh (= *Parreysia* (s. str.) *daccaensis* Preston, 1912; holotype ZSI M6105/1). (**d**) *L. lamellatus* (Lea, 1838), Ganges River, India (holotype NMNH 85173). (**e**) *L. mainwaringi* Preston, 1912, Kaladan River, Myanmar (specimen RMBH biv153). (**f**) *L. marginalis* (Lamarck, 1819), brook at fish ponds, Hetauda, Ganges Basin, Narayani Zone, Central Region, Nepal (specimen SMF 348831/16.01). **(g)** *L. marginalis* (Lamarck, 1819), Krishna River, Nagarjuna Sagar, Telangana, India (museum lot FBRC ZSI 1227; specimen RLm3). **(h)** *L. nongyangensis* Preston, 1912 **stat. rev.**, Lake of No Return [= Nongyang Lake], Irrawaddy Basin, Myanmar (topotype RMBH biv893/1). Scale bar = 20 mm. Photos: H. Singh, College of Fisheries, Ratnagiri, BOLD Systems BFB021-12, under a CC BY 3.0 license [**a**], N. V. Subba Rao and R. Pasupuleti [**c**, **f**, **g**], NMNH collection database under a CC0 1.0 license [**d**], A. Eschner [**b**], S. Hof [**f**], and E. S. Konopleva [**e**, **h**].

**Figure 4:**
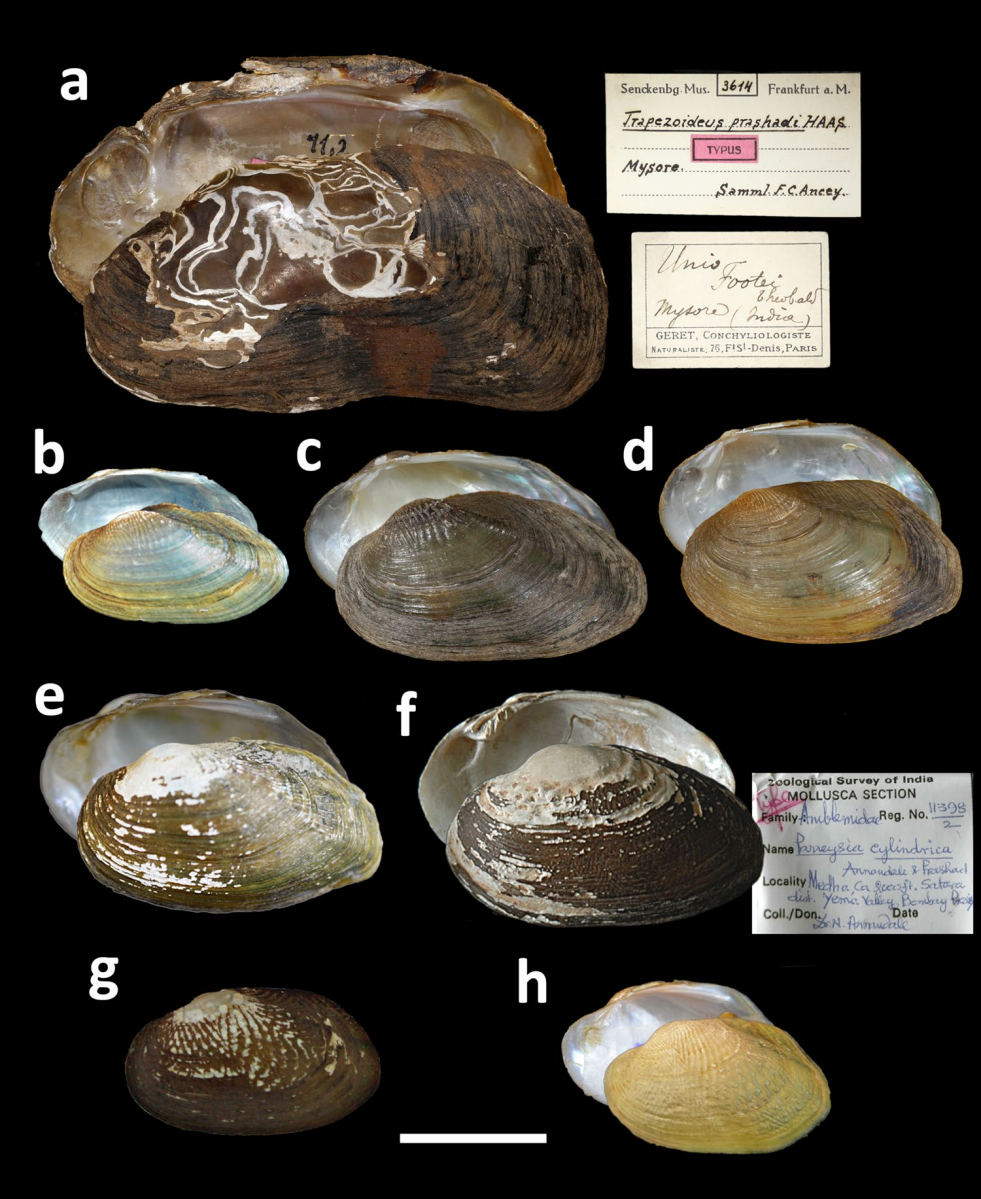
Shell examples of *Arcidopsis* and *Indonaia* species from the Indian Subcontinent. (**a**) *A. footei* (Theobald, 1876), Kistna flumine prope ‘Gutparba falls’ [Gokak Falls, Ghataprabna River, Krishna Basin, India] (= *Trapezoideus prashadi* Haas, 1922; holotype SMF 3614). (**b**) *I. caerulea* (Lea, 1831), fish pond, Krishna River basin, Uppalapadu, Andhra Pradesh, India (museum lot FBRC ZSI 1229; specimen RRc1). **(c)** *I. caerulea* (Lea, 1831), Jhajh nadi, Ganges basin, Narayani Zone, Central Region, Nepal (specimen SMF 348835/17.05). **(d)** *I. gratiosa* (Philippi, 1843) **comb. nov.,** Jhajh nadi, Ganges Basin, Narayani Zone, Central Region, Nepal (specimen SMF 348834/17.15). (**e**) *I. shurtleffiana* (Lea, 1856), Godavari River, Nashik, Maharashtra, India (museum lot FBRC ZSI 1230; specimen RR3). (**f**) *I. cylindrica* (Annandale & Prashad, 1919) **comb. nov.,** Yenna River, Upper Kistna watershed, at Medha, Krishna Basin, Maharashtra, India (syntype ZSI 11398/2). (**g**) *I. cylindrica* (Annandale & Prashad, 1919) **comb. nov.,** Yenna River, Upper Kistna watershed, at Medha, Krishna Basin, Maharashtra, India (syntype ZSI 11398/2). (**h**) *I. rugosa* (Gmelin, 1791) **comb. nov.,** Krishna River, Nagarjuna Sagar, Telangana, India (FBRC ZSI 1222; specimen RRl1). Scale bar = 20 mm. Photos: S. Hof [**a**, **c-d**], and N. V. Subba Rao and R. Pasupuleti [**b**, **e, f**, **g**, **h**].

**Figure 5:**
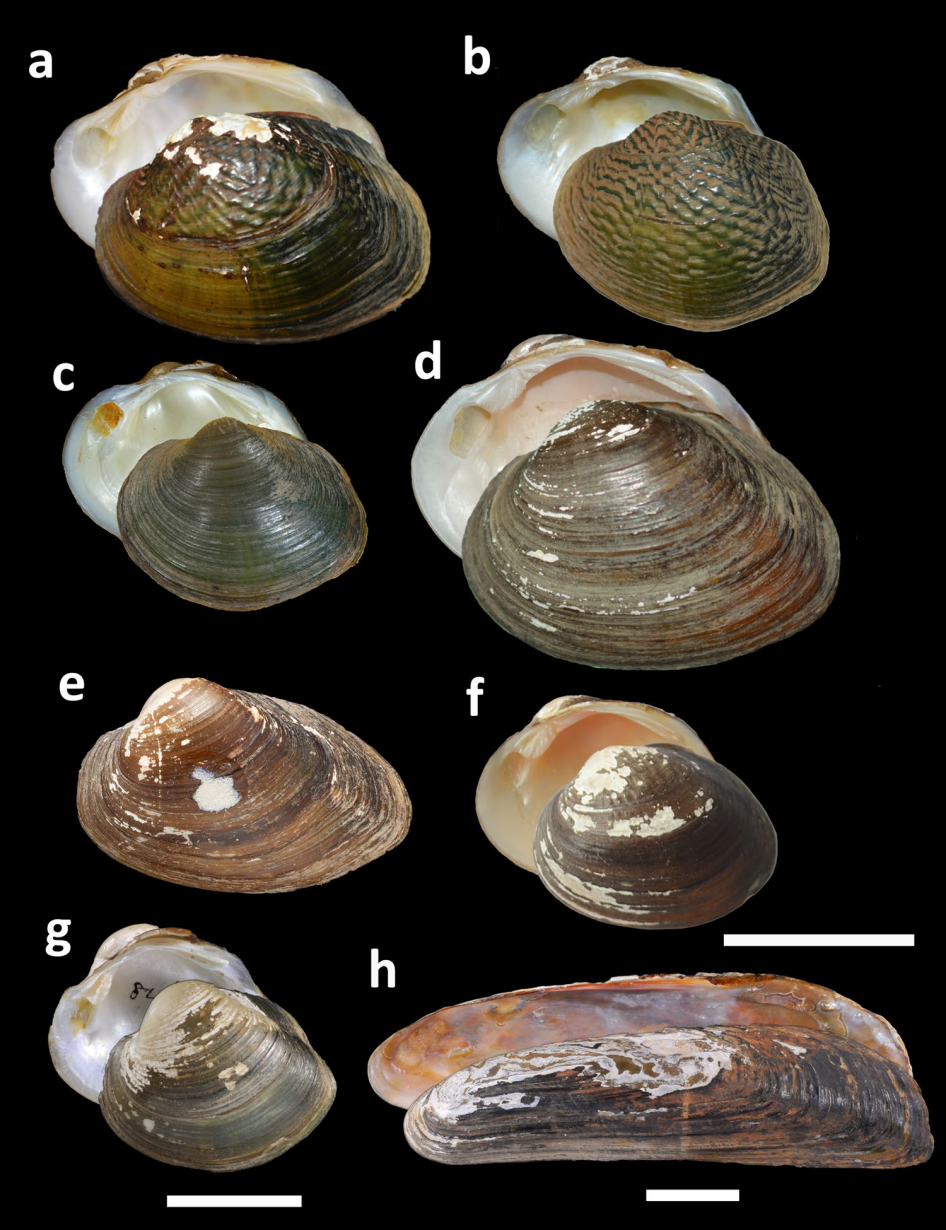
Shells of *Parreysia* and *Balwantia* species from the Indian Subcontinent. **(a)** *P. keralaensis* **sp. nov.,** Periyar River, Aluva, Kerala, India (holotype FBRC ZSI 1007-a/RCB2). (**b**) *P. keralaensis* **sp. nov.,** the type locality (museum lot FBRC ZSI 1007; paratype RCB3). (**c**) *P. corrugata* (Müller, 1774), brook at fish ponds, Hetauda, Ganges Basin, Narayani Zone, Central Region, Nepal (specimen SMF 348829/16.02). (**d**) *P. corrugata* (Müller, 1774). Krishna River, Nagarjuna Sagar, Telangana, India (museum lot FBRC ZSI 1224; specimen RPf1). (**e**) *P. nagpoorensis* (Lea, 1860)*,* Ramganga River near Moradabad, Ganges Basin, Uttar Pradesh, India (= *Unio pinax* Benson, 1862: syntype UMZC I.105035.B^241^). (**f**) *P. nagpoorensis* (Lea, 1860)*,* Krishna River, Nagarjuna Sagar, Telangana, India (museum lot FBRC ZSI 1224; specimen RPf2). **(g)** *P. rajahensis* (Lea, 1841). Rajah’s Tank, India (holotype NMNH 84638). **(h)** *B. soleniformis* (Benson, 1836). Brahmaputra River, India (specimen NMNH 127246). Scale bar = 20 mm [**a**-**e**, **g**]; scale bar = 25 mm [**f**]; scale bar = 30 mm [**h**]. Photos: N. V. Subba Rao and R. Pasupuleti [**a**, **b**, **d**, **f**], S. Hof [**c**], K. Webb [**e**], and NMNH collection database under a CC0 1.0 license [**g**, **h**].

Here, we present the most comprehensive phylogenetic and distribution datasets on freshwater mussels (Unionidae) from the Indian Subcontinent sampled to date with supplement of related taxa from Indochina and Africa (Fig. 2 and Supplementary Fig. 1). The phylogeny was reconstructed using partial sequences of the mitochondrial *cytochrome c oxidase subunit I* (*COI*), *small ribosomal RNA* (*16S rRNA*), and the nuclear *large ribosomal RNA* (*28S rRNA*) genes (Dataset 1). Based on the multi-locus phylogeny, DNA-based species delimitation procedures (Supplementary Fig. 2), and morphological data, we show that the Unionidae fauna of the Indian Subcontinent contains members of one subfamily, the Parreysiinae (Table 1). The total species richness of freshwater mussels on the Indian Subcontinent is rather uncertain due to the lack of DNA sequence data for multiple nominal taxa (Table 1). In summary, we propose a list of 25 valid species, almost all of which seem to be endemic to the subcontinent, though only 17 (68.0%) of those taxa were checked by means of a DNA-based approach. The 25 species recorded from the Indian Subcontinent belong to three tribes: Indochinellini (genus *Indonaia* Prashad, 1918: 8 species), Lamellidentini (genera *Lamellidens* Simpson, 1900 and *Arcidopsis* Simpson, 1900: 9 and 1 species, respectively), and Parreysiini (genus *Parreysia* Conrad, 1853: 6 species) (Figs. 3, 4 and 5)). One more genus, the monotypic *Balwantia* Prashad, 1919 (Fig. 5h), is considered here as Parreysiinae *incertae sedis*. The genera *Arcidopsis*, *Balwantia*, and *Parreysia* are endemic to the Indian Subcontinent, while each of the *Indonaia* and *Lamellidens* also contains three species from Western Indochina. Furthermore, there is one cementing bivalve species, *Pseudomulleria dalyi* (Smith, 1898) (Etheriidae), known to occur in India whose systematic assignment is still unclear (see “Discussion”).

**Table 1:** Taxonomic review of the recent Unionidae (Parreysiinae) from the Indian Subcontinent with supplement of congeneric species from Western Indochina (see Supplementary Note 1 for detail and complete synonymies).

Taxa with new synonyms	Type locality	Distribution	Tectonic block
**Tribe Indochinellini Bolotov, Pfeiffer, Vikhrev & Konopleva, 2018**			
**Genus *Indonaia* Prashad, 1918**			
**The *caerulea*-group**			
**Indonaia andersoniana (Nevill, 1877)	Myadoung, Burma [Irrawaddy River near Mya Taung village, 23.7310° N, 96.1486° E, Myanmar]^210^	Irrawaddy to Salween Basin, Myanmar^211^	Burma Terrane
*Indonaia bonneaudii* (Eydoux, 1838) **comb. rev.** [= *Unio leioma* Benson, 1862 **syn. nov.**]	Les rivières de la presqu'ile de l'Inde [rivers of the Indian Peninsula]^212^	Karli River, Western Ghats, India	Indian Plate
*Indonaia caerulea* (Lea, 1831) [= *Lampsilis argyratus* Rafinesque, 1831 **syn. nov.**; = *Unio nuttallianus* Lea, 1856 **syn. nov.**; = *U. pachysoma* Benson, 1862 **syn. nov.**; = *Trapezoideus dhanushori* Annandale & Prashad, 1921 **syn. nov.**]	Bengal, India^213^	Ganges Basin in India, Nepal, and Bhutan, Brahmaputra and Krishna basins in India; Surma River in Bangladesh; Indus Basin in Pakistan^57,61,63,214^	Indian Plate
*Indonaia rugosa* (Gmelin, 1791) **comb. nov.** [= *Diplasma striata* Rafinesque, 1831 **syn. nov.**; = *Unio scobina* Hanley, 1856 **syn. nov.**; = *Nodularia* (*Radiatula*) *lima* Simpson, 1900 **syn. nov.**]	Coromandel fluviis [rivers of the Coromandel Coast of India]^215^	Ganges, Brahmaputra, and Krishna basins, India	Indian Plate
*Indonaia shurtleffiana* (Lea, 1856) [= *I. khadakvaslaensis* Ray, 1966 **syn. nov.**]	Sina River, < … > Ahmednugger, India [upper reaches of the Sina River near Ahmednager, approx. 19.0835° N, 74.7281° E, Krishna Basin, Maharashtra, India]^216^	Krishna and Godavari basins, India	Indian Plate
**Indonaia subclathrata (Martens, 1899)	Chindwinfluss bei Kalewa und bei Matu < … > ; einige Stücke auch im Irawaddi selbst bei Yenangyoung [Chindwin River near Kalewa and Matu, approx. 23.1991° N, 94.3071° E, several specimens also from Irrawaddy River near Yenangyaung, approx. 20.4347° N, 94.8720° E, Myanmar]^217^	Lower Manipur River and a corresponding section of the Chindwin River, Myanmar^53,211^	Burma Terrane
*, **Indonaia theobaldi (Preston, 1912)	Manipur, Assam^218^	Upper Manipur Valley (including Logtak Lake), India^219^	Burma Terrane
**The *cylindrica*-group**			
*Indonaia cylindrica* (Annandale & Prashad, 1919) **comb. nov.**	Yenna River, Upper Kistna watershed, at Medha [Venna River at Medha (now Kanher Reservoir), 17.7887° N, 73.8254° E, Krishna Basin, Maharashtra, India]^220^	Endemic to the Upper Krishna Basin, India^220^	Indian Plate
*Indonaia gratiosa* (Philippi, 1843) **comb. nov.** [= *Unio corbis* Hanley, 1856 **syn. nov.**; = *U. occatus* Lea, 1860 **syn. nov.**; = *U. siliguriensis* Preston, 1908 **syn. nov.**]	Nova Hollandia [erroneous; it was collected somewhere in India]^221^	Ganges and Brahmaputra basins, India and Nepal	Indian Plate
**The *involuta*-group**			
*Indonaia involuta (Hanley, 1856)	Assam^222^	Upper Brahmaputra Basin in India and Surma River in Bangladesh	Indian Plate
*Indonaia olivaria (Lea, 1831)	Bengal^213^	Ganges Basin, India^61^	Indian Plate
**Tribe Lamellidentini Modell, 1942**			
**Genus *Arcidopsis* Simpson, 1900**			
*Arcidopsis footei (Theobald, 1876)	Kistna flumine prope ‘Gutparba Falls’ [Gokak Falls, Ghataprabha River, 16.1929° N, 74.7827° E, Krishna Basin, southwestern India]^223^	Upper part of the Krishna Basin in Western Ghats, India^71,72,124^	Indian Plate
**Genus *Lamellidens* Simpson, 1900** [= *Velunio* Haas, 1919 **syn. nov.**]			
**The *corrianus*-group**			
*Lamellidens corrianus* (Lea, 1834) [= *Unio theca* Benson, 1862 **syn. nov.**]	Calcutta, India^224^	Ganges and Krishna basins, India	Indian Plate
*Lamellidens nongyangensis* Preston, 1912 **stat. rev.** [= *L. narainporensis* Preston, 1912 **syn. nov.**; our first reviser action on the precedence of simultaneous synonyms]	Nongyang Lake, South of Patkai [Lake of No Return, 27.2192° N, 96.1439° E, Irrawaddy Basin, Myanmar]^218^	Ganges Basin in India, with an isolated population in Lake of No Return, Irrawaddy Basin, Myanmar	Indian Plate, with one isolated population on Burma Terrane
**Lamellidens savadiensis (Nevill, 1877)	At Sawady in the Thengleng Stream [Sawadi village, 24.1510° N, 97.1502° E, Myanmar], also at Bhamo [Irrawaddy River near Bhamo city, 24.2594° N, 97.2202° E, Myanmar] and at Shuaygoomyo [Irrawaddy River near Shwegu town, 24.2291° N, 96.7910° E, Myanmar]; four young specimens found at Myadoung [Irrawaddy River near Mya Taung village, 23.7310° N, 96.1486° E, Myanmar] probably also belong to this form^210^	Middle Irrawaddy (including Lake Indawgyi) and Sittaung basins, Myanmar^56^	Burma Terrane
*Lamellidens unioides Nesemann & Sharma in Nesemann et al., 2007	Mamu Bhanja Pokhra at Hajipur, Muzaffarpur District, Bihar, India [pond, 25.6758° N, 85.2250° E, Mamu Bhanja, Hajipur, Ganges Basin, Muzaffarpur District, Bihar, India]^61^	Ganges Basin in India^61^	Indian Plate
**The *marginalis*-group**			
*Lamellidens candaharicus (Hutton, 1849) [= *L. rhadinaeus* Annandale & Prashad, 1919 **syn. nov.**]	Canals at Candahar [Kandahar, 31.6148° N, 65.7198° E, Sistan/Helmand Basin, Afghanistan]^225^	Endorheic Sistan/Helmand Basin, eastern Iran and Afghanistan^78,225^	Indian Plate
**Lamellidens ferrugineus (Annandale, 1918) **stat. rev.** [= *Physunio micropteroides* Annandale, 1918 **syn. nov.**; our first reviser action on the precedence of simultaneous synonyms]	The semi-liquid mud at the bottom of the central region of the Inle Lake in water from 7 to 12 feet deep [central part of Lake Inle, 20.5903° N, 96.9025° E, Salween Basin, Myanmar]^226^	Lake Inle and streams around, Salween Basin, Myanmar	Connection of Burma Terrane with Sunda Plate
*Lamellidens friersoni (Simpson, 1914) **comb. nov.**	Assam, India^74,79^	Upper Brahmaputra Basin, Assam, India^74,79^	Indian Plate
**Lamellidens generosus (Gould, 1847) [= *Unio consobrinus* Lea, 1860 **syn. nov.**; = *L. brandti* Bolotov, Konopleva & Vikhrev, 2017 **syn. nov.**]	Newville, Tavoy, British Burmah [Hlaingbwe River near the former Newville village, 16.9834° N, 97.9043° E, Myanmar]^227^	Irrawaddy to Lower Salween Basin (including Haungthayaw, Hlaingbwe, and Ataran) in Myanmar; southwestern Yunnan in China	Burma Terrane but reaches the western margin of the Sunda Plate
*Lamellidens jenkinsianus* (Benson, 1862)	Fluvio Assamensi Berhampooter dicto [Brahmaputra River, Assam, India]^228^	Ganges, Meghna, Brahmaputra, Godavari, Krishna, and Ambā basins, India and Bangladesh; a few occurrences from Bhutan^63^	Indian Plate
*Lamellidens lamellatus* (Lea, 1838)	Ganges River, India^229^	Ganges, Krishna, and Mahanadi basins, India	Indian Plate
*Lamellidens mainwaringi* Preston, 1912 [= *L. phenchooganjensis* Preston, 1912 **syn. nov.**; our first reviser action on the precedence of simultaneous synonyms]	Siliguri [Siliguri, Ganges Basin, West Bengal, India]^218^	Ganges, Karli, Kalni, and Kaladan rivers, India, Bangladesh, and western Myanmar	Indian Plate
*Lamellidens marginalis* (Lamarck, 1819)	Bengale, dans les rizières [rice fields, Bengal, India]^230^	Ganges and Krishna basins, India and Nepal; Sri Lanka; Indus Basin in Pakistan^57,61^	Indian Plate
**Tribe Parreysiini Henderson, 1935**			
**Genus *Parreysia* Conrad, 1853**			
**The *keralaensis*-group**			
*Parreysia keralaensis* Bolotov, Pasupuleti & Subba Rao **sp. nov.**	Periyar River, 10.11° N, 76.37° E, Aluva, Kerala, India	Endemic to Periyar and Pampa basins, India	Indian Plate
**The *corrugata*-group**			
*Parreysia corrugata* (Müller, 1774) [= *Unio sikkimensis* Lea, 1859 **syn. nov.**; = *U. favidens* Benson, 1862 **syn. nov.**; = *U. favidens* var. *chrysis* Benson, 1862 **syn. nov.**; = *U. favidens* var. *deltae* Benson, 1862 **syn. nov.**; = *U. favidens* var. *densa* Benson, 1862 **syn. nov.**; = *U. favidens* var. *trigona* Benson, 1862 **syn. nov.**; = *U. favidens* var. *marcens* Benson, 1862 **syn. nov.**; = *U. favidens* var. *viridula* Benson, 1862 **syn. nov.**; = *U. laevirostris* Benson, 1862 **syn. nov.**; = *U. smaragdites* Benson, 1862 **syn. nov.**; = *U. tripartitus* Lea, 1863 **syn. nov.**; = *U. gowhattensis* Theobald, 1873 **syn. nov.**; = *U. feddeni* Theobald, 1873 **syn. nov.**; = *Parreysia* (*P.*) *annandalei* Preston, 1912 **syn. nov.**; = *P. favidens* var. *assamensis* Preston, 1912 **syn. nov.**]	In fluviis littoris Coromandel [rivers of the Coromandel Coast of India]^231^	Ganges Basin in India and Nepal; Brahmaputra, Krishna, and Godavari basins in India; Surma River in Bangladesh; Sri Lanka; Indus Basin in Pakistan	Indian Plate
*Parreysia plagiosoma* (Benson, 1862) **stat. rev.** [= *Unio tennentii* Hanley & Theobald, 1872 **syn. nov.**]	Bengal^228^	Ganges and Vaghotan basins, India	Indian Plate
*Parreysia rakhinensis* Bolotov et al., 2020	Kyeintali Stream upstream of Ohtein village, 17.9193° N, 94.5946° E, Rakhine State, Myanmar^23^	Rakhine Coast, western Myanmar^23^	Indian Plate
*Parreysia nagpoorensis* (Lea, 1860) **stat. rev.** [= *Unio merodabensis* Küster, 1861 **syn. nov.**; = *U. triembolus* Benson, 1862 **syn. nov.**; = *U. trirostris* Musgrave, 1863 **syn. nov.**; *= U. pinax* Benson, 1862 **syn. nov.**]	Ambijiri Tanks, Nagpoor, Bengal, India [Ambazari Pond in Nagpur, 21.1278° N, 79.0439° E, Godavari Basin, Maharashtra, India]^232^	Ganges, Krishna, Godavari, and Karli (Pithdhaval) basins, India	Indian Plate
**The *rajahensis*-group**			
*Parreysia rajahensis (Lea, 1841)	Rajah’s Tank, Calcutta, India [most likely inaccurate; the type shell was probably collected somewhere in the Narmada River basin, e.g. from Maharaja’s Tank in Jabalpur]^97,233^	Upstream section of the Narmada River, India^97^	Indian Plate
**Parreysiinae *incertae sedis***			
**Genus *Balwantia* Prashad, 1919**			
*Balwantia soleniformis (Benson, 1836)	The hills on the N.E. Frontier of Bengal (Silhet) [Sylhet Division, Upper Meghna Basin, northeastern Bangladesh]^234^	Upper Brahmaputra, Upper Barak (Dhaleswari), and Upper Meghna basins, India and Bangladesh^89–91^	Indian Plate

*Species whose DNA sequences are not available. All of the other species were studied by means of a molecular approach. **Taxa endemic to the Western Indochina Subregion (Burma Terrane) that are lacking in the fauna of Indian Subcontinent.

### Macroevolution and evolutionary biogeography of the Parreysiinae

Our combined supercontinent-based biogeographic modeling (S-DIVA + DIVALIKE) reveals that this subfamily most likely originated and diversified on Gondwana and its fragments (probability = 1.00), with a secondary radiation of the so-called Mekong’s Indochinellini (sensu Pfeiffer et al., 2018)^51^, a compact but diverse monophyletic subclade, in the Sundaland Subregion (probability = 1.00) (Fig. 2). The earliest split within the subfamily was associated with the separation of the Lamellidentini from other taxa by a dispersal event (probability = 1.00) and did occur in the Early Cretaceous (mean age = 135 Myr, 95% HPD = 125–144 Myr) (Fig. 2: Event I). Our combined tectonic plate-based ancestral area reconstruction (S-DIVA + DIVALIKE) suggests that this split occurred on the Burma Terrane, Indian Plate or on both of these blocks with equal probability (Fig. 2). The Parreysiini + Leoparreysiini clade most likely separated from the Coelaturini + Indochinellini clade by a dispersal event (probability = 1.00) in the mid-Cretaceous (mean age = 111 Myr, 95% HPD = 103–119 Myr) (Fig. 2: Event II). The Burma Terrane and Indian Plate are returned as the most probable ancestral areas during this event by our combined model (Fig. 2).

A series of vicariance events occurred in the Late Cretaceous: Coelaturini vs. Indochinellini (mean age = 98 Myr, 95% HPD = 90–105 Myr) (Fig. 2: Event III); Parreysiini vs. Leoparreysiini (mean age = 96 Myr, 95% HPD = 87–104 Myr) (Fig. 2: Event IV); *Indonaia* vs. the rest of the Indochinellini (mean age = 81 Myr, 95% HPD = 74–89 Myr) (Fig. 2: Event V); and *Lamellidens* vs. *Trapezidens* (mean age = 74 Myr, 95% HPD = 65–83 Myr) (Fig. 2: Event VI). The first vicariance event was preceded by a dispersal event and most likely corresponded to a split between Africa and Burma Terrane (probability = 0.60) or between Africa and Indian Plate (probability = 0.40). The other events in this series could be linked to repeated splits and reconnections between the Indian Plate and Burma Terrane (probability = 1.00).

The Mekong’s Indochinellini did separate from the *Radiatula* clade near the terminal Eocene (mean age = 38 Myr, 95% HPD = 34–42 Myr) (Fig. 2: Event VII). Our ancestral area reconstruction suggests that this vicariance event could be linked to a direct connection between Burma Terrane and mainland Asia (probability = 1.00). Exchanges between freshwater mussel faunas of the Indian Plate and Burma Terrane, which traced well in *Indonaia* and *Lamellidens* radiations, started in the Late Oligocene (mean age = 24–26 Myr, 95% HPD = 18–30 Myr) and continued in the Late Miocene (mean age = 8 Myr, 95% HPD = 6–11 Myr) (Fig. 2). These vicariance events reflect direct connections (river captures) between freshwater systems of these terranes based on our combined biogeographic reconstruction (probability = 1.00 in almost all cases).

## Taxonomy of freshwater mussels from the Indian Subcontinent

This taxonomic section is largely based on our novel phylogenetic and morphological research (Fig. 2 and Supplementary Note 1). A brief overview of the fauna is presented in Table 1, while the taxonomic account and explanatory comments for each species are given in the Supplementary Note 1. Additionally, one new species of the genus *Parreysia* from Southwestern India is described herein.

Family Unionidae Rafinesque, 1820.

Subfamily Parreysiinae Henderson, 1935.

Tribe Indochinellini Bolotov, Pfeiffer, Vikhrev & Konopleva, 2018.

Genus *Indonaia* Prashad, 1918.

Type species: *Unio caeruleus* Lea, 1831 (by original designation)^59^.

Distribution: Indian and Western Indochina subregions: from Indus River in Pakistan^60^ through India^57,61^, Bangladesh, Nepal^61,62^, and Bhutan^63^ to the Salween River in Myanmar^56^.

Comments: This genus contains not less than 11 recent species, eight of which occur on the Indian Subcontinent and three in the Western Indochina (Table 1 and Fig. 4b–h). Here, we tentatively delineate these taxa to three informal species groups (Fig. 2 and Table 1). The *caerulea*-group contains eight *Radiatula*-like species having an ovate or elongated shell of moderate thickness. The *cylindrica*-group joins two *Parreysia*-like species with a thicker, ovate shell. Finally, the *involuta*-group combines two peculiar species, sharing a thin, fragile shell. The pseudocardinal teeth in the latter group are lamellar, quite similar to those in *Lamellidens* taxa. The *caerulea*- and *cylindrica*-groups are largely supported by our phylogeny. The *involuta*-group was separated by means of a morphological approach alone, because the DNA sequences of both *Indonaia involuta* (Hanley, 1856) and *I. olivaria* (Lea, 1831) are not available. Based on the phylogenetic data, we transfer the nominal species *Parreysia cylindrica* Annandale & Prashad, 1919 to the genus *Indonaia* and propose *I. cylindrica* **comb. nov.** (Fig. 2, Fig. 4f–g, and Supplementary Figs. 1–2). Additionally, we revise the synonymy for nominal taxa in this genus (Table 1 and Supplementary Note 1). We chose not to discuss the nominal taxon *Indonaia substriata* (Lea, 1856) [= *Nodularia* (s. str.) *pecten* Preston, 1912]^2^ from Thailand here, because its generic placement and range are unclear and require future research efforts.

Two Late Cretaceous fossil species from the Intertrappean Beds of the Deccan Plateau in India are considered here as the earliest members of the *Indonaia* crown group, i.e. †*I. hunteri* (Hislop, 1860) **comb. nov.** and †*I. pascoei* Prashad, 1928 (Table 2 and Supplementary Note 2). Several younger fossil species in this genus were described from Miocene to Pliocene deposits (mostly the Siwalik Group^64^) in India, Pakistan, Nepal, and Myanmar^65–69^.

**Table 2:** Taxonomic review of the fossil Unionidae (Parreysiinae) from the Maastrichtian Intertrappean Beds of the Deccan Plateau, Indian Plate (see Supplementary Note 2 for detail).

Taxon	Original combination and synonyms	Type locality	Type specimen**
**Tribe Indochinellini Bolotov, Pfeiffer, Vikhrev & Konopleva, 2018**			
**Genus *Indonaia* Prashad, 1918**			
†*Indonaia hunteri* (Hislop, 1860) **comb. nov.**	†*Unio hunteri* Hislop (1860)^235^: p. 174, pl. 6, Fig. 25; †*Parreysia hunteri* (Hislop, 1860): Modell (1969)^67^: p. 11	Karuni, 100 miles S.S.W. of Nagpur city, Hyderabad Territory, British India [near Karanji Village, 19.8567° N, 78.3141° E, Deccan Plateau, Telangana, India]^235^	Lectotype PIMB 948 [designated by Hartman et al., 2008]^145^
†*Indonaia pascoei* Prashad, 1928	†*Indonaia pascoei* Prashad (1928)^66^: p. 311, pl. 25, Figs. 4–5; †*Palindonaia pascoei* (Prashad, 1928): Modell (1969)^67^: p. 9	“…at a point situated 2 furlongs S. 10° W. of Nawapet (17°43′30" 78°23′45"), Hyderabad State (Deccan)” [ca. 400 m SSW of Nawabpet Village, 17.7177° N, 78.3933° E, Telangana, India]^66^	Holotype [based on original designation; not traced but it is probably in the collection of Geological Survey of India]^66^
**Tribe Lamellidentini Modell, 1942**			
**Genus *Lamellidens* Simpson, 1900**			
†*Lamellidens carteri* (Hislop, 1860)	†*Unio carteri* Hislop (1860)^235^: p. 175, pl. 7, Fig. 28; †*Lamellidens carteri* (Hislop, 1860): Modell (1969)^67^: p. 11	Karuni, 100 miles S.S.W. of Nagpur city, Hyderabad Territory, British India [near Karanji Village, 19.8567° N, 78.3141° E, Deccan Plateau, Telangana, India]^235^	Lectotype PIMB 949 [designated by Hartman et al., 2008]^145^
†*Lamellidens deccanensis* (J. Sowerby in Malcolmson, 1840) **comb. nov.**	†*Unio deccanensis* J. Sowerby in Malcolmson (1840)^236^: pl. 47, Figs. 4–10; †*Hyriopsis deccanensis* (J. Sowerby, 1827) [erroneous publication year]: Modell (1969)^67^: p. 12	Munnoor^236^ [near Muthnur Village, 19.5192° N, 78.4657° E, Nirmal Hills, Telangana, India]^237^	Lectotype PIMB 947 (of rather poor quality) [designated by Hartman et al., 2008]^145^
†*Lamellidens vredenburgi* Prashad, 1921	†*Lamellidens vredenburgi* Prashad (1921)^238^: p. 368, pl. 12, Figs. 1–2	Goraha, Narbada [probably Gora Village, 1.8608° N, 73.6830° E, Narmada District, Gujarat, India]^238^	Holotype [based on original designation; not traced but it is probably in the collection of Geological Survey of India]^238^
**Tribe Parreysiini Henderson, 1935**			
**Genus *Parreysia* Conrad, 1853**			
†*Parreysia imbricatus* (Hislop, 1860) **comb. nov.**	†*Unio imbricatus* Hislop (1860)^235^: p. 175, pl. 7, Fig. 27a-c; †*Schistodesmus imbricatus* (Hislop, 1860): Modell (1969)^67^: p. 10	Mekalgandi Ghat, 150 miles S.S.W. of Nagpur city, Hyderabad Territory, British India^235,236^ [near Muthnur Village, 19.5192° N, 78.4657° E, Nirmal Hills, Telangana, India]^237^	Lectotype PIMB 950 [designated by Hartman et al., 2008]^145^
†*Parreysia malcolmsoni* (Hislop, 1860)	†*Unio tumida* J. Sowerby in Malcolmson (1840): pl. 47, Figs. 11–12 [unavailable as a primary homonym]^236^; †*U. malcolmsoni* Hislop (1860): p. 174 [new name for †*U. tumida* Sowerby in Malcolmson, 1840]^235^; †*Parreysia malcolmensis* Modell (1969): p. 11 [error for †*U. malcolmsoni* Hislop, 1860]^67^	Mekalgandi Ghat, 150 miles S.S.W. of Nagpur city, Hyderabad Territory, British India^235,236^ [near Muthnur Village, 19.5192° N, 78.4657° E, Nirmal Hills, Telangana, India]^237^	Lectotype PIMB 953 (complete shell) [designated by Hartman et al., 2008]^145^
†*Parreysia mamillatus* (Hislop, 1860) **comb. nov.**	†*Unio mamillatus* Hislop (1860)^235^: p. 175, pl. 7, Fig. 26; †*Schistodesmus mamillatus* (Hislop, 1860): Modell (1969)^67^: p. 10	Karuni, 100 miles S.S.W. of Nagpur city, Hyderabad Territory, British India [near Karanji Village, 19.8567° N, 78.3141° E, Deccan Plateau, Telangana, India]^235^	Lectotype PIMB 946 [designated by Hartman et al., 2008]^145^

*Approximate age of the deposits = 67 Myr^145,146^.

**PIMB—Palaeo Invertebrate Mesozoic Bivalve numbers^145^ of the Natural History Museum, London, United Kingdom.

Tribe Lamellidentini Modell, 1942.

Genus *Arcidopsis* Simpson, 1900.

Type species: *Unio footei* Theobald, 1876 (by original designation)^70^.

Distribution: Endemic to the upper section of the Krishna Basin in Western Ghats, India^53,71^. At first glance, historical records from “Mysore”^72,73^ could be linked to the Upper Kaveri Basin near the city of Mysuru^53^ but are more likely to be attributed to the former State of Mysore, which also covered part of the Upper Krishna Basin.

Comments: This monotypic genus with its single species, *A. footei* (Table 1 and and Fig. 4a), was placed within the Unionidae *incertae sedis*^1,53^ but later it was transferred to the Lamellidentini^2^. The DNA sequences of this taxon are yet to be generated, and its fossil records are unknown.

Genus *Lamellidens* Simpson, 1900 [= *Velunio* Haas, 1919 **syn. nov.**; type species: *Unio velaris* Sowerby, 1868 (by monotypy)^74,75^].

Type species: *Unio marginalis* Lamarck, 1819 (by original designation)^70^.

Distribution: Indian and Western Indochina subregions: widespread from Indus River in Pakistan^60^ through India, Sri Lanka^57,61,76^, Nepal^61,62^, Bhutan^77^, and Bangladesh^57^ to Salween River in Myanmar^56^ and southwestern Yunnan in China. *Lamellidens candaharicus* (Hutton, 1849) [= *L. rhadinaeus* Annandale & Prashad, 1919 **syn. nov.**], the westernmost species of this genus, was discovered from the endorheic Sistan/Helmand River drainage in eastern Iran and Afghanistan^78^.

Comments: This genus contains 12 recent species, nine of which occur on the Indian Subcontinent and three in the Western Indochina (Table 1 and Fig. 3a–h). In this study, we provisionally delineate these species to two informal groups, which are largely supported by our phylogenies (Supplementary Figs. 1–2, Fig. 2 and Table 1). The *corrianus*-group contains four species usually having a more or less elongated shell, while the *marginalis*-group joins species with somewhat ovate or rounded shell. Conversely, the shell outline itself cannot be used for diagnostic purposes even between the two species groups, as the shell shape of taxa in this genus is extremely variable, and multiple intermediate forms do occur, e.g. those in *Lamellidens marginalis* (Fig. 3f–g).

A new formal synonymy is proposed here for several nominal taxa (Table 1). Based on morphological and biogeographic data, we transfer the nominal taxon *Physunio friersoni* Simpson, 1914 [new name for *Unio velaris* Sowerby, 1868]^79^ to *Lamellidens* and propose *L. friersoni* (Simpson, 1914) **comb. nov.** Hence, *Velunio* Haas, 1919 **syn. nov.**, a monotypic subgenus (section)^75^ of the genus *Physunio* Simpson, 1900, established for this taxon, should be considered a synonym of *Lamellidens*.

The nominal species *Unio groenlandicus* Mörch, 1868 was introduced based on a description and figure of Schröter^80,81^. This taxon cannot be attributed to Schröter^81^, because this author named it as “die breite Mahler-Muschel aus Grönland”, which is not a binomial name. Mörch stated that it “is *Unio testudinarius*, Spgl. (*U. marginalis*, Lam.), a common shell from Tranquebar and other places in British East Indies”^80^. However, we cannot link Schröter’s figure (pl. 9, Fig. 1)^81^ to a *Lamellidens* species due to the lack of pseudocardinal teeth. Hence, *Unio groenlandicus* is here considered a *nomen dubium*.

There are two older available names belonging to *Lamellidens*, i.e. *Unio testudinarius* Spengler, 1793 and *U. truncatus* Spengler, 1793 that were described from Tranquebar [Tharangambadi, 11.0292° N, 79.8494° E, Kaveri Basin, Tamil Nadu, India]^82^. Later, Haas redescribed these nominal taxa and illustrated the holotypes^83^. Based upon morphological examination, Haas considered *Lamellidens testudinarius* as the oldest available name for *L. marginalis*, and placed *L. truncatus* as a synonym of this species^83,84^. Furthermore, Haas synonymized the majority of nominal *Lamellidens* taxa under the name *L. testudinarius*^84^. However, this concept was largely ignored by subsequent researchers^1,2,57^. The assigment of these nominal taxa to certain species is not straghtforward. Morphologically, the holotype of *Lamellidens testudinarius* is an ovate shell^83^ that could be something from the *marginalis*-group, e.g. *L. marginalis*, *L. mainwaringi* or *L. jenkinsianus*. In its turn, the holotype of *Lamellidens truncatus* represents a narrower, elongated shell^83^ that looks either like *L. corrianus* or even the recently described *L. unioides*. Here, we prefer to consider these nominal species as *taxa inquirenda* but their identity will be clarified in the future based on molecular analyses of topotype samples from Tamil Nadu.

The three earliest fossil members of this genus were described from the Late Cretaceous Intertrappean Beds of the Deccan Plateau in India, i.e. †*Lamellidens carteri* (Hislop, 1860), †*L. deccanensis* (J. Sowerby in Malcolmson, 1840) **comb. nov.**, and †*L. vredenburgi* Prashad, 1921 (Table 2 and Supplementary Note 2). There are several fossil species of *Lamellidens* from Miocene to Pliocene deposits (mostly the Siwalik Group^64^) in India, Pakistan, Nepal, and Myanmar^66–68,85^.

Tribe Parreysiini Henderson, 1935.

Genus *Parreysia* Conrad, 1853.

Type species: *Unio multidentatus* Philippi, 1847 (by original designation)^86^.

Distribution: Indian Subregion: from Indus River in Pakistan^60^ through India, Sri Lanka^57,61,76^, Nepal^61,62^, and Bangladesh^57^ to coastal drainages of the Rakhine State of Myanmar^23^.

Comments: This genus contains six recent species endemic to the Indian Subcontinent (Table 1 and Fig. 5a–g). Here, we delineate these species to three informal groups (Fig. 2 and Table 1). The *keralaensis*-group contains *Parreysia keralaensis* **sp. nov.** only (Fig. 5a,b). This new species represents the most distant phylogenetic lineage within the genus (Fig. 2). The *corrugata*-group comprises four species that are phylogenetically and morphologically close to each other, representing a species complex (Table 1 and Fig. 5c–f). Our time-calibrated phylogeny indicates that the radiation within this group occurred during the Miocene (Fig. 2). Finally, the *rajahensis*-group contains a single species, *Parreysia rajahensis* (Lea, 1841). Although the DNA sequences of this species are not available, it probably represents a distant phylogenetic lineage due to a number of specific conchological features such as very thick, triangular shell and massive hinge plate (Fig. 5g).

The synonymy of *Parreysia* taxa is revised here (Table 1 and Supplementary Note 1). The nominal taxon *Parreysia robsoni* Frierson, 1927 [holotype NHMUK 1965150; type locality: Black River, North Carolina]^87,88^ cannot be linked to the Indian fauna, and it is considered here as a junior subjective synonym of *Fusconaia masoni* (Conrad, 1834) (Ambleminae) based on morphological features.

The three earliest fossil species belonging to this genus were discovered from the Late Cretaceous Intertrappean Beds of the Deccan Plateau in India, i.e. †*Parreysia imbricatus* (Hislop, 1860) **comb. nov.**, †*P. malcolmsoni* (Hislop, 1860), and †*P. mamillatus* (Hislop, 1860) **comb. nov.** (Table 2 and Supplementary Note 2). There are several fossil species in this genus that were described from Miocene to Pliocene deposits (mostly the Siwalik Group^64^) in India, Pakistan, Nepal, and Myanmar^65–69^.

*Parreysia keralaensis* Bolotov, Pasupuleti & Subba Rao **sp. nov.**

Figure 5a,b, Supplementary Figs. 3–6, Supplementary Table 2.

LSID: http://zoobank.org/urn:lsid:zoobank.org:act:627CB4BE-CD22-495A-8FDD-55F45D971CCD.

Type material: Holotype No. FBRC ZSI 1007-a (RCB2) [shell length 50.0 mm, shell height 33.5 mm, shell width 23.8 mm; reference *COI* sequence acc. no. KJ872811], Periyar River (downstream), 10.11° N, 76.37° E, Aluva, Kerala, India, 17.01.2014, R. Pasupuleti leg. Paratypes: Six specimens [museum lot No. FBRC ZSI 1007; specimen codes RCB3, RCB4, RCB5, RCB8, RCB9, and RCB12] from the type locality, 17.01.2014, R. Pasupuleti leg.; two specimens [museum lot No. FBRC ZSI 1006; specimen codes RNB1 and RNB2] from Periyar River (upstream), 10.06° N, 76.78° E, Neriamangalam, Kerala, India, 01.12.2014, R. Pasupuleti leg.; one specimen [museum lot No. FBRC ZSI 1223; specimen code RPC10] from Achankovil River, 9.25° N, 76.83° E, Pampa River basin, Kizhavalloor, Kerala, India, 03.09.2014, R. Pasupuleti leg. Reference *COI* sequences and shell measurements of the type series are given in Supplementary Table 2. The type series is deposited in FBRC ZSI (Hyderabad, Telangana, India).

Etymology: The new species name is dedicated to the Kerala State of India, in which it was collected.

Diagnosis: The new species can be distinguished from other *Parreysia* taxa by having a prominent, massive, rounded umbo and a specific wave-like sculpture over the umbo or through the entire shell surface (Fig. 5a,b and Supplementary Fig. 3). Additionally, it represents the most distant phylogenetic lineage within the genus (Fig. 2).

Description: Medium-sized mussel: shell length 34.8–59.1 mm, shell height 23.7–38.2 mm, shell width 15.1–28.8 mm (*N* = 10; Supplementary Table 2). Shell thick, of triangular or rounded shape, slightly inequilateral; ventral margin slightly convex; dorsal, anterior, and posterior margins rounded. Umbo massive, prominent, elevated. Shell sculpture well developed, with specific wave-like ridges, covering the umbonal region or the entire shell. Most specimens share weak corrugate plications posteriorly. Periostracum brown to dark orange with green tinge. Nacre white, with yellowish or pinkish tinge, shining. Hinge plate rather narrow. Left valve with two curved lateral teeth and two strongly indented pseudocardinal teeth. Right valve with one curved lateral tooth and one massive, indented pseudocardinal tooth with a small auxiliary tooth. Anterior adductor scar rounded and deep, posterior adductor scar rounded and very shallow. Umbonal cavity very deep.

Distribution: Periyar and Pampa basins, Kerala, Southwestern India.

Parreysiinae *incertae sedis.*

Genus *Balwantia* Prashad, 1919.

Type species: *Anodonta soleniformis* Benson, 1836 (by original designation)^89^.

Distribution: Upper Brahmaputra and Upper Barak (Dhaleswari) basins, India^89–91^.

Comments: This monotypic genus (Table 1 and Fig. 5h) was long thought to be a synonym of *Solenaia* Conrad, 1869 based on a similar ultra-elongated shell shape^1,57^. The latter genus was recently revised with separation of several genera such as *Solenaia* s. str.^27,92^, *Parvasolenaia* Huang & Wu, 2019^93^, *Koreosolenaia* Lee et al., 2020^37^, and *Sinosolenaia* Bolotov et al., 2021^94^. The first genus is a member of the tribe Contradentini^27,92^, while the others belong to the Gonideini^37,93,94^. Bolotov et al. restored *Balwantia* and placed *B. soleniformis* within the Contradentini together with two recently described species from Myanmar, having an ultra-elongated shell shape^23^. However, *B. soleniformis* shares unhooked glochidia and carries larvae in all the four gills (tetragenous brooding)^89^, and, hence, cannot be placed in the Contradentini^27,95^. Pfeiffer et al. considered it a monotypic genus, which may belong to the Parreysiinae^27^. Here, we place *Balwantia* as Parreysiinae *incertae sedis* because of the lack of available DNA sequences. Fossil records of this genus are not available.

## Doubtful and uncertain freshwater mussel taxa linked to India

In this section, we present a morphology-based overview of several nominal taxa, which were described by Constantine S. Rafinesque^96^. Subsequent researchers largely ignored these taxa as “indeterminate Unionidae” and even as “the worthless fabrications of Rafinesque” because of very poor and incomplete descriptions^97,98^. In the body of available literature on the types of Unionidae described by Rafinesque^99–103^, any mention of the type series for his Indian taxa is absent. Furthermore, we were unable to locate the current whereabouts of these types neither in European museums nor in those in the USA (including the ANSP Malacology Collection database; http://clade.ansp.org/malacology/collections). Perhaps, the type lots have been sold to a private collector(s), because in the introduction of that paper Rafinesque offered for sale all the type shells described there^96^. Hence, the types are probably lost. Therefore, our decisions and comments are based exclusively on the original descriptions. Taxa, the protologues of which lack diagnostic features for reliable taxonomic identification, are considered here as *nomina dubia*.

A complete reappraisal of Rafinesque’s nominal taxa linked to India^96^ is given in Supplementary Note 3, while a brief summary of our taxonomic decisions is presented here. *Diplasma marginata* Rafinesque, 1831 is considered a *nomen dubium*, because its type locality is uncertain (River Tennessee or Hindostan) and the identity is unclear. Three more nominal species cannot be identified with certainty based on the original descriptions and are also considered *nomina dubia*: *Diplasma similis* Rafinesque, 1831 (type locality: River Ganges); *Diplasma* (*Hemisolasma*) *vitrea* Rafinesque, 1831 (type locality: River Jellinghy in Bengal [approx. 23.4356° N, 88.4905° E, Jalangi River, West Bengal, India]); and *Lampsilis fulgens* Rafinesque, 1831 (type locality: River Ganges)^96^. Hence, the associated genus- and family-group names such as *Diplasma* Rafinesque, 1831 (type species: *Diplasma marginata*^98^), *Hemisolasma* Rafinesque, 1831 (type species: *Diplasma* (*Hemisolasma*) *vitrea*^101,104^), Diplasminae Modell, 1942^105^, and Hemisolasminae Starobogatov, 1970^106^ also become *nomina dubia*.

Based on the original descriptions^96^, *Lampsilis argyratus* Rafinesque, 1831 (type locality: River Ganges) and *Diplasma* (*Hemisolasma*) *striata* Rafinesque, 1831 (type locality: River Jellinghy in Bengal [approx. 23.4356° N, 88.4905° E, Jalangi River, West Bengal, India]) are considered here as junior synonyms of *Indonaia caerulea* (Lea, 1831) and *I. rugosa* (Gmelin, 1791) **comb. nov.**, respectively (Table 1). Furthermore, the diagnostic features, mentioned in the protologue^96^, clearly indicate that the monotypic genus *Loncosilla* Rafinesque, 1831 with its type species *Loncosilla solenoides* Rafinesque, 1831 is not a unionid mussel but a freshwater clam of the family Pharidae. A more in-depth comparative analysis using available taxonomic works on freshwater Pharidae^107,108^ allowed us to propose the formal synonymy as follows: *Novaculina* Benson, 1830 [= *Loncosilla* Rafinesque, 1831 **syn. nov.**] and *Novaculina gangetica* Benson, 1830 [= *Loncosilla solenoides* Rafinesque, 1831 **syn. nov.**] (Pharidae: Pharellinae).

Additionally, we would note on the enigmatic nominal taxon *Unio digitiformis* Sowerby, 1868 [holotype NHMUK 1965199; type locality: India] that shares an ultra-elongated shell with pointed posterior margin. Different authors placed it within different genera such as *Nodularia* Conrad, 1853, *Lanceolaria* Conrad, 1853, and *Indochinella* Bolotov et al., 2018^35,84,109,110^. Haas stated that this species is certainly not a member of the Indian fauna^84^. Based upon a morphological examination of the holotype, we found that it conchologically corresponds to *Diplodon parallelopipedon* (Lea, 1834) (Hyriidae: Hyriinae), a South American species, which is known to occur in the Paraná Basin and coastal drainages of Uruguay^1,2^. Hence, its type locality was given in error. The formal synonymy is proposed here as follows: *Diplodon parallelopipedon* [= *Unio digitiformis* Sowerby, 1868 **syn. nov.**].

## Discussion

### Taxonomic richness and endemism of Oriental freshwater mussels

The Indian Subcontinent houses a rather taxonomically poor fauna of the Unionidae, which contains 25 species belonging to three Gondwanan tribes (Indochinellini, Lamellidentini, and Parreysiini) and one subfamily, the Parreysiinae. All these species are endemic to the region, except for *Lamellidens nongyangensis* Preston, 1912, a local population of which was recorded in Lake of No Return (Nongyang Lake) near the boundary between India and Myanmar. Our novel results confirm the conclusion of Bolotov et al.^19^ that the Unionidae faunas of the Indian and Western Indochina subregions share almost 100% level of endemism at the species level and that multiple records of Indian species in Myanmar^57,110–113^ were based on erroneous identifications. Furthermore, the tribe Parreysiini and the genera *Arcidopsis*, *Balwantia*, and *Parreysia* are unknown beyond the Indian Subregion.

The taxonomic richness of the Unionidae fauna in Western Indochina is 2.5 times higher compared with that on the Indian Subcontinent, with more than 60 species, but it represents an amalgam of the original Gondwanan taxa (Indochinellini, Lamellidentini, and Leoparreysiini), and the Paleogene immigrants from the Sundaland (Contradentini and Pseudodontini)^19,22,23,35,56^. The genera *Indochinella* Bolotov et al., 2018, *Pseudodon* Gould, 1844, *Radiatula* Simpson, 1900, *Trapezidens* Bolotov, Vikhrev & Konopleva, 2017, and *Yaukthwa* Konopleva et al., 2019 are endemic to the Western Indochina Subregion^22,23,35,53,56,114^. Though *Leoparreysia* Vikhrev, Bolotov & Kondakov, 2017 was also considered among these endemic clades^23,35,56^, morphology-based surveys indicate that it may contain at least two species east of the Salween – Mekong drainage divide. First, *Leoparreysia subcircularis* (Brandt, 1974) [type locality: Mekong River between Takek and Nakon Panom, Laos]^115^ was recently transferred from Contradentini to Leoparreysiini^27^. Second, *Leoparreysia superstes* (Neumayr, 1899) **comb. nov.** [type locality: Erhai Lake, Upper Mekong Basin, Yunnan, China]^116^ conchologically corresponds to the Leoparreysiini and is moved from *Rhombuniopsis* Haas, 1920 (Unioninae: Unionini) to *Leoparreysia* here. However, the generic placement of both species, mentioned above, is yet to be confirmed by means of the DNA-based approach.

The Indian cementing bivalve *Pseudomulleria dalyi* (Smith, 1898) was considered a possible Gondwanan relict^117^. Traditionally, this enigmatic “freshwater oyster” was placed in the Etheriidae based on morphological criteria^57,84,110,118,119^ but phylogenetic reconstructions using a single available *COI* sequence of this taxon (acc. No. AF231750) repeatedly indicated that it is a unionid species belonging to the Parreysiinae^120–122^. Its close affinities with the Unionidae were previously assumed based on anatomical surveys^118,123^. Conversely, Graf & Cummings considered this *COI* sequence as potentially problematic due to the discordance of its phylogenetic position with morphological data^104^ and returned *Pseudomulleria dalyi* to the Etheriidae^1^. The latter concept of *Pseudomulleria* is accepted in the most recent global checklist of freshwater mussel taxa^2^. Although the *COI* sequence under discussion seems to be correct, we did not include this taxon to the current list of the Indian Parreysiinae, because a final solution on its family-level placement requires an expanded set of DNA sequences and needs further research efforts^2^.

We show that several highland areas of the Indian Subcontinent harbor endemic taxa of freshwater mussels with restricted ranges (Table 1). As it was expected^117^, rivers flowing from the Western Ghats share the highest proportion of regional endemic species such as *Arcidopsis footei* (Tunga, Ghataprabha, and Koyna rivers, Upper Krishna Basin^71,124^), *Indonaia bonneaudii* (Karli River), *I. cylindrica* **comb. nov.** (Upper Krishna Basin), *Parreysia keralaensis* **sp. nov.** (Periyar and Pampa basins), and *Pseudomulleria dalyi* (Tunga and Bhadra rivers, Upper Krishna Basin^124,125^). Further, rivers in Assam and northeastern Bangladesh house at least three narrowly endemic taxa, i.e. *Balwantia soleniformis* (Upper Brahmaputra and Upper Barak basins), *Indonaia involuta* (Upper Brahmaputra and Surma basins), and *Lamellidens friersoni* **comb. nov.** (Upper Brahmaputra Basin). Finally, *Parreysia rajahensis* seems to be endemic to the Narmada River^97^. Hence, these freshwater systems should be considered of highest priority areas for freshwater mussel conservation at the national and global levels.

It was assumed that the Western Ghats could have served as a refugium for the autochthonous Gondwanan fauna during the Deccan eruptions^126^. At first glance, our data on the taxonomic diversity and endemism of freshwater mussels agree with this hypothesis. This mountain range represents a major evolutionary hotspot for a plethora of taxa with possible Gondwanan affinities such as scorpions^126^, freshwater gastropods^127^, freshwater fish^128,129^, frogs^130–133^, and evergreen trees^134^.

The Andaman and Nicobar archipelagoes, being a union territory of India, are located on a separate tectonic platelet, which is confined to the western margin of the Sunda Plate^38^. These islands may therefore harbor a specific freshwater mussel assemblage that should be different from those on the Indian Subcontinent and Burma Terrane. A single nominal taxon, *Alasmodonta nicobarica* Mörch, 1872, was described on the basis of one shell from the Nicobar Islands^135^. Based upon the original description^135^, this shell is irregularly oval, convex, with irregular growth lines; dorsal margin slightly arched, anterior margin narrowed and rounded, ventral margin slightly concave, posterior margin narrowed and slightly prominent; shell color is olive, with darker bandages and numerous dark green rays; umbo is not prominent, eroded; pseudocardinal teeth are almost completely absent, lateral teeth are lamellar. Simpson placed this nominal taxon in the genus *Pseudodon* sensu lato^70^ based on Mörch’s comments in the protologue^135^ but later transferred it to *Pletholophus* Simpson, 1900 with respect to the expert opinion of Haas, who has examined the holotype of *Alasmodonta nicobarica*^79^. Currently, it is considered a synonym of *Pletholophus tenuis* (Griffith & Pidgeon, 1833) (Unioninae: Cristariini), an East Asian species^2^. However, if its type locality is stated correctly, it cannot be assigned to *Pletholophus tenuis* from a biogeographical point of view^35^. At first glance, it may be a member of the genus *Monodontina* Conrad, 1853 (Gonideinae: Pseudodontini). This genus can be distinguished from other taxa by having an ovate or rounded shell and weakly developed pseudocardinal teeth^36^, which aligns with the original description of *Alasmodonta nicobarica*. The *Monodontina* taxa sometimes share green rays through the periostracum. The genus *Monodontina* is known to occur in southern Myanmar (Lenya River), southern Thailand, Chao Phraya and Mekong basins, and throughout Malaysia and the Greater Sunda Islands^23^, and, hence, could theoretically be found on the Nicobar Islands. Here, we propose *Monodontina nicobarica* (Mörch, 1872) **comb. nov.** as a preliminary taxonomic hypothesis that needs to be checked by means of a DNA-based approach. The Great Nicobar Island with its numerous rivers and streams flowing through primary tropical forests could indeed house some interesting freshwater mussel taxa and must be a focus of future collecting efforts.

### Gondwanaland origin and diversification of the Parreysiinae

Our earlier biogeographic scenario on the origin and diversification of the Unionidae^19^ suggested that the MRCA of Parreysiinae has originated in Western Indochina in the Late Jurassic (ca. 150 Myr), with subsequent dispersal of descendants into Africa in the mid-Cretaceous (ca. 95 Myr) and into India in the Paleocene (ca. 60 Myr). At that time, we did not consider the Burma Terrane as a Gondwanan fragment and used a very restricted set of Indian taxa. Hence, our scenario predicted a Laurasian origin of the Parreysiinae and their westward expansion to East Gondwana starting more than 100 Myr ago that, as Pfeiffer et al. noted^51^, weakly corresponds to modern paleogeographic reconstructions. The latter authors proposed an alternative hypothesis on the origin of the Parreysiinae^51^. Their scenario predicted that this subfamily clade originated in Western Eurasia, with subsequent expansions south to Africa, and east to India, Myanmar, and mainland Southeast Asia. Pfeiffer et al. placed these events in the Cenozoic^51^, after final contact of the Indian Subcontinent with the Eurasian Plate (i.e. since the mid-Eocene^42,45^), based on multiple Miocene fossils of three Parreysiinae lineages (Coelaturini, *Parreysia*, and *Lamellidens*) on Gondwanan fragments. Using these fossils as time calibrations, Ortiz-Sepulveda et al. proposed an additional scenario on African taxa that predicted the Early Miocene origin of the Coelaturini in Eurasia followed by their Early to Middle Miocene expansion into Africa, roughly coinciding with the closure of the Tethys Sea^31^. Anyway, all the scenarios, outlined above, do not consider modern plate tectonic reconstructions and, though substantially differ by timeframe, support the so-called “Out-of-Asia” model^136^.

**Figure 6:**
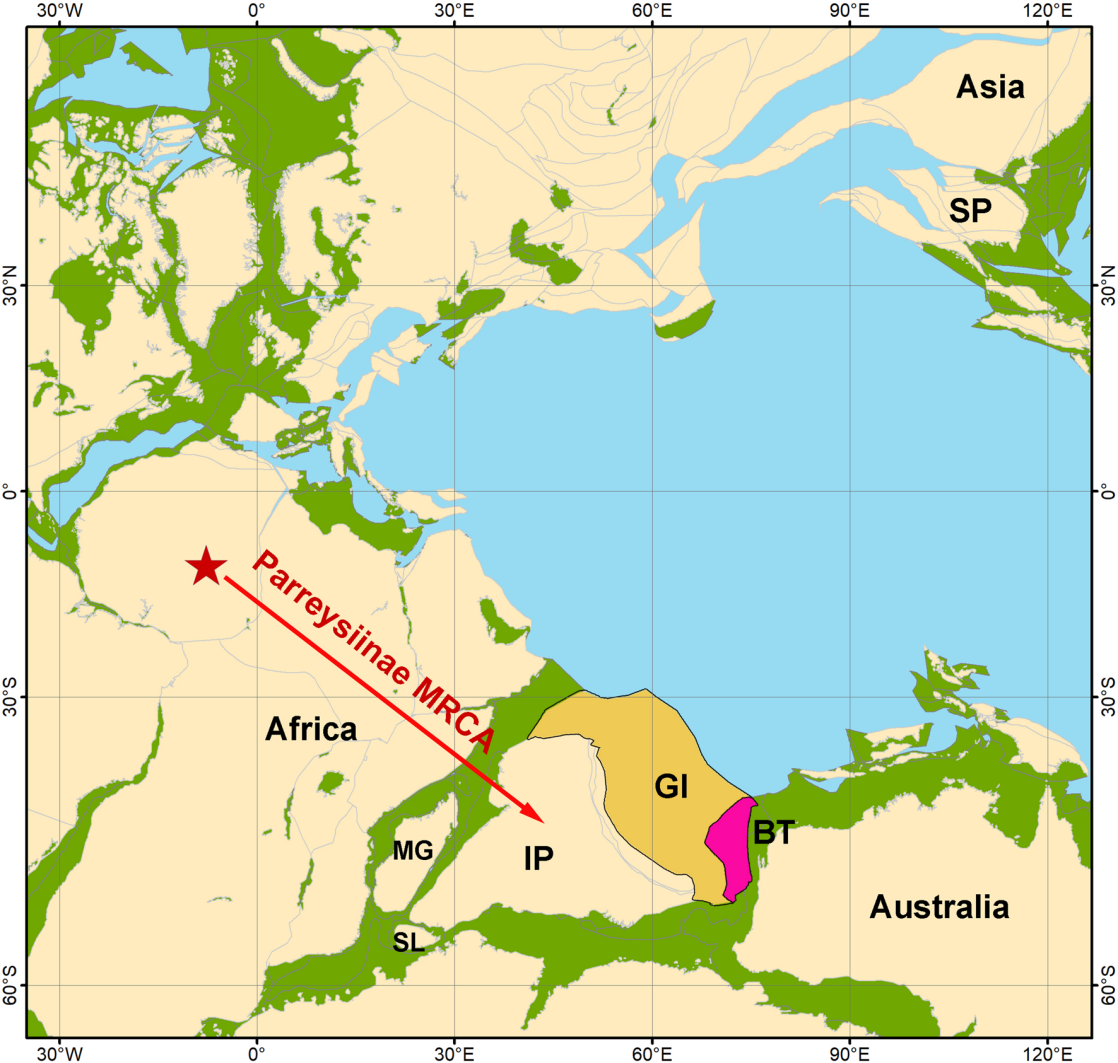
Proposed scenario of the trans-Gondwanan expansion of the Parreysiinae MRCA (red arrow) in the Middle Jurassic (170–165 Myr). We assume that the MRCA of this subfamily migrated through freshwater systems of the African Plate and/or Arabia to an ancient landmass, containing the Indian Plate (with Greater India) and Burma Terrane. Red star indicates fossil records of the earliest African crown-group unionid mussels from Mid- to Late Jurassic deposits in Niger, i.e. †*Coactunio iguallalensis*, †*Rostrunio lapparenti*, and †*Tuaregunio agadesensis*^138^. *IP* Indian Plate, *BT* Burma Terrane, *GI* Greater India, *SP* Sunda Plate (with the Indochina Block and Sibumasu Terrane), *SL* Sri Lanka, *MG* Madagascar. Color filling is as follows: Burma Terrane (pink), Greater India (light orange), modern land (light yellow), proposed ancient land (light green), and ocean surface (light blue). The paleo-map was reconstructed using GPlates v. 2.3 (https://www.gplates.org)^205^ and corresponding data sets^206–209^. Reconstruction of the Greater India in Gondwana follows published works^47,139^ with additional modifications according to our biogeographic reconstructions. (Maps: Mikhail Yu. Gofarov and Ivan N. Bolotov).

Here, we present an updated reconstruction of the origin and diversification of the Parreysiinae based on our novel biogeographic modeling, expanded paleontological data set, and the newest tectonic and paleomagnetic reconstructions^39–42,48,50,137^. Our new “Out-of-India-&-Burma” scenario indicates that this subfamily originated in East Gondwana in the Late Jurassic. While Late Triassic or Early Jurassic African Unionidae are unknown, the Mid- to Late Jurassic deposits (age from 170 to 145 Myr) house a species-rich freshwater mussel assemblage, which contains a number of Unionidae and Margaritiferidae taxa^138^. The ancestors of these groups most likely arrived to East Gondwana from Laurasia through the joined African Plate and Arabia. Several fossil freshwater mussel taxa from the Irhazer Group deposits (Mid- to Late Jurassic) of Niger resemble modern Unionidae in the hinge structure, e.g. the monotypic genera †*Coactunio* Van Damme & Bogan, 2015, †*Rostrunio* Van Damme & Bogan, 2015, and †*Tuaregunio* Van Damme & Bogan, 2015^138^. These taxa were considered the earliest members of the modern crown-group of the Unionidae in Jurassic Africa^138^. In our opinion, these rare fossils could be linked to the MRCA or a steam group of the Parreysiinae based on the hinge structure. The initial breakup of East Gondwana from West Gondwana started approximately 160 Myr, separating the Indian Plate together with its proposed satellites from continental Africa^45,139^. Hence, the Parreysiinae MRCA most likely colonized India earlier (Fig. 6).

**Figure 7:**
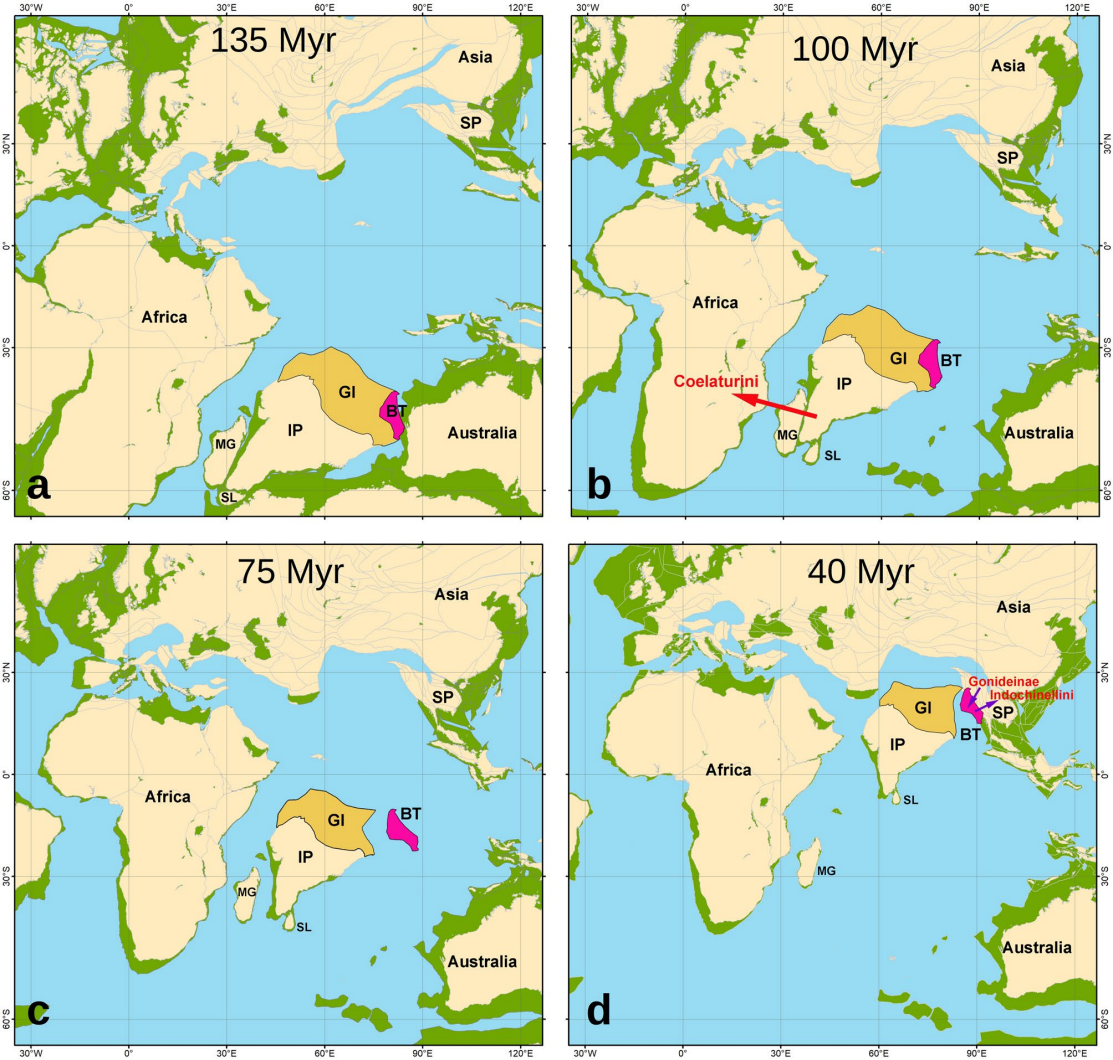
Proposed scenarios of tectonic evolution of the Indian Plate and Burma Terrane with respect to our time-calibrated phylogenetic reconstruction and statistical biogeographic models for the freshwater mussel subfamily Parreysiinae (see Fig. 2 for detail). The paleo-maps are as follows: (**a**) Early Cretaceous (135 Myr): unionid mussels have gone extinct in continental Africa but survived on an East Gondwanan fragment containing the Indian Plate (with Greater India) and Burma Terrane, where the first split in the Parreysiinae did occur, i.e. the separation of the Lamellidentini; (**b**) mid-Cretaceous (100 Myr): colonization event of the Coelaturini MRCA (red arrow) to continental Africa, suggesting direct contact between the Indian Subcontinent and African Plate, probably through Madagascar; the Indian Plate and Burma Terrane are still connected through the Greater India; (**c**) Late Cretaceous (75 Myr): final separation of the Burma Terrane from the Indian Plate, probably by a partial submergence of the Greater India; both the landmasses served as insular “biotic ferries”, carrying Gondwanan biota to Asia; and (**d**) Late Eocene (40 Myr): Burma Terrane—Asia collision, leading to the expansion of the Mekong’s Indochinellini MRCA to the Sundaland Subregion and a colonization event of the *Pseudodon* and *Yaukthwa* MRCAs (Gonideinae: Pseudodontini and Contradentini)^23^ to the Burma Terrane (purple arrows). *IP* Indian Plate, *BT* Burma Terrane, *GI* Greater India, *SP* Sunda Plate (with the Indochina Block and Sibumasu Terrane), *SL* Sri Lanka, *MG* Madagascar. Color filling is as follows: Burma Terrane (pink), Greater India (light orange), modern land (light yellow), proposed ancient land (light green), and ocean surface (light blue). The paleo-maps were created using GPlates v. 2.3 (https://www.gplates.org)^205^ and corresponding data sets^206–209^, with additional modifications according to a set of novel tectonic and paleomagnetic models^39–42,48,137,139^ and our biogeographic reconstructions. (Maps: Mikhail Yu. Gofarov and Ivan N. Bolotov).

Our scenario further suggests that the earliest diversification in the subfamily occurred on an ancient landmass, containing the Indian Plate and Burma Terrane, which were joined through the Greater India, in the Early Cretaceous^47,139^ (Fig. 7a). During that period, two large clades, i.e. Lamellidentini (Fig. 2: Event I) and Parreysiini + Leoparreysiini (Fig. 2: Event II) were separated. The splits, outlined above, coincided with a complete disappearance of unionids and margaritiferids in the Early Cretaceous deposits of continental Africa, probably reflecting a major extinction event^138^. Though it roughly coincides with the global Tithonian extinction event^138^, the post-Jurassic disappearance of naiads in Africa could also be linked to active development of a large system of rifts, leading to intercontinental marine transgressions^140–142^. Thereby we could assume that an ancient landmass, which consisted of the modern Indian Plate, Greater India, and Burma Terrane^39,40,42,48,137^, played a significant role as a refugium for freshwater mussels in the Early Cretaceous. Perhaps some geographic barriers on this landmass such as mountain ranges drove the early macroevolution of the Parreysiinae, as it was suggested for the diversification patterns in the Hyriidae^18^.

Our modeling reveals that a re-colonization event of the Parreysiinae from the Indo-Burma refugium into Africa most likely occurred in the mid-Cretaceous (Fig. 7b). This dispersal was followed by a vicariance event (mean age = 98 Myr) that lead to the origin of the African tribe Coelaturini (Fig. 2: Event III). A similar mean age was obtained for the split between the African *Parachanna* and Oriental *Channa* clades of snakehead fishes (Channidae) using a set of crown fossil calibrations^129^. The sister family Aenigmachannidae, a unique subterranean lineage from Western Ghats, separated from the Channidae approximately 110 Myr ago^129^. The pattern, outlined above, predicts a hypothetical land bridge between the Indian Plate and Africa nearly 100–110 Myr ago, probably through Madagascar^45,143^. Conversely, India could have reestablished biotic connections with Africa during its collision with the Kohistan–Ladakh Arc along the Indus Suture in the Late Cretaceous (ca. 85 Ma)^45^, although this geological event postdates our divergence age for the Coelaturini. Briggs assumed that India always remained close to Africa and Madagascar during its northward motion^144^. Our results, however, suggest that the Indian Plate was linked to Africa sometime in the mid-Cretaceous but this connection was lost afterwards.

Three subsequent splits in the subfamily Parreysiinae reflect the segregation of the Indian Plate and Burma Terrane during the Late Cretaceous (mean age interval 96 to 74 Myr; Fig. 2: Events IV, V, VI). There is no evidence of any connection between these landmasses during a nearly 50-Myr period (74 to 26 Myr), starting near the Campanian—Maastrichtian boundary and covering the entire Paleogene epoch (Fig. 7c). Our review of available fossils from the Deccan Intertrappean Beds reveals that members of *Indonaia*, *Lamellidens*, and *Parreysia* were presented on the Indian Plate^145^ right before the Cretaceous – Paleogene (K-Pg) boundary^146^ (Table 2 and Supplementary Note 2). These paleontological findings support our ancestral area reconstruction indicating the Indian origin of these genera. Van Damme et al. noted that Deccan fossils could also belong to the Hyriidae because they often share a crenate or wavy ventral margin^138^. However, such “plicate” forms could independently evolve in different unionoid families, e.g. the Margaritiferidae (*Margaritifera marrianae* Johnson, 1983^147^ and *Pseudunio flabellatus* (Goldfuss, 1837)^148^) and Unionidae (genera *Amblema* Rafinesque, 1820^149^, *Tritogonia* Agassiz, 1852^150^, and others).

The Burma Terrane collided with the Sunda Plate in the Late Eocene (mean age = 38 Myr) that is reflected by the dispersal event of the Mekong’s Indochinellini from the terrane to mainland Asia (Figs. 7d, 2: Event VII). During the same period, members of the tribes Contradentini and Pseudodontini, belonging to the subfamily Gonideinae, colonized the Burma Terrane from Asia (Sunda Plate) that leads to the endemic *Pseudodon* and *Yaukthwa* radiations in Western Indochina^23^. The Sundaland itself most likely represents an ancient evolutionary hotspot for the Gonideinae, because two endemic, deeply divergent tribes were recently discovered from Borneo, i.e. the Ctenodesmini Pfeiffer, Zieritz, Rahim & Lopes-Lima, 2021 and Schepmaniini Lopes-Lima, Pfeiffer & Zieritz, 2021^28^. Though these Bornean clades are yet to be involved into any time-calibrated phylogeny, their phylogenetic position (sister to the Contradentini + Rectidentini and to Pseudodontini, respectively) undoubtedly indicates a Late Mesozoic separation^28^.

A few species-level splits discovered in the genera *Indonaia* and *Lamellidens* indicate that the first re-connection of the Indian Plate and Burma Terrane did occur at the Oligocene – Miocene boundary (mean ages 26 to 24 Myr). Several additional faunal exchanges between these landmasses during the Miocene (mean ages 12 to 8 Myr), most likely reflecting river (stream) capture events, were also uncovered by our phylogeny. These range expansions could be traced in multiple fossil records of *Indonaia*, *Lamellidens*, and *Parreysia* species from Miocene deposits throughout Pakistan, India, Nepal, and Myanmar^65–69^. Perhaps, the exchanges between freshwater mussel faunas of the Indian Subcontinent and surrounding landmasses during the Miocene were triggered by humid and warm climatic episodes, as it was shown for freshwater gastropods^127,151^ and amphibians^152^.

Interestingly, none of the unionid mussels seems to follow the so-called “Into India” scenario, though this pattern frequently occurs in Indian freshwater gastropods^127,153,154^, frogs^155,156^, and other animals. In contrast, our biogeographic models trace multiple “Into Burma” expansion events from India and Sundaland, starting since the Burma Terrane – Asia collision in the Late Eocene.

### Burma Terrane as a second “biotic ferry” from Gondwana to Asia

There are multiple evidences that the Indian Plate has served as a “biotic ferry”, transferring a derivative of the aboriginal Gondwanan biota to Asia^44,157–159^. The iconic examples of taxa that are thought to have arrived to Asia by this way were discovered among caecilians^160,161^, frogs^132^, freshwater fishes^129,162^, freshwater crabs^163^, centipedes^164^, scorpions^126^, tarantulas^165^, and various plants^166,167^. Our study reveals that unionid mussels, a primarily freshwater group, the dispersal of which requires direct links between landmasses, should surely be added to the list of “passengers” that have travelled through the Tethys Ocean on this tectonic block. Furthermore, we show that the Burma Terrane could be considered a separate “biotic ferry” that also carried members of Gondwanan biota to Asia (Fig. 7a–d). The high degree of endemism discovered in freshwater mussels on the Burma Terrane (and on the Indian Subcontinent as well) reveals that the Gondwanan “biotic ferries” have served as insular evolutionary hotspots, at least during the entire Paleogene (Fig. 7c). Our results support the hypothesis on insular endemism patterns (the so-called “endemic insularity syndrome”) discovered in the paleo-biota from the mid-Cretaceous Burmese amber^168–170^.

Earlier, it was suggested that several non-Indian Gondwanaland fragments such as the Burma and Lhasa terranes might have transferred Gondwanan lineages into Asia but any direct biogeographic evidence supporting this idea was not available^127^. The body of literature on this issue is still very limited, and a few available reports are based exclusively on paleontological data. First, a review of biogeographic affinities of numerous plant and animal taxa discovered in the mid-Cretaceous Burmese amber (ca. 100 Myr; near the Albian—Cenomanian boundary) reveals that this biota represents a selection of Gondwanan organisms and that the Burma Terrane could not have separated from East Gondwana before the Early Cretaceous^49^. From this perspective, ancestors of several secondary freshwater/estuarine and terrestrial groups of Mollusca discovered in Burmese amber such as †*Palaeolignopholas* piddocks (Pholadidae)^171^ and some land snail taxa (Diplommatinidae and Pupinidae)^172,173^ may have also arrived to Asia with the Burma Terrane. The discovery of a freshwater pond snail (Lymnaeidae) in this amber, however, could be linked to a long‐distance dispersal event^174^. Perhaps it was not a transoceanic dispersal as such but an expansion from the nearby Indian Subcontinent, because the Deccan Trap sedimentary sequence harbors a diverse and species-rich assemblage of fossil freshwater snails, containing the Lymnaeidae, Planorbidae, Pomatiopsidae, Succinidae, Thiaridae, Valvatidae, and Viviparidae taxa^175^. Second, on the basis of a comprehensive survey on the Eocene flora of Myanmar, the Burma Terrane was considered a Gondwanan fragment that collided with Asia in the Late Eocene (ca. 41 Myr) and facilitated floristic exchange between the terrane, Indian Plate, and Asian mainland^176^. The dating of the Burma Terrane—Asia collision recovered in this research aligns with our estimate of 38 Myr inferred from the time-calibrated phylogeny of the Parreysiinae.

## Conclusion

Our research presents the first DNA-based evidence that the Burma Terrane transferred an ancient derivative of Gondwanan biota to Asia, as India did. These results agree with a growing body of modern paleontological, tectonic, paleomagnetic, and geological research, supporting a Gondwanan origin of the Burma Terrane and its northward rafting through the Tethys Ocean^39–41,48,49,176^. Based on biogeographic patterns that were discovered in freshwater mussels (Unionidae: Parreysiinae), we propose that this terrane was a part of an ancient landmass, also containing the Indian Plate and Greater India, from the Middle Jurassic (ca. 160 Myr) to the terminal Cretaceous (ca. 75 Myr). Later on, during the Paleogene, the Burma Terrane was an isolated island that has collided with mainland Asia (Sunda Plate) in the Late Eocene (ca. 40 Myr). The biogeographic reconstruction presented here could be used as supplement to modern plate tectonic models, repeatedly indicating northward drifting of the Burma Terrane alongside the Indian Plate^40,177^. In general, our scenario of tectonic evolution of the region differs from other available scenarios^40^ by the position of the Burma Terrane in relation to that of the Indian Plate and Greater India.

From this perspective, mainland Southeast Asia represents a Late Eocene collision zone of two tectonic blocks (Burma Terrane and Sunda Plate), initially housing completely different biotas^176^. These blocks could roughly be delineated via the Sagaing Fault and along the northern part of the Tenasserim Range through the Three Pagodas and Ranong faults^178^. This unique pattern was largely overlooked until recently which sometimes lead to incorrect conclusions on the origin and diversification of certain taxa, e.g. onychophorans^179^. To avoid possible reconstruction failures, Western Indochina should be coded as a separate, Gondwana-derived ancestral area in statistical biogeographic and paleontological models. Furthermore, the origin and role of several geographic barriers linked to the collision zone such as the Isthmus of Kra^23^ and the Salween – Mekong drainage divide^35^ must be re-considered based on these new findings.

## Methods

### Data collection

Freshwater mussel samples were collected from various localities in India, Nepal, and Myanmar from 2012 to 2020. A small foot or mantle tissue snip from each specimen from Myanmar and Nepal was fixed with 96% ethanol immediately after collection^19,22,23^. For the Indian samples, hemolymph was preferred as the source of genomic DNA. The hemolymph samples (0.2 ml per one specimen) were collected using a standard approach^180^, and genomic DNA was isolated from 0.1 ml of fresh hemolymph using the NucleoSpin® Tissue Kit (Macherey–Nagel GmbH & Co. KG, Germany), following the manufacturer protocol. The partial sequences of the mitochondrial *COI*, *16S rRNA*, and the nuclear *28S rRNA* genes were generated using standard protocols described in our earlier works^19,53,56^. The *COI* sequences of samples from Nepal were generated using the LCO1490 and COIschneck primers pair^181^, while those from Indian samples were obtained with the standard Folmer’s primers^182^. Additional DNA sequences of Indian and African taxa were obtained from NCBI’s GenBank (Datasets 1 and 2).

The dry shell vouchers and ethanol-preserved complete specimens collected by us were deposited in the following collections: FBRC ZSI—Freshwater Biology Regional Centre, Zoological Survey of India, Hyderabad, India (samples from India); SMF—Senckenberg Museum, Frankfurt, Germany (samples from Nepal); and RMBH—Russian Museum of Biodiversity Hotspots, Federal Center for Integrated Arctic Research of the Ural Branch of the Russian Academy of Sciences, Arkhangelsk, Russia (samples from Myanmar). Additionally, we examined historical shell lots in NMNH—National Museum of Natural History, Smithsonian Institution, Washington, DC, United States of America; MCZ—Museum of Comparative Zoology, Cambridge, USA; NHMUK—British Museum of Natural History, London, United Kingdom; MNHN—Muséum National d’Histoire Naturelle, Paris, France; MSNG—Museo Civico di Storia Naturale di Genova, Genoa, Italy. Furthermore, the MUSSEL Project (MUSSELp) Database (http://mussel-project.uwsp.edu) was used as a reliable source of taxonomic, bibliographic, and morphological information on nominal taxa of freshwater mussels^1,2,183^.

### Phylogenetic analyses

To reconstruct multi-locus phylogeny of the Parreysiinae (3 codons of *COI* + *16S rRNA* + *28S rRNA*), we compiled an alignment with 61 unique species-level haplotypes, including four outgroup taxa, that were selected from 203 sequenced specimens (Datasets 1 and 2). The maximum likelihood and Bayesian phylogenies were calculated using IQ-TREE v. 1.6.12^184^ and MrBayes v. 3.2.7a^185^, respectively. The IQ-TREE^184^ analysis was run using an automatic identification of the best evolutionary models for each partition^186^ and an ultrafast bootstrap algorithm^187^ via an online server (http://iqtree.cibiv.univie.ac.at)^188^. The Bayesian analysis was performed through the CIPRES Science Gateway^189^. We assigned the best-fit evolutionary models to each partition based on the second-order Akaike information criterion (AICc) of MEGA7^190^ as follows: GTR + G (1st codon of *COI*); TN93 + G + I (2nd codon of *COI*); HKY (3rd codon of *COI*); GTR + G + I (*16S rRNA*); and GTR + G (*28S rRNA*). The MrBayes settings were as follows: two runs (each with 50,000,000 generations), four MCMC chains (three cold and one heated; temp = 0.1), sampling every 1000th generation, and a 15% burn-in.

### Species delimitation

Two species delimitation approaches were applied through available web-services, that is, the Poisson Tree Process (PTP) modeling (http://mptp.h-its.org)^191^ and ASAP (assemble species by automatic partitioning; https://bioinfo.mnhn.fr/abi/public/asap)^192^. As an input tree, we used the maximum likelihood *COI* phylogeny of the Parreysiinae inferred from IQ-TREE v. 1.6.12^184^. An initial alignment with 196 in-group *COI* sequences was compiled (Dataset 1). This alignment was converted to 173 unique haplotypes using an online sequence toolbox, FaBox v. 1.5 (https://birc.au.dk/~palle/php/fabox)^193^. Four Pseudodontini haplotypes were used as outgroup (Dataset 1). The IQ-TREE analysis was run through an online server (http://iqtree.cibiv.univie.ac.at)^184^ with settings as described above. Each species-level Molecular Operational Taxonomic Unit (MOTU), probably corresponding to a biological species, was checked with morphological criteria such as the shell shape, shell sculpture, umbo position, structure of pseudocardinal and lateral teeth, and shape of muscle attachment scars^56^. To link each MOTU to certain nominal species, the conchological features of available specimens were compared with the original taxonomic descriptions^23^.

### Divergence time estimation

Divergence ages were estimated using BEAST v. 2.6.3^194,195^. The time-calibrated phylogeny was reconstructed based on an external *COI* evolutionary rate (1.5E-9 substitutions per site per year)^196^. This rate was obtained using a comprehensive set of mitochondrial genome sequences and several reliable fossil calibrations for Unionidae taxa^196^ and largely agrees with earlier estimates of the *COI* molecular clock rate in freshwater mussels^147,197^. For BEAST runs, we used the same multi-locus dataset as for the IQ-TREE and MrBayes phylogenetic analyses (3 codons of *COI* + *16S rRNA* + *28S rRNA*). The molecular clock rate was assigned to the *COI* partition only. The HKY + G model was applied for each gene partition based on our earlier considerations^19^. We applied a strict clock algorithm with the Yule speciation process as the tree prior^198^. The BEAST runs were performed through the CIPRES Science Gateway^189^. Three independent BEAST runs were performed, each with 150,000,000 generations and tree sampling every 1000th cycle. The resulting log files were checked visually with Tracer v. 1.7.2^199^. The Effective Sample Size (ESS) values of all the parameters in the combined runs were found to be > 300 after a 50% burn-in. The final tree set was generated using LogCombiner v. 2.6.6^194,194^ with an additional re-sampling every 5,000th generation and an appropriate burn-in. The consensus tree was found with TreeAnnotator v. 2.6.6^194,194^.

### Ancestral area reconstruction

First, we reconstructed possible ancestral areas using data on the distribution of Parreysiinae species throughout tectonic plates as follows: African Plate (*A*); Burma Terrane [= West Burma Block]: a separate tectonic block between the Naga Hills, Chin Hills, and Rakhine Hills mountain ranges and the Sagaing, Three Pagodas, and Ranong faults^38–40,178^; the Mogok–Mandalay–Mergui Belt^40^ is placed here as a marginal part of the Burma Terrane (*B*); Indian Plate (*C*); and Sunda Plate with the Indochina Block and Sibumasu Terrane (*D*). Second, we tested the role of former supercontinents in the origin of the Parreysiinae clades as follows: Gondwana and its fragments [African Plate + Indian Plate + Burma Terrane]^40,177^ (*A*); and Laurasia [Sunda Plate]^39^ (*B*).

Ancestral areas were reconstructed with BioGeoBEARS packages^200,201^ implemented in RASP v. 4.2^202^. As input files, we used the set of trees and the consensus phylogeny obtained from BEAST runs (see above). The branch length of the trees was converted from years to Myr using a “Scaling Branch Length” option of RASP v. 4.2^202^ with an appropriate scaling coefficient (1.0E-6). The four outgroup taxa were removed from the trees using a “Remove Selected Groups” option of RASP v. 4.2^202^. The biogeographic analyses were run with default settings (max areas = 2) but without a set of + J models checking for founder-event speciation^200^, because these models appear to be rather doubtful from a statistical and conceptual point of view^203^.

To find the most appropriate biogeographic models, we conducted a comparative analysis of the relative probability of BioGeoBEARS models (DEC, DIVALIKE, and BAYAREALIKE) using the log-likelihood (LnL), AICc, and model weight according to ΔAICc (AICc wt)^200–202^. For the “supercontinents” reconstruction, the DIVALIKE model shared higher relative probability compared with others with respect to the AICc wt value (Supplementary Table 1). In its turn, the DEC model could be chosen according to that criterion among those reconstructing ancestral areas on the basis of tectonic plates (Supplementary Table 1). However, we selected the DIVALIKE model, sharing similar LnL and AICc values to the DEC model, as the preferred model, because the DEC model did not return well-resolved reconstructions on several nodes. In both (supercontinents and tectonic plates) cases, we additionally calculated S-DIVA model with RASP v. 4.2^202^ and combined DIVALIKE and S-DIVA^204^ models using the “Combine Results” option of the software^202^. These combined models were used in subsequent biogeographic analyses and reconstructions.

### Tectonic plate modeling

The tectonic plate reconstructions for selected time intervals were calculated using GPlates 2.3 software (https://www.gplates.org)^205^ and a corresponding set of digital layers on topological plate model^206–209^. Additional settings were obtained from a set of novel tectonic and paleomagnetic reconstructions^39–42,48,137,139^. The paleogeographic positions of the Burma Terrane and Greater India at 165–170, 135, 100, 75, and 40 Myr were modified manually on the basis of our time-calibrated phylogenetic reconstruction and statistical biogeographic models (see above).

### Nomenclatural acts

The electronic edition of this article conforms to the requirements of the amended International Code of Zoological Nomenclature (ICZN), and hence the new name and combinations contained herein are available under that Code from the electronic edition of this article. This published work and the nomenclatural acts it contains have been registered in ZooBank (http://zoobank.org), the online registration system for the ICZN. The LSID for this publication is: http://zoobank.org/urn:lsid:zoobank.org:pub:FABE4C0F-313E-4AB8-803F-3523595D9A39. The electronic edition of this paper was published in a journal with an ISSN, and has been archived and is available from PubMed Central.

## Supplementary Information

Supplementary Information 1.

Supplementary Information 2.

Supplementary Information 3.

## Data Availability

The voucher specimens of freshwater mussels from the Oriental Region are available in SMF— Senckenberg Museum, Frankfurt, Germany; RMBH—Russian Museum of Biodiversity Hotspots, Federal Center for Integrated Arctic Research of the Ural Branch of the Russian Academy of Sciences, Arkhangelsk, Russia; ZSI—Zoological Survey of India, Kolkata, India; and FBRC ZSI—Freshwater Biology Regional Centre, Zoological Survey of India, Hyderabad, India. The DNA sequences generated in this study could be downloaded from NCBI’s GenBank (https://www.ncbi.nlm.nih.gov/genbank). The DNA sequence accession numbers and collecting locality data for every sample are presented in Datasets 1 and 2.
