# Supplementary material for: Oriental freshwater mussels arose in East Gondwana and arrived to Asia on the Indian Plate and Burma Terrane
Source: Sci Rep. 2022 Jan 27;12:1518. doi: 10.1038/s41598-022-05257-0 (PMC8795121; doi:10.1038/s41598-022-05257-0)
Supplement: Supplementary file 1 — Supplementary Information 1. [file 41598_2022_5257_MOESM1_ESM.pdf]

SUPPLEMENTARY INFORMATION

Oriental freshwater mussels arose in East Gondwana and arrived to Asia on the Indian Plate and Burma Terrane

Ivan N. Bolotov, Rajeev Pasupuleti, N. V. Subba Rao, Suresh Kumar Unnikrishnan, Nyein Chan, Zau Lunn, Than Win, Mikhail Y. Gofarov, Alexander V. Kondakov, Ekaterina S. Konopleva, Artyom A. Lyubas, Alena A. Tomilova, Ilya V. Vikhrev, Markus Pfenninger, Sophie S. Düwel, Barbara Feldmeyer, Hasko F. Nesemann & Karl-Otto Nagel

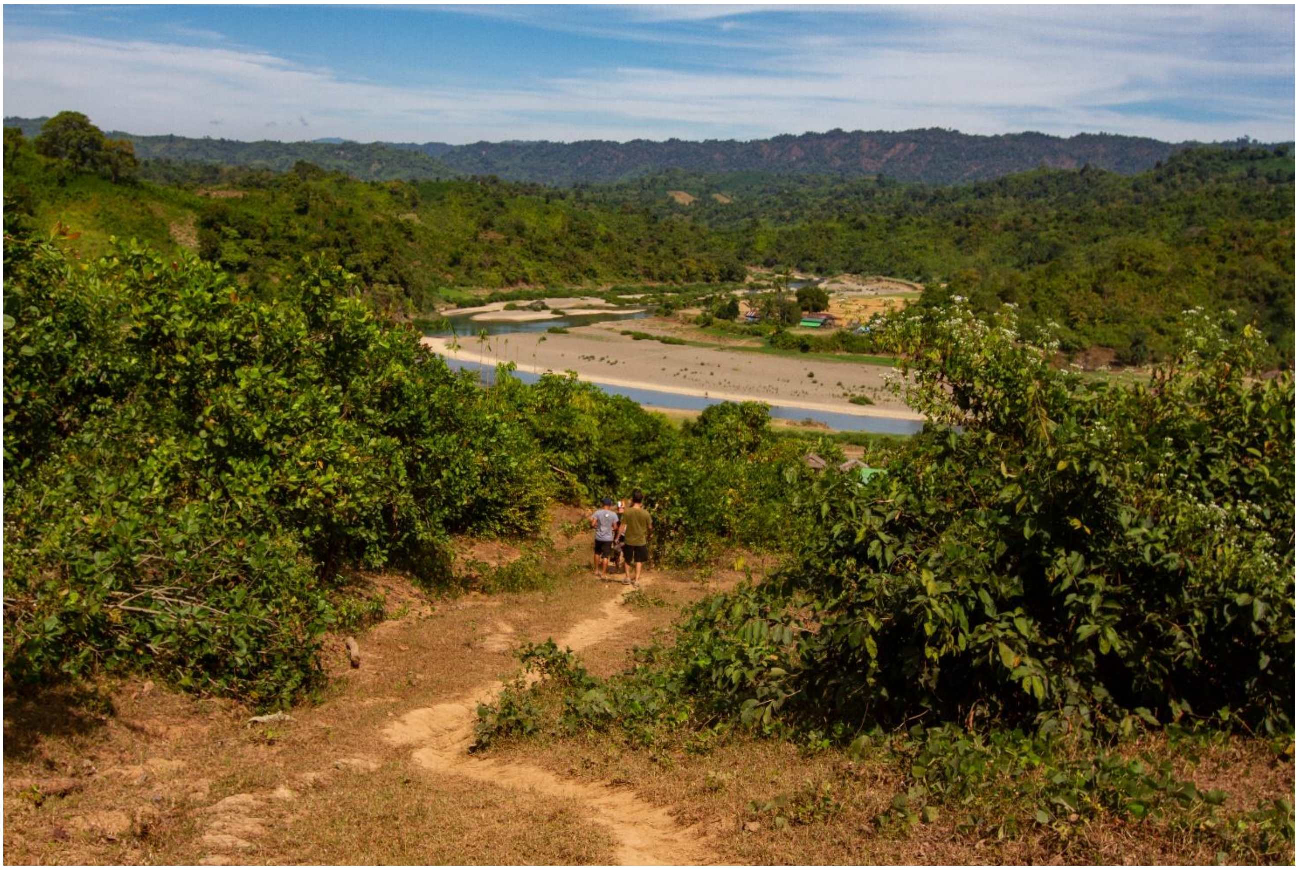

Title image: Scenic view to the Rakhine Mountains from the Toungup River valley, Western Myanmar. This 950 km-long range serves as a part of the main geographic barrier separating freshwater mussel faunas of the Indian Subcontinent and Burma Terrane. The coastal Toungup River empties into the Bay of Bengal and houses an abundant population of *Parreysia rakhinensis*, belonging to the Indian *corrugata* species group. (Photo: I. V. Vikhrev).

Contents

Supplementary Figures

Supplementary Figure 1. Maximum likelihood multi-locus phylogeny of the Parreysiinae based on the complete data set of mitochondrial and nuclear sequences (five partitions: three codons of *COI* + *16S rRNA* + *28S rRNA*).

Supplementary Figure 2. Maximum likelihood phylogeny and species delimitation of the Parreysiinae based on the *COI* haplotype alignment.

Supplementary Figure 3. Shell variability of the type series of *Parreysia keralaensis* **sp. nov.** (outer view).

Supplementary Figure 4. Shell variability of the type series of *Parreysia keralaensis* **sp. nov.** (inner view).

Supplementary Figure 5. Collecting localities of *Parreysia keralaensis* **sp. nov.** in the Periyar River, Kerala, India. (Photos: R. Pasupuleti).

Supplementary Figure 6. Living individuals of *Parreysia keralaensis* **sp. nov.** (shown by white arrows) and *Lamellidens* sp. from the downstream section of the Periyar River, Aluva, Kerala, India. (Photo: R. Pasupuleti).

Supplementary Tables

Supplementary Table 1. Relative probability of biogeographic models calculated for Oriental freshwater mussel assemblage (Unionidae: Parreysiinae) using BioGeoBEARS through RASP v. 4.2.

Supplementary Table 2. Shell measurements and reference COI sequences of the type series of *Parreysia keralaensis* **sp. nov.**

Supplementary Notes

Supplementary Note 1. Taxonomic review of recent freshwater mussels (Unionidae) from the Indian Subcontinent with supplement of congeneric species from Western Indochina (Indian Plate and Burma Terrane, respectively).

Supplementary Note 2. Taxonomic review of the Late Cretaceous Unionidae (Parreysiinae) from the Intertrappean Beds of the Deccan Plateau, Indian Plate.

Supplementary Note 3. Taxonomic review of doubtful and uncertain freshwater mussel taxa from India described by Constantine S. Rafinesque.

Supplementary References

Supplementary Datasets

Dataset 1. Information on DNA sequences of freshwater mussels (Unionidae) used in this study [separate Excel file].

Dataset 2. Information on DNA sequences of freshwater mussels (Unionidae) used to reconstruct multi-locus phylogenies (one haplotype per species) [separate Excel file].

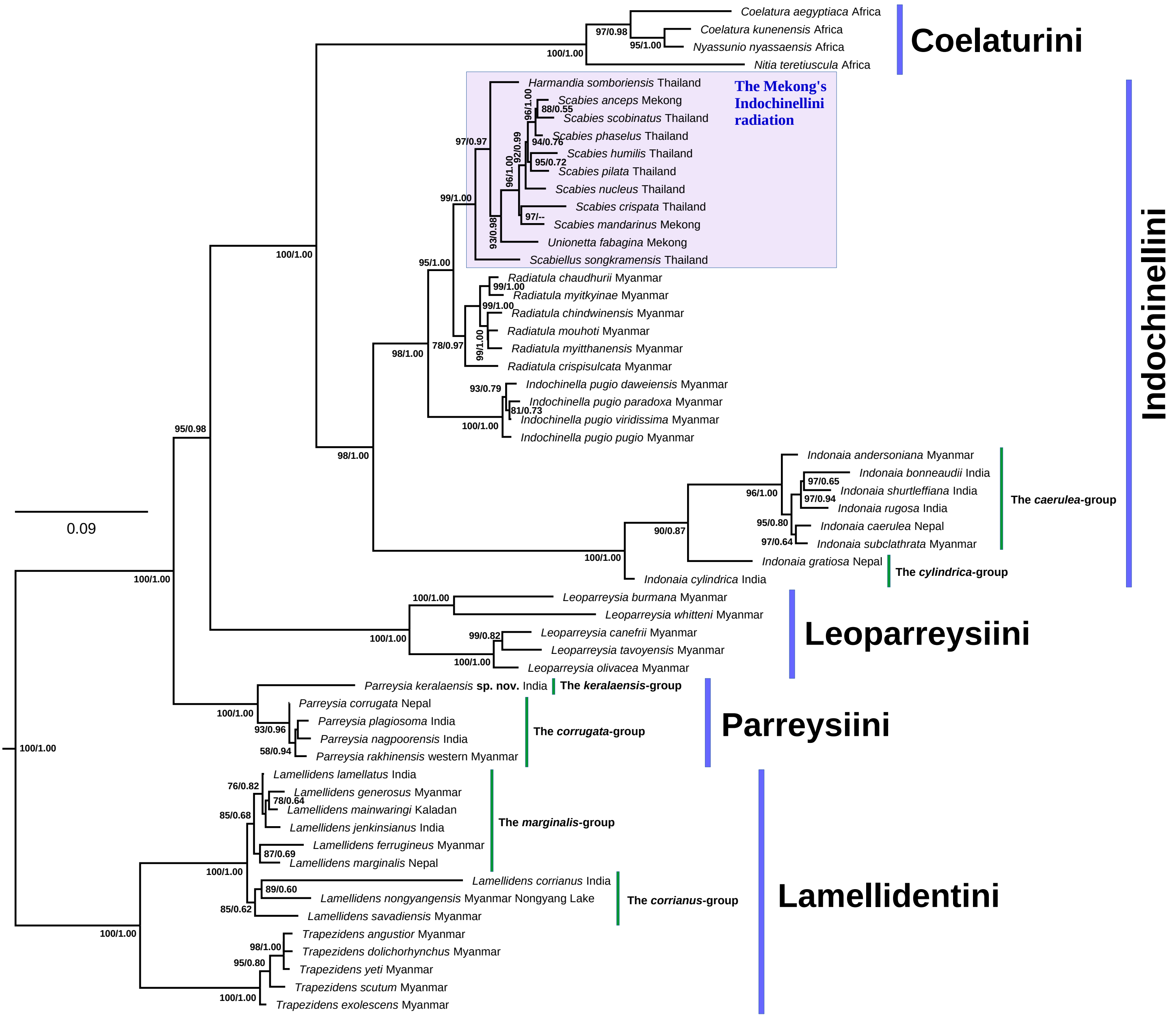

Supplementary Figure 1. Maximum likelihood multi-locus phylogeny of the Parreysiinae based on the complete data set of mitochondrial and nuclear sequences (five partitions: three codons of *COI* + *16S rRNA* + *28S rRNA*). Black numbers near nodes indicate BS values of IQ-TREE v. 1.6.12 / BPP values of MrBayes v. 3.2.7a. The outgroup taxa are not shown.

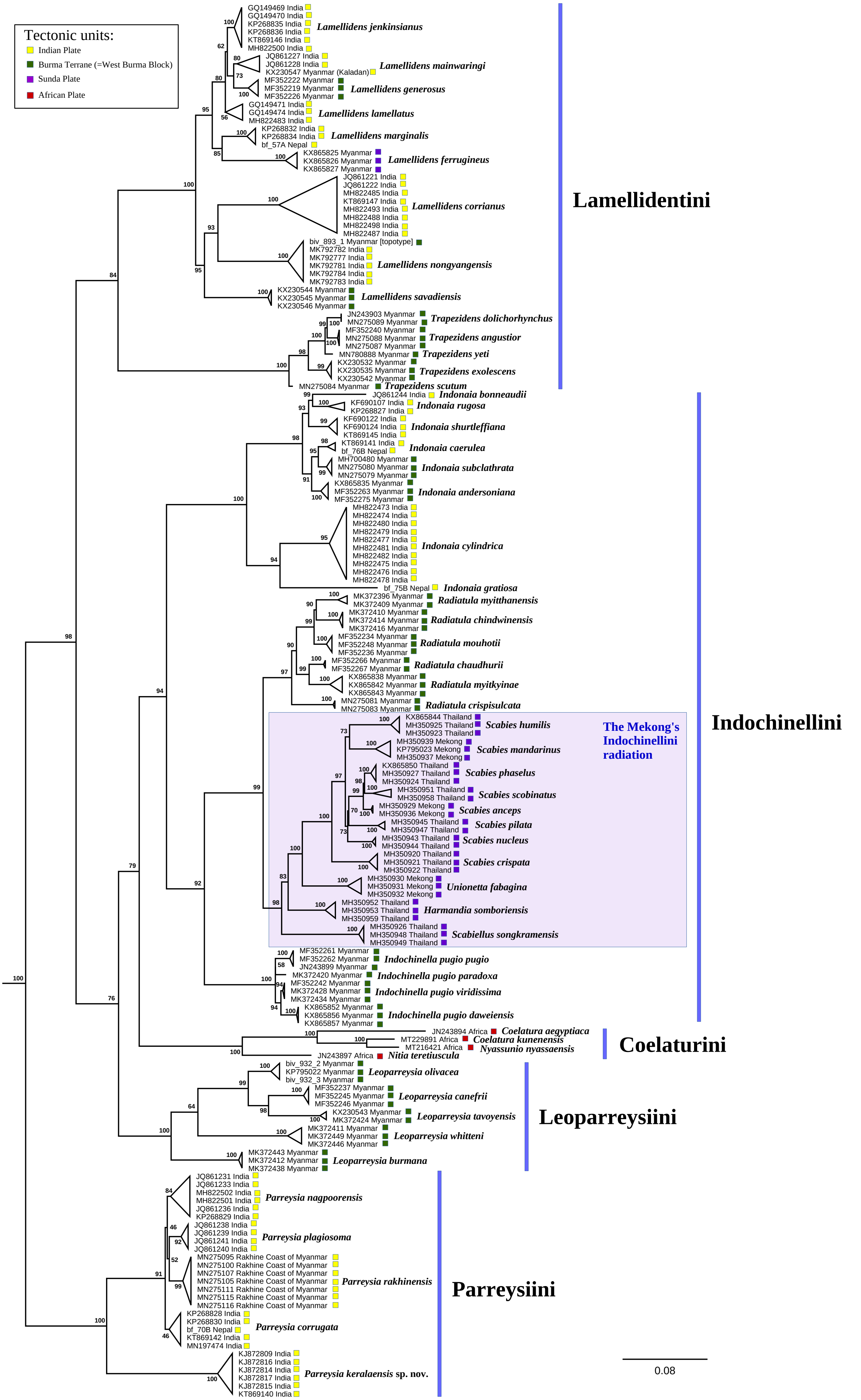

Supplementary Figure 2. Maximum likelihood phylogeny and species delimitation of the Parreysiinae based on the COI haplotype alignment. Black numbers near nodes indicate BS values of IQ-TREE v. 1.6.12. Scale bar indicates the branch length (substitutions per site). The outgroup taxa are not shown.

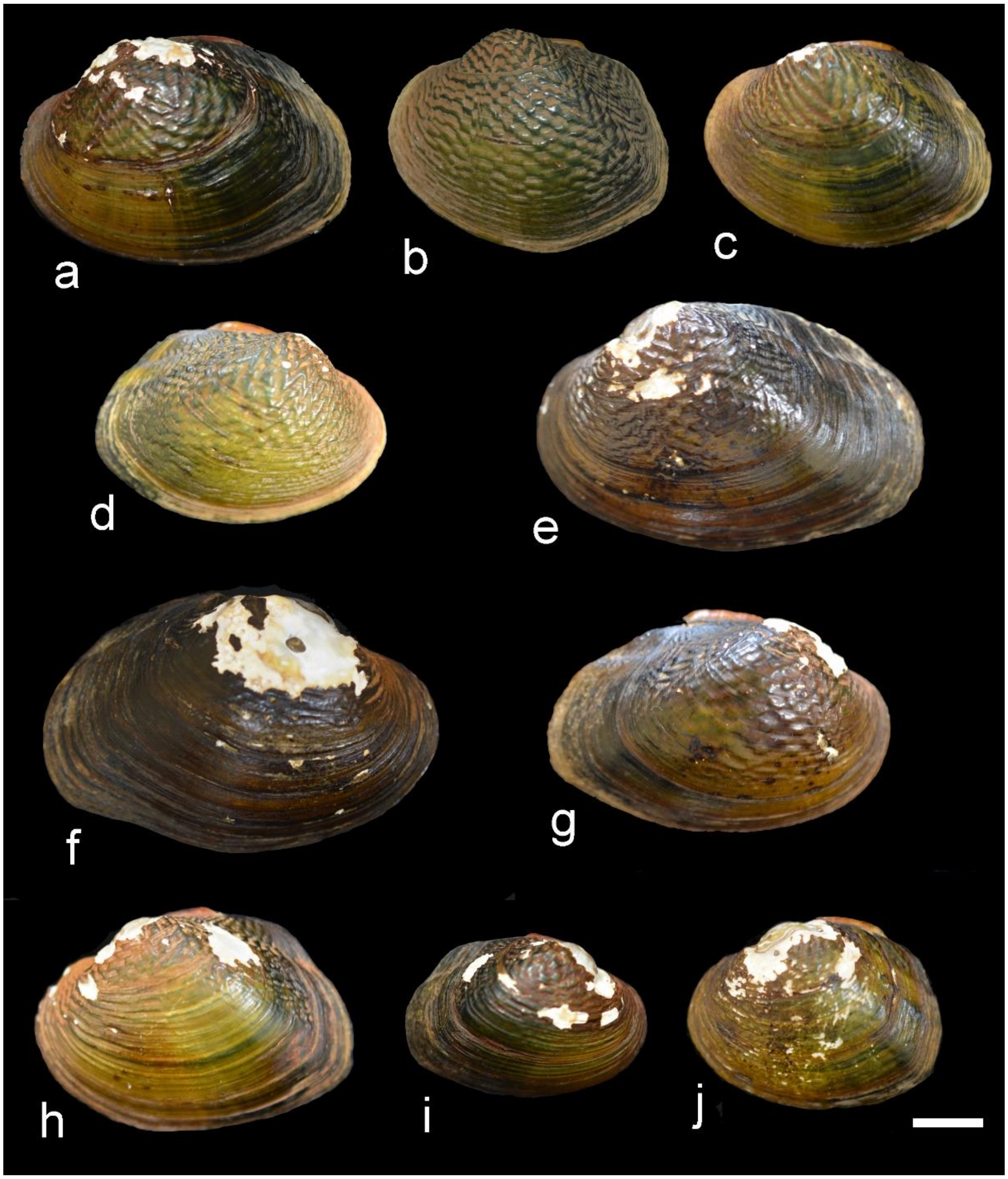

Supplementary Figure 3. Shell variability of the type series of *Parreysia keralaensis* **sp. nov.**: (**a**) holotype FBRC ZSI 1007-a (RCB2), Periyar River (downstream), Aluva, Kerala, India; (**b**) paratype FBRC ZSI 1007 (RCB3), type locality; (**c**) paratype FBRC ZSI 1007 (RCB4), type locality; (**d**) paratype FBRC ZSI 1007 (RCB5), type locality; (**e**) paratype FBRC ZSI 1007 (RCB8), type locality; (**f**) paratype FBRC ZSI 1007 (RCB9), type locality; (**g**) paratype FBRC ZSI 1007 (RCB12), type locality; (**h**) paratype FBRC ZSI 1006 (RNB1), Periyar River (upstream), Neriamangalam, Kerala, India; (**i**) paratype FBRC ZSI 1006 (RNB2), the same upstream locality; (**j**) paratype FBRC ZSI 1223 (RPC10), Achankovil River, Pampa River basin, Kizhavalloor, Kerala, India. Outer view of the left [**a**, **b**, **c**, **e**, **h**, **j**] and right [**d**, **f**, **g**, **i**] valve is shown. The type series is deposited in FBRC ZSI – Freshwater Biology Regional Centre, Zoological Survey of India, Hyderabad, India. Scale bar = 10 mm. (Photos: N. V. Subba Rao and R. Pasupuleti).

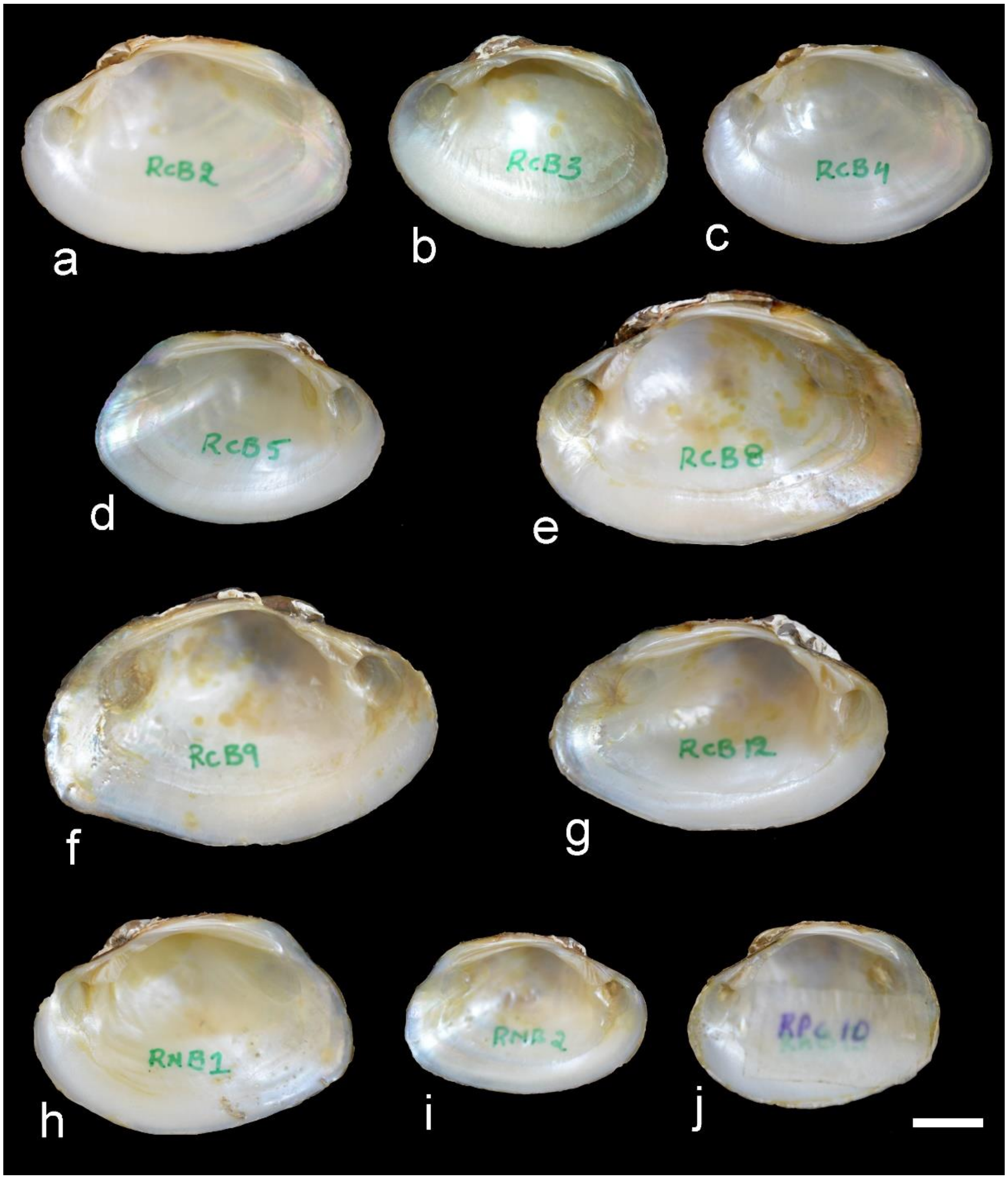

Supplementary Figure 4. Shell variability of the type series of *Parreysia keralaensis* **sp. nov.**: (**a**) holotype FBRC ZSI 1007-a (RCB2), Periyar River (downstream), Aluva, Kerala, India; (**b**) paratype FBRC ZSI 1007 (RCB3), type locality; (**c**) paratype FBRC ZSI 1007 (RCB4), type locality; (**d**) paratype FBRC ZSI 1007 (RCB5), type locality; (**e**) paratype FBRC ZSI 1007 (RCB8), type locality; (**f**) paratype FBRC ZSI 1007 (RCB9), type locality; (**g**) paratype FBRC ZSI 1007 (RCB12), type locality; (**h**) paratype FBRC ZSI 1006 (RNB1), Periyar River (upstream), Neriamangalam, Kerala, India; (**i**) paratype FBRC ZSI 1006 (RNB2), the same upstream locality; (**j**) paratype FBRC ZSI 1223 (RPC10), Achankovil River, Pampa River basin, Kizhavalloor, Kerala, India. Inner view of the right [**a**, **b**, **c**, **e**, **h**, **j**] and left [**d**, **f**, **g**, **i**] valve is shown. The type series is deposited in FBRC ZSI – Freshwater Biology Regional Centre, Zoological Survey of India, Hyderabad, India. Scale bar = 10 mm. (Photos: N. V. Subba Rao and R. Pasupuleti).

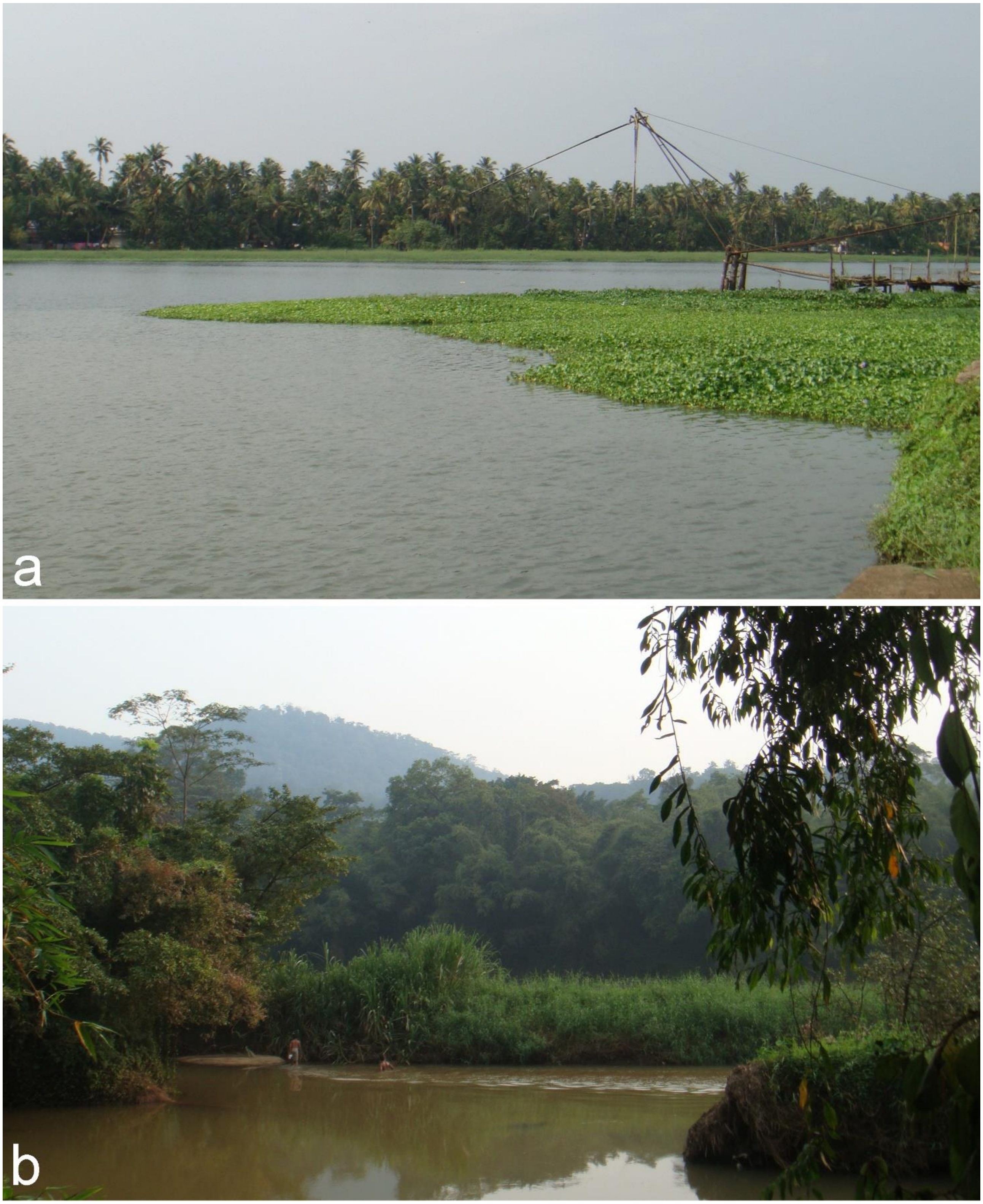

Supplementary Figure 5. Collecting localities of *Parreysia keralaensis* **sp. nov.** in the Periyar River, Kerala, India: (**a**) downstream section, 10.11°N, 76.37°E, Aluva (type locality); (**b**) upstream section, 10.06°N, 76.78°E, Neriamangalam. (Photos: R. Pasupuleti).

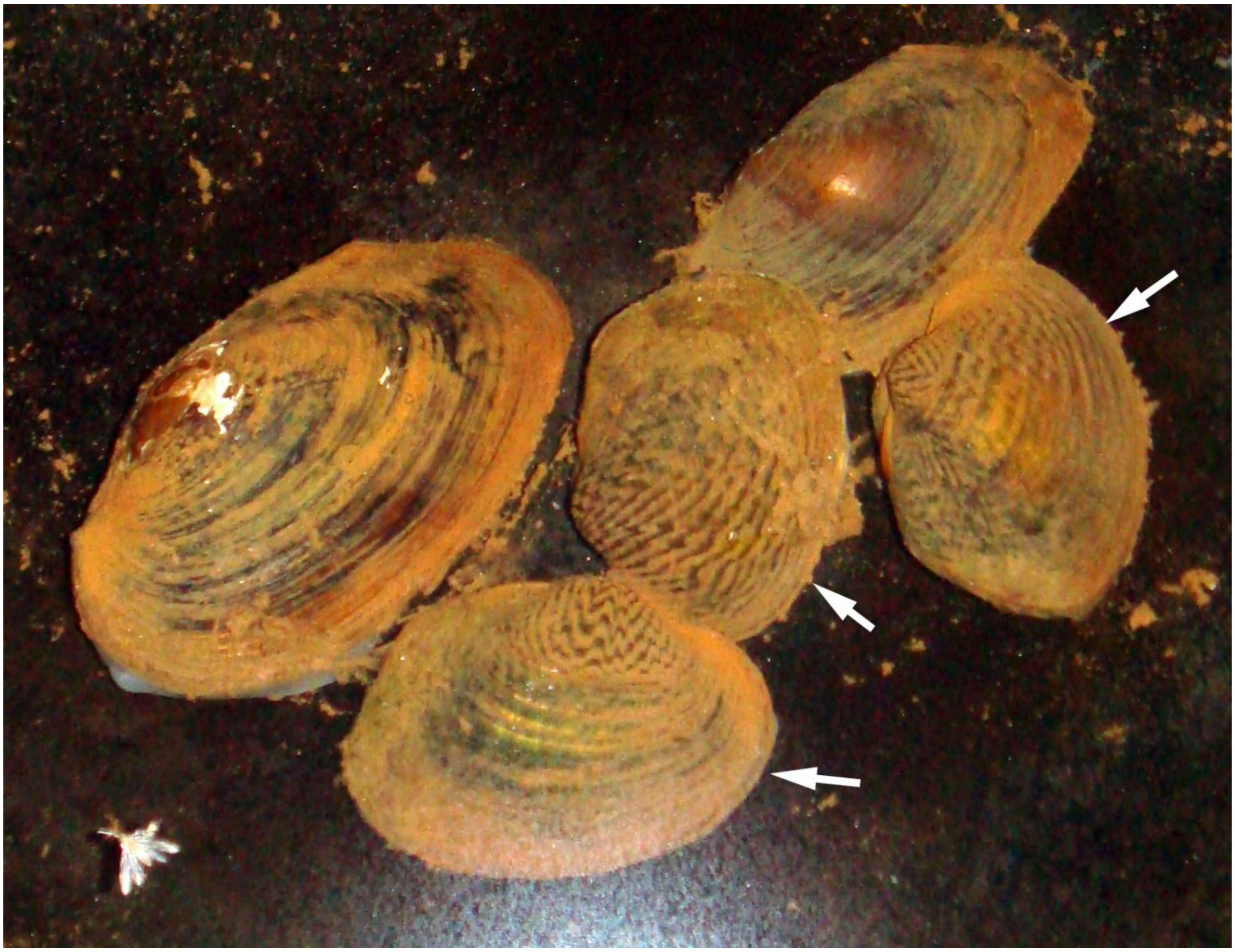

Supplementary Figure 6. Living individuals of *Parreysia keralaensis* **sp. nov.** (shown by white arrows) and *Lamellidens* sp. from the downstream section of the Periyar River, Aluva, Kerala, India. (Photo: R. Pasupuleti).

Supplementary Table 1. Relative probability of biogeographic models calculated for Oriental freshwater mussel assemblage (Unionidae: Parreysiinae) using BioGeoBEARS [1, 2] through RASP v. 4.2 [3].

**Model**	***LnL***	**Number of parameters**	***d***	***e***	**Second-order Akaike Information Criterion (*AICc*)**	**Model weight according to ΔAICc (*AICc wt*)**
Tectonic plates (four distribution areas):						
DEC*	-51.95	2	0.0012	1.00E-12	108.1	0.86
DIVALIKE	-53.79	2	0.0020	1.00E-12	111.8	0.14
BAYAREALIKE	-71.67	2	0.0016	0.009	147.6	2.30E-09
Supercontinents (two distribution areas):						
DEC	-14.69	2	0.0011	1.00E-12	33.59	0.19
DIVALIKE*	-13.21	2	0.0011	1.00E-12	30.64	0.81
BAYAREALIKE	-23.45	2	0.0012	0.0012	51.13	2.90E-05

*The most probable model based on the highest AICc wt value.

Supplementary Table 2. Shell measurements and reference *COI* sequences of the type series of *Parreysia keralaensis* **sp. nov.** The type series is deposited in FBRC ZSI – Freshwater Biology Regional Centre, Zoological Survey of India, Hyderabad, Telangana, India.

**Status of specimen**	**ZSI museum lot number (specimen code*)**	**Locality data and collector**	***COI* seq. acc. number**	**Shell measurements (mm)**		
				**SL**	**SH**	**SW**
Holotype	FBRC ZSI 1007-a (RCB2)	Aluva, Periyar River, Kerala, 17.01.2014, R. Pasupuleti leg.	KJ872811	50.0	33.5	23.8
Paratype	FBRC ZSI 1007 (RCB3)	Ditto	KJ872812	41.9	31.0	20.2
Paratype	FBRC ZSI 1007 (RCB4)	Ditto	KJ872813	43.3	30.9	19.6
Paratype	FBRC ZSI 1007 (RCB5)	Ditto	KJ872814	40.0	28.4	19.2
Paratype	FBRC ZSI 1007 (RCB8)	Ditto	KJ872815	59.1	37.6	27.1
Paratype	FBRC ZSI 1007 (RCB9)	Ditto	KJ872816	58.7	38.2	28.8
Paratype	FBRC ZSI 1007 (RCB12)	Ditto	KJ872817	48.9	29.9	26.4
Paratype	FBRC ZSI 1006 (RNB1)	Neriyamangalam, Periyar River, Kerala, 01.12.2014, R. Pasupuleti leg.	KJ872809	43.1	31.0	23.4
Paratype	FBRC ZSI 1006 (RNB2)	Ditto	KJ872810	35.9	23.7	15.1
Paratype	FBRC ZSI 1223 (RPC10)	Achankovil, Pathanamthitta, Kerala, 03.09.2014, R. Pasupuleti leg.	KT869140	34.8	24.4	18.4

*The specimen code is handwritten by a green marker on the inner side of each valve (see Supplementary Fig. 4).

Supplementary Note 1. Taxonomic review of recent freshwater mussels (Unionidae) from the Indian Subcontinent with supplement of congeneric species from Western Indochina (Indian Plate and Burma Terrane, respectively)

Family Unionidae Rafinesque, 1820

Subfamily Parreysiinae Henderson, 1935

Tribe Indochinellini Bolotov, Pfeiffer, Vikhrev & Konopleva, 2018

Genus *Indonaia* Prashad, 1918

The *caerulea*-group

Indonaia andersoniana (Nevill, 1877)

=*Unio andersoniana* Nevill (1877): p. 40 [4]. Type locality: Myadoung, Burma [Irrawaddy River near Mya Taung village, 23.7310°N, 96.1486°E, Myanmar] [4].

Type: Syntypes ZSI M5192/1, Zoological Survey of India, Kolkata, India [5] (examined by us).

Distribution: Irrawaddy to Salween Basin, Myanmar [6].

Comments: The taxonomic concept of this species was established earlier based on morphological and phylogenetic approaches [6].

Indonaia bonneaudii (Eydoux, 1838) **comb. rev.**

=*Unio bonneaudii* Eydoux (1838): p. 190, pl. 60, figs. 1-1a [7]. Type locality: Les rivières de la presqu'ile de l'Inde [rivers of the Indian Peninsula] [7].

=*Unio leioma* Benson (1862): p. 192 [8]. Type locality: regione Dekhan (?), prope Bombay [Deccan Plateau (?) near Mumbai, Maharashtra, India] [8].

Type: Not traced.

Distribution: Karli River, Western Ghats, India.

Comments: It was thought that this species belongs to the genus *Radiatula* [9] and is widespread from India to Myanmar [10]. Multiple authors listed its historical records [11, 12, 13, 14, 15] and modern samples [16, 17] from the Western Indochina Subregion (Myanmar). However, our novel results reveal that this species is endemic to western India and that it clusters with the *Indonaia* clade. All the occurrences of *Indonaia bonneaudii* from Myanmar [10] should be reconsidered as belonging to other taxa. Our concept of this taxon is close to that of Haas [18], who considered it as a rare and little-known species.

Indonaia caerulea (Lea, 1831)

=*Unio caeruleus* Lea (1831): p. 95 (105), pl. 13, fig. 25 [19]. Type locality: Bengal, India [based on the holotype's label], while in the protologue it is listed as follows: "River Hoogly, Hindostan" [Hooghly River, Ganges Basin, India] [19].

=*Lampsilis argyratus* Rafinesque (1831): p. 7 [20]. Type locality: River Ganges [20].

=*Unio gaudichaudii* Eydoux (1838): p. 190, pl. 59, fig. 3 [7]. Type locality: Les petites rivières du Bengale [small rivers of Bengal, India] [7].

=*Unio keraudreni* Eydoux (1838): p. 188, pl. 59, figs. 1-1a [7]. Type locality: Les petites rivières et les mares du Bengale [small rivers and ponds of Bengal, India] [7].

=*Unio gerbidoni* Eydoux (1838): p. 189, pl. 59, figs. 2-2b [7]. Type locality: Les petites rivières et les eaux douces stagnantes de la côte de Coromandel [small rivers and stagnant fresh waters of the Coromandel Coast, India] [7].

=*Unio nuttallianus* Lea (1856): p. 103 [21]. Type locality: India [21].

=*Unio pachysoma* Benson (1862): p. 186 [8]. Type locality: Assam [8].

=*Unio evitatus* Lea (1866): p. 133 [22]. Type locality: Bengal [22].

=*Trapezoideus dhanushori* Annandale & Prashad (1921): p. 611, figs. 30, 31a, b [12]. Type locality: Dhanushori Stream at a distance of about a mile from Dimapur, Assam [Dhanushori Stream, approx. 26.9260°N, 93.7523°E, Brahmaputra Basin, Assam, India] [12].

Type: Holotype NMNH 85193, National Museum of Natural History, Smithsonian Institution, Washington, DC, USA (examined by us).

Distribution: Ganges Basin in India, Nepal, and Bhutan, Brahmaputra and Krishna basins in India; Surma River in Bangladesh (shell lot UMMZ 109604), Indus Basin in Pakistan [15, 23, 24, 25, 26].

Comments: It is a conchologically variable species, some morphological varieties of which were described as separate nominal taxa. Here, we largely follow the traditional view on this species [15, 18, 27, 28] and use *Indonaia caerulea* as its oldest available name but provide an updated synonymy for this taxon. Our examination of the *Trapezoideus dhanushori* holotype (ZSI M11962/2; by original designation) revealed that it conchologically corresponds to *Indonaia caerulea*. The holotype of *Unio caeruleus* shares a thinner shell and lamellar pseudocardinal teeth, as does our sequenced sample from India. Conversely, our shell lots from Nepal deposited in SMF share an extremely high degree of variability, i.e. from a form having a rather thin, narrow shell, weakly developed umbonal sculpture, and elongated pseudocardinal teeth to a variety with ovate shell, strongly developed umbonal sculpture, and short, massive pseudocardinal teeth. The latter variety (see Fig. 4c) externally looks similar to *Indonaia gratiosa* **comb. nov.** Haas [18] believed that this conchological variability reflects environmental conditions, leading to the origin of river, stream, and lake forms.

Indonaia rugosa (Gmelin, 1791) **comb. nov.**

=*Mya rugosa* Gmelin (1791): p. 3222, sp. 32 [29]. Type locality: Coromandel fluviis [rivers of the Coromandel Coast of India] [29].

=*Diplasma striata* Rafinesque (1831): p. 6 [20]. Type locality: River Jellinghy in Bengal [Jalangi River, approx. 23.4356°N, 88.4905°E, Ganges Basin, West Bengal, India] [20].

=*Unio radula* Hanley (1856): p. 382, pl. 23, fig. 41 [unavailable; primary homonym of *Unio radula* Say, 1829] [30]. Type locality: Assam [30].

=*Unio scobina* Hanley (1856): p. 382, pl. 23, fig. 40 [30]. Type locality: Assam [30].

=*Nodularia* (*Radiatula*) *lima* Simpson (1900): p. 820 [replacement name for *Unio radula* Hanley] [31].

Type: Not traced.

Distribution: Ganges, Brahmaputra, and Krishna basins, India. There is a recent record from Sisi River [27.325°N, 94.839°E], a tributary of Dihing River, Upper Brahmaputra Basin, Assam [erroneously identified as *Scabies crispata* (Gould, 1843)] (Ref. [32]: p. 18244, Fig. 2.7).

Comments: This species shares a rather thin, ovate shell having a prominent sculpture with zigzag ridges, usually covering the entire shell surface. The characteristic W-shaped sculpture just below the umbo could be considered a diagnostic feature of this taxon. We propose a few new synonyms for this species based chiefly on morphological features.

Indonaia shurtleffiana (Lea, 1856)

=*Unio shurtleffianus* Lea (1856): p. 94 [21]. Type locality: Sina River, <...> Ahmednugger, India [upper reaches of the Sina River near Ahmednager, approx. 19.0835°N, 74.7281°E, Krishna Basin, Maharashtra, India] [21].

=*Indonaia khadakvaslaensis* Ray (1966): p. 226, figs. 1a-d [33]. Type locality: Khadakvasla Dam, Pune [Khadakwasla Reservoir, approx. 18.4383°N, 73.7654°E, Krishna Basin, Maharashtra, India] [33].

Type: Syntype MCZ 175566, Museum of Comparative Zoology, Cambridge, USA (examined by us).

Distribution: Krishna and Godavari basins, India.

Comments: This species shares an ovate or somewhat elongated shell, usually more or less tapering posteriorly, which is rather similar in the general outline to that of *Nodularia* taxa. The umbo and anterior margin usually covered by a fine wave-like sculpture, while the posterior part of the shell bears fine striations. *Indonaia khadakvaslaensis* is considered here a junior subjective synonym of *I. shurtleffiana* based on morphological features and close proximity of their type localities, belonging to the Upper Krishna Drainage [21, 33].

Indonaia subclathrata (Martens, 1899)

=*Unio misellus* var. *subclathratus* Martens (1899): p. 44, pl. 6, fig. 3 [34]. Type locality: Chindwinfluss bei Kalewa und bei Matu < … >; einige Stücke auch im Irawaddi selbst bei Yenangyoung [Chindwin River near Kalewa and Matu, approx. 23.1991°N, 94.3071°E, several specimens also from Irrawaddy River near Yenangyaung, approx. 20.4347°N, 94.8720°E, Myanmar] [34].

Type: Not traced (it was not found in the Museum für Naturkunde [ZMB], Berlin, Germany).

Distribution: Lower Manipur River and a corresponding section of the Chindwin River, Irrawaddy Basin, Myanmar [6, 35].

Comments: The taxonomic concept of this species was established earlier based on morphological and phylogenetic approaches [6, 35].

Indonaia theobaldi (Preston, 1912)

=*Nodularia* (s. str.) *theobaldi* Preston (1912): p. 292 [36]. Type locality: Manipur, Assam [36].

Type: Holotype ZSI 4710/1, Zoological Survey of India, Kolkata, India [5] (examined by us). Paratype SMF 3634, Senckenberg Museum, Frankfurt, Germany (examined by us).

Distribution: Upper Manipur Valley (including Logtak Lake), India [12].

Comments: It seems to have a restricted distribution in the Upper Manipur Basin [12]. The pseudocardinal teeth of this species are similar to those in *Radiatula* taxa. The DNA sequences of

this species are lacking, and its taxonomic status needs to be confirmed in the future based on a sample of sequenced topotypes.

The *cylindrica*-group

Indonaia cylindrica (Annandale & Prashad, 1919) **comb. nov.**

=*Parreysia cylindrica* Annandale & Prashad (1919b): p. 150, pl. 4, figs. 6-7 [37]. Type locality: Yenna River, Upper Kistna watershed, at Medha [Venna River at Medha (now Kanher Reservoir), 17.7887°N, 73.8254°E, Krishna Basin, Maharashtra, India] [37].

Type: Syntypes ZSI M11398/2, Zoological Survey of India, Kolkata, India [5] (examined by us).

Distribution: Endemic to the Upper Krishna Basin, India [37].

Comments: This species was described as a *Parreysia* member based on its massive, sculptured shell. However, our phylogeny indicates that it should be placed within *Indonaia*.

Indonaia gratiosa (Philippi, 1843) **comb. nov.**

=*Unio gratiosus* Philippi (1843): p. 20, pl. 1.6 *Unio* Tab. I, fig. 5 [38]. Type locality: Nova Hollandia [erroneous: somewhere in India] [38].

=*Unio corbis* Hanley (1856): p. 386, pl. 23, fig. 43 [30]. Type locality: Assam [30].

=*Unio occatus* Lea (1860): p. 307 [39]. Type locality: Bengal, India [39].

=*Unio macilentus* Benson (1862): p. 187 [8]. Type locality: rivulo Choia Nuddy dicto, non procul a Bijnore, provinciae Rohilkhund [Chhoeya Stream near Bijnor, 29.3689°N, 78.1794°E, Ganges Basin, Uttar Pradesh, India] [8].

=*Unio siliguriensis* Preston (1908): p. 47, fig. 7 [40]. Type locality: Siliguri, N. Bengal [Siliguri, Ganges Basin, West Bengal, India] [40].

Type: Not traced.

Distribution: Ganges and Brahmaputra basins, India and Nepal.

Comments: This species shares an ovate shell with rather prominent radial ridges, covering the umbo region or the entire shell surface. Here, we propose an updated synonymy of this species based on morphological features with supplement of the available DNA sequence data.

The *involuta*-group

Indonaia involuta (Hanley, 1856)

=*Unio involutus* Hanley (1856): p. 385, pl. 23, fig. 19 [30]. Type locality: Assam [30].

Type: Holotype NHMUK 1968656, Natural History Museum, London, United Kingdom (examined by us).

Distribution: Upper Brahmaputra Basin in India (the holotype) and Surma River in Bangladesh (shell lot UMMZ 109741). This species is known from only a few historical records. It was not re-discovered during a modern survey of freshwater molluscs in Assam [32].

Comments: It is a thin-shelled freshwater mussel, largely resembling members of the genus *Lamellidens* in the general shell outline and low shell thickness. The pseudocardinal teeth are

lamella-like, strongly reduced. Its taxonomic position needs further research efforts, as the DNA sequences of this unusual species are not available.

Indonaia olivaria (Lea, 1831)

=*Unio olivarius* Lea (1831): p. 108, pl. 16, fig. 38 [19]. Type locality: Bengal [based on the syntypes' label], while in the protologue it is listed as follows: Burrill River, India [19]. Blanford [41] noted that "I do not know if where be such a river as Burrill, but the locality for the original type is very probably the neighbourhood of the Burrail Range, north of Cachar, as the shell was received by Lea from Dr. Burrough who collected extensively in Assam...". However, most available museum lots of this species were collected from three localities in the Ganges Basin: Ramganga River near Moradabad, Uttar Pradesh (NHMUK MP D308, NHMUK MP D066, NHMUK MP D067, NHMUK MP D090, and NHMUK 1954-6-2-1459), Yamuna River near Agra, Uttar Pradesh (SMF 13532 and NHMUK 90-6-3-4-5), and Jalangi River, West Bengal (NMNH 85200 and ANSP 68485). It was not found during a modern survey of freshwater molluscs in Assam [32]. Hence, Bengal appears to be the correct type locality of this species.

Type: Syntypes ANSP 41742, Academy of Natural Sciences, Philadelphia, USA (not examined by us).

Distribution: Ganges Basin, India [24].

Comments: This species shares a thin, rather fragile shell, narrow hinge plate, and lamella-like pseudocardinal teeth, as does *Indonaia involuta*. Its taxonomic affinities needs further clarification based on the DNA sequences of a topotype sample, which is yet to be collected.

Tribe Lamellidentini Modell, 1942

Genus *Arcidopsis* Simpson, 1900

Arcidopsis footei (Theobald, 1876)

=*Unio footei* Theobald (1876): p. 187, pl. 14, fig. 9 [42]. Type locality: Kistna flumine prope 'Gutparba Falls' [Gokak Falls, Ghataprabha River, 16.1929°N, 74.7827°E, Upper Krishna Basin, Karnataka, southwestern India] [35, 42].

=*Trapezoideus prashadi* Haas (1922): p. 101 [43]. Type locality: Mysore, Südostindien [the former State of Mysore, Upper Krishna Basin, Karnataka, southwestern India] [35, 43].

Type: Holotype NHMUK 88-12-4-1651, Natural History Museum, London, United Kingdom (examined by us).

Distribution: Upper part of the Krishna basin in Western Ghats, India [44, 45, 46]. At first glance, historical records from Mysore [43, 44] could be linked to the Upper Kaveri Basin near the city of Mysuru [35] but are likely referred to the former State of Mysore, which also covered part of the Upper Krishna Basin.

Comments: The taxonomic concept of *Arcidopsis footei* was updated earlier, when *Trapezoideus prashadi* was found to be a junior synonym of this species [35]. Graf & Cummings [9] placed *Arcidopsis* in the tribe Lamellidentini based on conchological features. The DNA sequences of this peculiar taxon are not available.

Genus *Lamellidens* Simpson, 1900

The *corrianus*-group

Lamellidens corrianus (Lea, 1834)

=*Unio corrianus* Lea (1834): p. 65, pl. 9, fig. 25 [47]. Type locality: Calcutta, India [Kolkata, Ganges Basin, India] [47].

=*Unio theca* Benson (1862): p. 186 [8]. Type locality: Fluvio Cane, prope Banda, Bundelkhund [Ken River near Banda, approx. 25.4836°N, 80.3128°E, Ganges Basin, Uttar Pradesh, India] [8].

Type: Holotype NMNH 86056, National Museum of Natural History, Smithsonian Institution, Washington, DC, USA (examined by us).

Distribution: Ganges and Krishna basins, India.

Comments: It is a rather well defined species, having a more or less elongated shell. By the general shell outline, it largely resembles several members of the genus *Trapezidens* Bolotov, Vikhrev & Konopleva, 2017 from Western Indochina. Therefore, most *Trapezidens* taxa were previously considered synonyms of *Lamellidens corrianus* [10] but our phylogenetic research revealed that the earlier taxonomy was biased by a convergent external similarity of the phylogenetically distant lineages [48, 49, 50]. Here, the nominal taxon *Lamellidens theca* is tentatively considered a junior synonym of *L. corrianus* based on conchological features that were mentioned in the original description [8].

Lamellidens nongyangensis Preston, 1912 **stat. rev.**

=*Lamellidens nongyangensis* Preston (1912): p. 306 [36]. Type locality: Nongyang Lake, South of Patkai [Lake of No Return, 27.2192°N, 96.1439°E, Irrawaddy Basin, Myanmar] [36].

=*Lamellidens narainporensis* Preston (1912): p. 305 [36]. Type locality: Narainpore Bhil, Murshidabad District, Bengal [Narayanpur village, 24.2738°N, 88.2846°E, Ganges Basin, Murshidabad District, West Bengal, India] [36].

Type: Holotype ZSI M5068/1, Zoological Survey of India, Kolkata, India [5] (examined by us).

Distribution: Ganges Basin in India, with an isolated population in Lake of No Return (Nongyang Lake), Irrawaddy Basin, Myanmar.

Comments: Earlier, we placed *Lamellidens nongyangensis* to the synonymy of *L. savadiensis* based on morphological examination of the type specimens [48]. However, the DNA sequences generated from a new topotype sample from Lake of No Return revealed that it is a separate species, which also occurs in the Ganges Drainage. Its Gangetic population was described as a separate species, *Lamellidens narainporensis*. Our first reviser action on the precedence of simultaneous synonyms: *Lamellidens nongyangensis* over *L. narainporensis* **syn. nov.**

Lamellidens savadiensis (Nevill, 1877)

=*Unio marginalis var. savadiensis* Nevill (1877): p. 37 [4]. Type locality: at Sawady in the Thengleng Stream [Sawadi village, 24.1510°N, 97.1502°E, Myanmar], also at Bhamo [Irrawaddy River near Bhamo city, 24.2594°N, 97.2202°E, Myanmar] and at Shuaygoomyo [Irrawaddy River near Shwegu town, 24.2291°N, 96.7910°E, Myanmar]; four young specimens found at Myadoung [Irrawaddy River near Mya Taung village, 23.7310°N, 96.1486°E, Myanmar] probably also belong to this form [4].

=*Lamellidens marginalis* var. *sawaddyensis* Preston (1912): p. 305 [36]. Type locality: Sawaddy River [24.1510°N, 97.1502°E, a tributary of the Irrawaddy River near Sawadi village, Myanmar] [36].

=*Lamellidens indawgyiensis* Prashad (1930): p. 253, fig. 4, pl. 8, figs. 9-10 [14]. Type locality: Indawgyi Lake [approx. 25.1209°N, 96.3300°E, Irrawaddy Basin, Myanmar] [14].

Type: Not traced.

Distribution: Middle Irrawaddy (including Lake Indawgyi) and Sittaung basins, Myanmar [48, 51].

Comments: It is a conchologically variable species with a rather restricted range. Our concept of this taxon is based on morphological data and a set of DNA sequences generated from a new topotype sample. The synonymy, presented above, is also confirmed by means of a DNA-based approach. A more thorough synonymy of this species, which will include several Tapparone Canefri's nominal taxa [11], is under development and will be published elsewhere.

Lamellidens unioides Nesemann & Sharma in Nesemann et al., 2007

=*Lamellidens unioides* Nesemann & Sharma in Nesemann et al. (2007): p. 30, pl. 12-A, figs. 1-4 [24]. Type locality: Mamu Bhanja Pokhra at Hajipur, Muzaffarpur District, Bihar, India [pond, 25.6758°N, 85.2250°E, Mamu Bhanja, Hajipur, Ganges Basin, Muzaffarpur District, Bihar, India] [24].

Type: Holotype NHMW 104161, NHMW Mollusca Collection, Naturhistorisches Museum Wien, Austria (examined by us).

Distribution: Ganges Basin, India [24].

Comments: The validity and taxonomic position of this nominal species are unclear due to the lack of DNA sequence data. However, it shares reduced pseudocardinal teeth and seems to be a separate and well-distinguishable species. Here, we place it within the *corrianus*-group based on morphological features such as a narrow, elongated shell.

The *marginalis*-group

Lamellidens candaharicus (Hutton, 1849)

=*Unio candaharicus* Hutton (1849): p. 660 [52]. Type locality: canals at Candahar [Kandahar, 31.6148°N, 65.7198°E, Sistan/Helmand Basin, Afghanistan] [52].

=*Lamellidens marginalis rhadinaeus* Annandale & Prashad (1919a): p. 59, pl. 3, figs. 9-10; pl. 7, figs. 7-11 [53]. Type locality: Helmand near Nasratabad, Seistan [Helmand River near Zabol, 30.9877°N, 61.4542°E, Sistan/Helmand Basin, Sistan and Baluchestan Province, Iran] [53].

Type: Not traced.

Distribution: Endorheic Sistan/Helmand Basin, eastern Iran and Afghanistan. Records from the Indus Basin [23], though quite possible, need further confirmation on the basis of a molecular approach.

Comments: It is the most western species in this genus, the identity of which needs further research efforts, because its DNA sequences are yet to be generated. Here, we consider *Lamellidens marginalis rhadinaeus* as a junior synonym of *L. candaharicus* based on morphological similarity and biogeographic data.

Lamellidens ferrugineus (Annandale, 1918) **stat. rev.**

=*Physunio ferrugineus* Annandale (1918): p. 139, pl. 19, figs. 4-9 [54]. Type locality: The semi-liquid mud at the bottom of the central region of the Inle Lake in water from 7 to 12 feet deep [central part of Lake Inle, 20.5903°N, 96.9025°E, Salween Basin, Myanmar] [54].

=*Physunio micropteroides* Annandale (1918): p. 139, pl. 19, figs. 1-3 [54]. Type locality: Sluggish streams on the Yawnghwe Plain, in dense mud in about 3 feet of water [tributaries of Lake Inle near Nyaungshwe, 20.6607°N, 96.9250°E, Salween Basin, Myanmar] [54].

Type: The holotype of *Physunio ferrugineus* (shell lot ZSI M11290/2) is not listed in Ramakrishna et al.'s type specimen catalogue of the Mollusca section in the Zoological Survey of India, Kolkata [5]. Our attempts to locate this shell in the ZSI collection have failed, and it is probably lost. The holotype of *Physunio micropteroides* (shell lot ZSI M11048/2) is still available in ZSI (examined by us).

Distribution: Lake Inle and streams around, Salween Basin, Myanmar.

Comments: In our earlier work on freshwater mussels from Myanmar [48], we showed that both nominal taxa from Lake Inle (*Physunio ferrugineus* and *P. micropteroides*) are conspecific and that this variable species belongs to the genus *Lamellidens*. These results were based on sequenced topotypes of both nominal taxa. The rounded shell shape of this species clearly resembles that of *Physunio* taxa such as *P. superbus* (Lea, 1843) but this pattern does not reflect a common ancestry but is attributable to convergent evolution. Initially, we assumed that *Lamellidens generosus* should be the oldest available name for this species, as it was also described from the Salween Basin [48]. However, the DNA sequences generated from topotypes of *Lamellidens generosus* from the Hlaingbwe River do not support this hypothesis. Now we found that *Lamellidens generosus* is widespread in Western Indochina and crosses a number of drainage divides from the Irrawaddy to the Lower Salween (see below). In its turn, the Inle Lake and its tributaries are inhabited by a separate *Lamellidens* species, having a rather restricted range. Our first reviser action on the precedence of simultaneous synonyms: *Lamellidens ferrugineus* over *L. micropteroides* **syn. nov.**

Lamellidens friersoni (Simpson, 1914) **comb. nov.**

=*Unio velaris* Sowerby (1868): pl. 72, sp. 368 [55]. Type locality: Assam [55].

=*Physunio friersoni* Simpson (1914): p. 1068 [introduced as a new name for *Unio velaris* Sowerby, 1868] [56].

Type: ZSI (shell lot No.?), Zoological Survey of India, Kolkata, India (examined by us).

Distribution: Upper Brahmaputra Basin, Assam, India. There is a possible recent occurrence from Diharang River [27.381°N, 95.101°E], a tributary of Dihing River, Upper Brahmaputra Basin, Assam [erroneously identified as *Trapezidens exolescens* (Gould, 1843)] (Ref. [32]: p. 18244, Fig. 2.9).

Comments: Sowerby [55] introduced this Indian species with reference to the nominal taxon *Unio velaris* Hanley, 1856. The latter taxon was stated to be collected from "Bugis, Celebes" [most likely somewhere in South Sulawesi, Indonesia] [30]. If this type locality is correct, it is indeed a remarkable occurrence, because the native Unionidae are unknown from Wallacea [9, 57], although these remote tropical islands are actively colonized by at least one invasive species [58]. Currently, Hanley's nominal taxon is placed within the synonymy of *Physunio superbus* (Lea, 1843) (Gonideinae: Contradentini). Simpson [56] introduced *Physunio friersoni* as a new name for *Unio velaris* Sowerby and provided a brief description of this taxon. Haas [59, 60] placed it in the monotypic subgenus (section) *Velunio* Haas, 1919. Later, Prashad [61] assumed that the nominal taxon of Hanley is based on the same shell from Assam in the Benson's collection (ZSI) and that its type locality on Sulawesi was given in error. Conversely, Prashad [61] did not provide any evidence supporting his hypothesis, and, hence, this assumption could not be accepted. Furthermore, the shell from Assam in ZSI [15, 28] shares a small, tubercle-like umbo with some concentric sculpture corresponding to the figure of

Sowerby (Ref. [55]: pl. 72, sp. 368). In its turn, Hanley (Ref. [30]: pl. 23, fig. 42) illustrated a shell having a broad, prominent umbo without any sculpture, which is similar to that in *Physunio superbus* and related taxa from the Sundaland Subregion. Finally, several recent researchers accepted *Physunio* (*Velunio*) *velaris* (Sowerby) as a possible combination for the Assamese species [15, 28], while others listed the correct name *Physunio friersoni* [9, 57].

As Contradentini occurrences in India are hardly expected, this particular case seems to resemble that with *Physunio ferrugineus* and *P. micropteroides* (see taxonomic account of *Lamellidens ferrugineus* for detail). These nominal taxa from Lake Inle in Myanmar were initially placed in the genus *Physunio* based on a rounded shell shape and well-developed wing [54] but later were found to represent a single species belonging to the genus *Lamellidens* [48]. Here, we transfer the nominal taxon *Physunio friersoni* Simpson, 1914 to *Lamellidens* and propose *L. friersoni* (Simpson, 1914) **comb. nov.** based on morphological and biogeographic data. Hence, *Velunio* Haas, 1919 **syn. nov.**, a monotypic subgenus (section) of the genus *Physunio* Simpson, 1900 that was established for this taxon, becomes a synonym of *Lamellidens*.

Lamellidens generosus (Gould, 1847)

=*Unio generosus* Gould (1847): p. 220 [62]. Type locality: Newville, Tavoy, British Burmah [Hlaingbwe River near the former Newville village, 16.9834°N, 97.9043°E, Myanmar] [62].

=*Unio consobrinus* Lea (1860): p. 331 [39]. Type locality: China [the type was likely collected somewhere within the Chinese part of the Salween or Irrawaddy basins in southwestern Yunnan, China] [39].

=*Lamellidens brandti* Bolotov, Konopleva & Vikhrev (2017b): p. 7 [48]. Type locality: Pathi River, Sittaung Basin, Myanmar [48].

Type: Holotype MCZ 169449, Augustus A. Gould Type Collection, Museum of Comparative Zoology, Cambridge, USA (examined by us).

Distribution: Irrawaddy to Lower Salween Basin (including Haungthayaw, Hlaingbwe, and Ataran) in Myanmar; southwestern Yunnan in China.

Comments: An updated synonymy of this variable and widespread species is under development, and will be published elsewhere. In this study, we propose two new synonyms for *Lamellidens generosus*. First, *Lamellidens brandti* **syn. nov.** was described as a new species from the Sittaung Basin based on DNA sequence data and morphological features [48]. Now we found that this nominal taxon phylogenetically clusters with new topotypes of *Lamellidens generosus*. Second, *Unio consobrinus* **syn. nov.** was thought to be a widespread species, ranging from India to Sri Lanka and Nepal [10, 15, 28, 63]. However, this nominal taxon was described from China, and here we place it in the synonymy of *Lamellidens generosus* based on morphological and biogeographic evidence.

Lamellidens jenkinsianus (Benson, 1862)

=*Unio jenkinsianus* Benson (1862): p. 185 [8]. Type locality: Fluvio Assamensi Berhampooter dicto [Brahmaputra River, Assam, India] [8].

=*Parreysia* (s. str.) *daccaensis* Preston (1912): p. 300 [36]. Type locality: Dacca [Dacca, Meghna Basin, Bangladesh] [36].

Type: Not traced (probably in the Benson Collection, University Museum of Zoology [UMZC], Cambridge, UK [64]).

Distribution: Ganges, Meghna, Brahmaputra, Godavari, Krishna, and Ambā basins, India and Bangladesh; a few occurrences from Bhutan [26, 65].

Comments: It is a rather well defined species. The nominal taxon *Lamellidens daccaensis*, which was considered a separate subspecies or even species due to its conchological peculiarity [15, 24, 44, 66], is treated here as a thick-shelled form of *L. jenkinsianus* based on available DNA sequence data.

Lamellidens lamellatus (Lea, 1838)

=*Unio lamellatus* Lea (1838): p. 19, pl. 6, fig. 16 [67]. Type locality: Ganges River, India [67].

Type: Holotype NMNH 85173, National Museum of Natural History, Smithsonian Institution, Washington, DC, USA (examined by us).

Distribution: Ganges, Krishna, and Mahanadi basins, India.

Comments: Here, we largely follow the traditional morphology-based concept of this species [15, 24, 28].

Lamellidens mainwaringi Preston, 1912

=*Lamellidens mainwaringi* Preston (1912): p. 306 [36]. Type locality: Siliguri [Siliguri, approx. 26.7234°N, 88.4219°E, Ganges Basin, West Bengal, India] [36].

=*Lamellidens phenchooganjensis* Preston (1912): p. 306 [36]. Type locality: Phenchooganj, Central Sylhet [Fenchuganj, 24.7014°N, 91.9424°E, Kalni River, Sylhet, Bangladesh] [36].

Type: Holotype ZSI M5016/1, Zoological Survey of India, Kolkata, India [5] (examined by us).

Distribution: Ganges, Karli, Kalni, and Kaladan rivers, India, Bangladesh, and western Myanmar.

Comments: The identity of *Lamellidens mainwaringi* is fully supported by available DNA sequence data. It is a conchologically variable species, the range of which extends through India and Bangladesh to the Kaladan River in western Myanmar. Our first reviser action on the precedence of simultaneous synonyms: *Lamellidens mainwaringi* over *L. phenchooganjensis* **syn. nov.**

Lamellidens marginalis (Lamarck, 1819)

=*Unio marginalis* Lamarck (1819): p. 79, sp. 41 [68]. Type locality: Bengale, dans les rizières [rice fields, Bengal, India] [68].

=*Symphynota bilineata* Lea (1831): p. 98, pl. 11, fig. 19 [19]. Type locality: River Hoogly, Hindostan [Hooghly River, India; the author’s note: about 100 mi upstream of Calcutta] [19].

=*Unio layardii* Lea (1859): p. 153 [69]. Type locality: Ceylon [Sri Lanka] [69].

=*Unio thwaitesii* Lea (1859): p. 152 [69]. Type locality: Ceylon [Sri Lanka] [69].

=*Unio corbeti* Deschamps (1892): p. 68, text fig. [70]. Type locality: lac de Kaudy, a Ceylan [Kandy Reservoir, 7.2921°N, 80.6392°E, Sri Lanka] [70].

Type: Syntype MNHN MPL 0031 (needs to be confirmed), Muséum National d'Histoire Naturelle, Paris, France (examined by us).

Distribution: Ganges and Krishna basins in India and Nepal [15, 23, 24]; Indus Basin in Pakistan [25]. It is thought that *Lamellidens* populations from Sri Lanka also belong to this species [15, 41, 63].

Comments: It seems to be the most popular and economically important species among Indian freshwater mussels [15]. Here, we use available DNA sequences and propose a narrower concept

of this species compared with those presented in earlier works [13, 15, 27, 28]. Its shell shape varies from narrow and elongated to broadly ovate. Three nominal species described from Sri Lanka are placed here in the synonymy of *Lamellidens marginalis* based on morphological similarity but this preliminary hypothesis needs future confirmation by means of a molecular approach.

Tribe Parreysiini Henderson, 1935

Genus *Parreysia* Conrad, 1853

The *keralaensis*-group

Parreysia keralaensis Bolotov, Pasupuleti & Subba Rao **sp. nov.**

Type: Holotype FBRC ZSI 1007-a (RCB2), Freshwater Biology Regional Centre, Zoological Survey of India, Hyderabad, Telangana, India.

Type locality: Periyar River (downstream), 10.11°N, 76.37°E, Aluva, Kerala, India.

Distribution: Periyar and Pampa basins, India.

Comments: A new species from southwestern India, which is described in this study (see Results).

The *corrugata*-group

Parreysia corrugata (Müller, 1774)

=*Mya corrugata* Müller (1774): p. 214, sp. 398 [71]. Type locality: In fluviis littoris Coromandel [rivers of the Coromandel Coast of India] [71].

=*Mya spuria* Gmelin (1791): p. 3222, sp. 16 [29]. Type locality: Tranquebariae fluviis [Kaveri Basin, Tamil Nadu, India] [29].

=*Mya gaditana* Schreibers (1793): p. 6, sp. 14 [72]. Type locality: In fluviis littoris Coromandel [rivers of the Coromandel Coast of India] [72].

=*Unio multidentatus* Philippi (1847): p. 46, pl. 3, fig. 4 [73]. Type locality: Nova Hollandia [erroneous: it was collected somewhere in India] [73].

=*Unio fulmineus* Philippi (1847): p. 46, pl. 3, figs. 5-6 [73]. Type locality: Nova Hollandia [erroneous: it was collected somewhere in India] [73].

=*Unio sikkimensis* Lea (1859): p. 151 [69]. Type locality: Sikkim, India [69].

=*Unio wynegungaensis* Lea (1860): p. 331 [39]. Type locality: Wynegunga River, Deccan, India [Wainganga River, Godavari Basin, central India] [39].

=*Unio favidens* Benson (1862): p. 188 [8]. Type locality: Assam [8].

=*Unio favidens* var. *chrysis* Benson (1862): p. 189 [8]. Type locality: River Dojora at Kareily Ghat, near Bareilly [Dojora River near Bareilly, approx. 28.36°N, 79.43°E, Ganges Basin, Uttar Pradesh, India] [8].

=*Unio favidens* var. *deltae* Benson (1862): p. 189 [8]. Type locality: River Jellinghy, in the upper part of Gangetic Delta, Bengal [Jalangi River, approx. 23.4356°N, 88.4905°E, Ganges Basin, West Bengal, India] [8].

=*Unio favidens* var. *densa* Benson (1862): p. 189 [8]. Type locality: Ganges River, above Chunar, between Allahabad and Benares [Ganges River upstream of Chunar, between Allahabad and Varanasi, approx. 25.2271°N, 82.2463°E, Uttar Pradesh, India] [8].

=*Unio favidens* var. *trigona* Benson (1862): p. 188 [8]. Type locality: Nujeebabad, in the north-west of Rohilkhund [Milini River near Najibabad, 29.6180°N, 78.3358°E, Ganges Basin, Uttar Pradesh, India] [8].

=*Unio favidens* var. *marcens* Benson (1862): p. 188 [8]. Type locality: Berhampooter River, Assam [Brahmaputra River, Assam, India] [8].

=*Unio favidens* var. *viridula* Benson (1862): p. 189 [8]. Type locality: Standing water, or "jheel", between Humeerpore and Someerpore, Bundelkhund [Betwa River near Hamirpur, approx. 25.9520°N, 80.1455°E, Ganges Basin, Uttar Pradesh, India] [8].

=*Unio laevirostris* Benson (1862): p. 191 [8]. Type locality: Tanks and streams near the Fort of Chunar, above Benares [ponds and streams near the former Fort Chunar, approx. 25.1201°N, 82.8780°E, Ganges Basin, Uttar Pradesh, India] [8].

=*Unio smaragdites* Benson (1862): p. 190 [8]. Type locality: Fluvio Berhampooter, regionis Assamensis [Brahmaputra River, Assam, India] [8].

=*Unio tripartitus* Lea (1863): p. 190 [74]. Type locality: Jillingee River, India [Jalangi River, approx. 23.4356°N, 88.4905°E, Ganges Basin, West Bengal, India] [74].

=*Unio gowhattensis* Theobald (1873): p. 208, pl. 17, fig. 4-a [75]. Type locality: Prope Gowhatti in Assam [near Guwahati, approx. 26.1792°N, 91.7334°E, Brahmaputra Basin, Assam, India] [75].

=*Unio feddeni* Theobald (1873): p. 208, pl. 17, fig. 3 [75]. Type locality: Peemgunga fluvio, Indiae centralis [Painganga River, Godavari Basin, Maharashtra, central India] [75]. Prashad [13] assumed that the type locality of this species is erroneous and that the type was collected in Burma. Theobald [75] noted that “This very peculiar and marked form <...> seems to be rare and was collected sparingly among number of fine specimens of *U. wyngungensis*, Lea, in Central India by my colleague Mr. Fedden”. Preston [27] stated that “there are specimens [of *Unio feddeni*] also in the British Museum and in the Indian Museum from the same [type] locality, the latter collected by the late Dr. W. T. Blanford”. Hence, there is no any evidence for Prashad’s hypothesis [13], and we think it should be rejected.

=*Parreysia* (*Parreysia*) *annandalei* Preston (1912): p. 302 [36]. Type locality: Gowhatty [Guwahati, approx. 26.1792°N, 91.7334°E, Brahmaputra Basin, Assam, India] [36].

=*Parreysia favidens* var. *assamensis* Preston (1912): p. 299 [36]. Type locality: Digong [an unknown place in Assam, India] [36].

Type: Not traced.

Distribution: Ganges Basin in India and Nepal; Brahmaputra, Krishna, and Godavari basins in India [15, 23, 24]; Surma River in Bangladesh (shell lots NMNH 122358 and NMNH 84065); Sri Lanka [76]; Indus Basin in Pakistan [25].

Comments: Traditionally, it was considered the most widespread and variable species within the genus, with a large number of synonyms [15, 18, 27]. Here, we propose even a broader concept of this species based on our morphological and molecular data. We found that the umbonal sculpture in this species-level clade shares a high degree of individual variation and that what was thought to be *Parreysia favidens* and related taxa could be placed within *P. corrugata*. Conversely, the DNA sequences of samples from Brahmaputra are not available, and the identity of Assamese nominal taxa needs to be checked in the future by means of a molecular approach.

Frierson [77] introduced a new species, *Parreysia robsoni* Frierson, 1927, based on a shell of *Unio pumilis* Lea, 1836 [=*Fusconaia masoni* (Conrad, 1834)] illustrated by Sowerby (Ref. [78]: pl. 36, sp. 198) and labelled “Black River, North Carolina” [holotype NHMUK 1965150, Natural History Museum, London, United Kingdom]. It was stated that this shell “is nothing at all alike to the species above [i.e. *Unio pumilis*], but is quite evidently an East-Indian shell, closely allied to the *Parreysia* (*Unio*) *radula* Benson” [77]. It is unclear which evidence Frierson [77] used to reach this decision, because the holotype of *Parreysia robsoni* has morphologically nothing to do with the Parreysiinae and this name represents a junior synonym of the Nearctic species *Fusconaia masoni* (Ambleminae).

Parreysia plagiosoma (Benson, 1862) **stat. rev.**

=*Unio plagiosoma* Benson (1862): p. 191 [8]. Type locality: Bengal [8].

=*Unio tennentii* Hanley & Theobald (1872): p. 22, pl. 45, figs. 7-9 [79]. Type locality: British India [79].

Type: Not traced (probably in the Benson Collection, University Museum of Zoology [UMZC], Cambridge, UK [64]).

Distribution: Ganges and Vaghotan basins, India.

Comments: This species is morphologically similar to *Parreysia corrugata* and *P. nagpoorensis* but shares a more ovate shell and narrower hinge plate with less serrated cardinal teeth. Usually, it shares a well-developed zigzag sculpture over the umbo.

Parreysia rakhinensis Bolotov et al., 2020

=*Parreysia rakhinensis* Bolotov et al. (2020): p. 4, figs. 2A-B [80]. Type locality: Kyeintali Stream upstream of Ohtein village, 17.9193°N, 94.5946°E, Rakhine State, Myanmar [80].

Type: Holotype RMBH biv0652_1, Russian Museum of Biodiversity Hotspots, Federal Center for Integrated Arctic Research of the Ural Branch of the Russian Academy of Sciences, Arkhangelsk, Russia (examined by us).

Distribution: Rakhine Coast, western Myanmar.

Comments: This species was recently discovered in small and medium-sized rivers at the western coast of Myanmar [80]. It shares a high degree of conchological variability, with shell shape ranging from rounded to somewhat elongated. The shell sculpture is lacking, while the umbo is not prominent.

Parreysia nagpoorensis (Lea, 1860) **stat. rev.**

=*Unio nagpoorensis* Lea (1860): p. 331 [39]. Type locality: Ambijiri Tanks, Nagpoor, Bengal, India [Ambazari Pond in Nagpur, 21.1278°N, 79.0439°E, Godavari Basin, Maharashtra, India] [39].

=*Unio merodabensis* Küster (1861): p. 233, pl. 78, fig. 4 [81]. Type locality: Bengalen, in der Provinz Merodab [Moradabad District, Ganges Basin, Uttar Pradesh, India] [41, 81].

=*Unio pinax* Benson (1862): p. 190 [8]. Type locality: Rivulo Gungun, prope Moradabad, Rohilkhund [Ramganga River near Moradabad, 28.8437°N, 78.7894°E, Ganges Basin, Uttar Pradesh, India] [8].

=*Unio triembolus* Benson (1862): p. 190 [8]. Type locality: Ramgunga River, near Morodabod, India [Ramganga River near Moradabad, 28.8437°N, 78.7894°E, Ganges Basin, Uttar Pradesh, India] [8].

=*Unio trirostris* Hanley & Musgrave (1863): pl. 2, fig. 9 [82]. Type locality: Hindostan [India] [82].

Type: Holotype ANSP 125504, Academy of Natural Sciences, Philadelphia, USA (not examined by us).

Distribution: Ganges, Krishna, Godavari, and Karli (Peethdhaval) basins, India.

Comments: This species is similar to *Parreysia corrugata* and *P. plagiosoma* but usually shares a more or less prominent umbo. The shell sculpture is rather weakly developed, the pseudocardinal teeth are massive and serrated.

The *rajahensis*-group

Parreysia rajahensis (Lea, 1841)

=*Unio rajahensis* Lea (1841): p. 30 [83]. Type locality: Rajah's Tank, Calcutta, India [83]. The latter locality seems to be inaccurate. In particular, Blanford [41] noted that he was unable to discover what tank is referred to and that the shells from the Seven Tanks in Calcutta show considerable difference from the holotype of this nominal taxon. Here, we assume that the type shell was probably collected somewhere in the Narmada River basin, e.g. from Maharaja's Tank in Jabalpur; the label of the holotype reads "Rajah's Tank, India" without reference to Calcutta.

=*Unio indicus* Sowerby (1866): pl. 40, sp. 222 [78]. Type locality: India [78].

Type: Holotype NHMUK 1965195, Natural History Museum, London, United Kingdom (examined by us).

Distribution: Upstream section of the Narmada River, India [41]. There are historical records from this river near Hoshangabad [shell lot NHMUK MP D415; approx. 22.7564°N, 77.6857°E] and Jabalpur [shell lots NHMUK 2444-03-7-1, NHMUK 539-06-1-1, NHMUK 479-06-1-1, and NHMUK 2428-03-7-1; approx. 23.1059°N, 79.9238°E]. Recent occurrences from the middle and lower reaches of the Subarnarekha River [84] are based on erroneous identification and belong to *Parreysia corrugata* (see the shell images on Figs 2-3 in the cited paper [84]).

Comments: It is a peculiar taxon, having a thick, triangular shell with massive hinge plate and prominent umbo. It cannot be mistaken conchologically with any other species in this genus. The DNA sequences of *Parreysia rajahensis* are not available.

Parreysiinae *incertae sedis*

Genus *Balwantia* Prashad, 1919

Balwantia soleniformis (Benson, 1836)

=*Anodonta soleniformis* Benson (1836): p. 750 [85]. Type locality: the hills on the N.E. Frontier of Bengal (Silhet) [Sylhet Division, Upper Meghna Basin, northeastern Bangladesh] [85]. The type locality is not stated in the protologue but the freshwater Mollusca samples described in this work are referred to as the "Silhet collection" that was collected by somebody "in the hills on the N.E. frontier of Bengal" and was bought by the Asiatic Society of Bengal in 1833 [85].

=*Margaron* (*Unio*) *bensonii* Lea (1870): p. 57 [unnecessary replacement name for *Anodonta soleniformis* Benson] [86].

Type: Not traced (probably in the Benson Collection, University Museum of Zoology [UMZC], Cambridge, UK [64]).

Distribution: Upper Brahmaputra, Upper Barak (Dhaleswari), and Upper Meghna basins, India and Bangladesh [87, 88, 89]. There is a recent occurrence from Dihing River [27.273°N, 94.802°E], a tributary of the Upper Brahmaputra in Assam (Ref. [32]: p. 18244, Fig. 2.4).

Comments: This peculiar species shares an ultra-elongate shell and digs deep burrows in hard clay [89] and even sandstone [88] sediments. The validity of *Balwantia soleniformis* is beyond doubt since its original description [18, 41, 61, 87]. Usually, it was placed within the genus *Solenaia* Conrad, 1869 [15, 28, 31, 56] but the latter genus was subdivided into several genera [90, 91, 92, 93, 94]. Bolotov et al. [80] restored *Balwantia* as a Contradentini member but they did not take adequately into consideration some diagnostic morphological and anatomical features such as unhooked glochidia and tetragenous brooding type [87, 91]. Here, we follow Pfeiffer et al. [91] who considered it as a monotypic genus, which may belong to the Parreysiinae. The DNA sequences of *Balwantia* are not available.

Supplementary Note 2. Taxonomic review of the Late Cretaceous Unionidae (Parreysiinae) from the Intertrappean Beds of the Deccan Plateau, Indian Plate

Family Unionidae Rafinesque, 1820

Subfamily Parreysiinae Henderson, 1935

Tribe Indochinellini Bolotov, Pfeiffer, Vikhrev & Konopleva, 2018

Genus *Indonaia* Prashad, 1918

†*Indonaia hunteri* (Hislop, 1860) **comb. nov.**

=†*Unio hunteri* Hislop (1860): p. 174, pl. 6, fig. 25 [95]. Type locality: Karuni, 100 miles S.S.W. of Nagpur city, Hyderabad Territory, British India [somewhere near Karanji Village, 19.8567°N, 78.3141°E, Deccan Plateau, Telangana, India] [95]. Type strata: Lameta Formation, Deccan Intertrappean Beds, Maastrichtian [95, 96].

=†*Parreysia hunteri* (Hislop, 1860). – Modell (1969): p. 11 [97].

Type: Lectotype PIMB 948 (designated by Hartman et al., 2008 [96]), Natural History Museum, London, United Kingdom (examined by us using available digital photos: Refs [96, 98]).

Comments: Medium-sized fossil species: shell length 61.0 mm, shell height 38.1 mm, shell width 25.4 mm (lectotype) [95, 96]. Modell [97] placed this taxon in the genus *Parreysia*. However, the lectotype shares very broad, prominent umbo, which remotely resembles that of the recent *Indonaia involuta*. The fossil taxon differs from *Indonaia involuta* by having a thicker shell, massive hinge plate with well-developed lateral teeth, and corrugate plication along the ventral margin [95]. Here, we consider †*Indonaia hunteri* as a possible ancestral lineage of the *Indonaia* clade, although it may also belong to a related stem group.

†*Indonaia pascoei* Prashad, 1928

=† *Indonaia pascoei* Prashad (1928): p. 311, pl. 25, figs 4-5 [99]. Type locality: “at a point situated 2 furlongs S. 10° W. of Nawapet (17°43'30" 78°23'45"), Hyderabad State (Deccan)” [ca. 400 m SSW of Nawabpet Village, 17.7177°N, 78.3933°E, Deccan Plateau, Telangana, India] [99]. Type strata: “…in a thin lenticular bed of pale calcareous Lameta shale, resting directly upon calcified hornblende granite gneiss of Archrean age, and covered by the Deccan Trap series (Upper Cretaceous or Lower Tertiary)” [Lameta Formation, Deccan Intertrappean Beds, Maastrichtian] [99].

=†*Palindonaia pascoei* (Prashad, 1928). – Modell (1969): p. 9 [97].

Type: Holotype (based on original designation); probably in the collection of Geological Survey of India, Kolkata, India [99] (not examined by us).

Comments: Small fossil species: shell length 43.5 mm, shell height 26.6 mm, shell width 14.4 mm (holotype) [99]. Prashad [99] noted that it externally resembles *Indonaia shurtleffiana*. Here, we follow the original concept of this species, which appears to be correct.

Tribe Lamellidentini Modell, 1942

Genus *Lamellidens* Simpson, 1900

†*Lamellidens carteri* (Hislop, 1860)

=†*Unio carteri* Hislop (1860): p. 175, pl. 7, fig. 28 [95]. Type locality: Karuni, 100 miles S.S.W. of Nagpur city, Hyderabad Territory, British India [near Karanji Village, 19.8567°N, 78.3141°E, Deccan Plateau, Telangana, India] [95]. Type strata: Lameta Formation, Deccan Intertrappean Beds, Maastrichtian [95, 96].

=†*Lamellidens carteri* (Hislop, 1860). – Modell (1969): p. 11 [97].

Type: Lectotype PIMB 949 (designated by Hartman et al., 2008 [96]), Natural History Museum, London, United Kingdom (examined by us using available digital photos: Refs [96, 98]).

Comments: Large fossil species: shell length 88.9 mm, shell height 43.2 mm (lectotype) [95, 96]. The original figure of the lectotype published by Hislop (Ref. [95]: pl. 7, fig. 28) is somewhat inaccurate, because this shell does not share a broad, massive umbo and strong corrugate plication at the posterior part. Externally, it strongly resembles taxa from the recent *marginalis*-group such as *Lamellidens lamellatus* and *L. marginalis* by having an elongate-ovate shell outline, a prominent but narrow wing posteriorly, and a slightly prominent umbo. Hence, we fully agree with the earlier taxonomic decision of Modell [97], who placed it in the genus *Lamellidens*. Based on conchological features, it could surely be linked to the MRCA of the crown group of this genus.

†*Lamellidens deccanensis* (J. Sowerby in Malcolmson, 1840) **comb. nov.**

=†*Unio deccanensis* J. Sowerby in Malcolmson (1840): pl. 47, figs 4-10 [100]. Type locality: Munnoor (Malcolmson 1840) [near Muthnur Village, 19.5192°N, 78.4657°E, Nirmal Hills, Telangana, India] [101]. The original description [100] states that “Fig. 6 is in limestone from the northern descent of the Sichel hills; the others are in chert from Munnoor”. The lectotype was selected from the Munnoor’s sample [96], and thus this site becames the type locality of the species. The location of Munnoor, which is a village near the top of the Mekalgandi Ghat (a part of Nirmal [=Sichel] Hills) is clarified by Newbold [101]. Malcolmson [100] presented a map of the southeast part of the Great Basaltic District of India with the location of his fossil shell site (https://www.biodiversitylibrary.org/item/111770#page/1031/mode/1up). Type strata: Lameta Formation, Deccan Intertrappean Beds, Maastrichtian [95, 96].

=†*Hyriopsis deccanensis* (J. Sowerby, 1827) [erroneous publication year]. – Modell (1969): p. 12 [97].

Type: Lectotype PIMB 947 (of rather poor quality; designated by Hartman et al., 2008 [96]), Natural History Museum, London, United Kingdom (examined by us using digital photos: Refs [96, 98]).

Comments: Large fossil species: shell length 86.4 mm, shell height 55.9 mm, shell width 43.2 mm (lectotype) [95, 96]. The pseudocardinal teeth small, lateral teeth long, slightly arched [95]. Externally, it resembles some recent species such as *Lamellidens ferrugineus* and *L. friersoni* by having a rounded shell with a broad but weakly prominent umbo and a well-developed wing. At first glance, it could represent a conchological variety of †*Lamellidens carteri*, having a shorter and rounded shell. Here, we transfer this species from *Hyriopsis* (Gonideinae: Rectidentini) to *Lamellidens* (Parreysiinae: Lamellidentini) based on conchological features.

†*Lamellidens vredenburgi* Prashad, 1921

=†*Lamellidens vredenburgi* Prashad (1921): p. 368, pl. 12, figs 1-2 [102]. Type locality: Goraha, Narbada [probably Gora Village, 1.8608°N, 73.6830°E, Narmada District, Gujarat, India] [102]. Type strata: Lameta Formation, Deccan Intertrappean Beds, Maastrichtian [95, 96].

Type: Holotype (based on original designation); probably in the collection of Geological Survey of India, Kolkata, India [102] (not examined by us).

Comments: Small fossil species: shell length 25.1 mm, shell height 15.0 mm, shell width 7.3 mm (holotype) [102]. Prashad [102] considered it as a juvenile specimen of *Lamellidens* and also noted that "The species, though nearly related to the living forms *L. marginalis* (Lam.) and *L. corrianus* (Lea), differs from either in shape, in the umbones being more prominent and the upper surface being more angulate. As already remarked it seems to come very near the ancestral form of the living species". At the time of this description, Prashad was an experienced malacologist, who examined an enormous amount of freshwater mussel shells from British India and British Burma, and worked on a complete revision of freshwater mussels from Burma [13]. Hence, the original generic assignment of this fossil taxon appears to be correct. Though this species may represent a juvenile specimen of †*Lamellidens carteri*, a final solution on its taxonomic status requires an expanded series of fossil shells from the type locality, which is currently not available to us. Here, we follow the original concept of this species.

Tribe Parreysiini Henderson, 1935

Genus *Parreysia* Conrad, 1853

†*Parreysia imbricatus* (Hislop, 1860) **comb. nov.**

=†*Unio imbricatus* Hislop (1860): p. 175, pl. 7, fig. 27a-c [95]. Type locality: Mekalgandi Ghat, 150 miles S.S.W. of Nagpur city, Hyderabad Territory, British India [95, 100] [near Muthnur Village, 19.5192°N, 78.4657°E, Nirmal Hills, Telangana, India] [100, 101]. The lectotype of this species was collected by Malcolmson at the Mekalgandi Ghat (part of the Nirmal Hills) near Munnoor [now Muthnur] Village [100, 101], which is situated approximately 120 miles SSW of Nagpur. Type strata: Lameta Formation, Deccan Intertrappean Beds, Maastrichtian [95, 96].

=†*Schistodesmus imbricatus* (Hislop, 1860). – Modell (1969): p. 10 [97].

Type: Lectotype PIMB 950 (designated by Hartman et al., 2008 [96]), Natural History Museum, London, United Kingdom (examined by us using digital photos: Refs [96, 98]).

Comments: Medium-sized fossil species: shell length 58.4 mm, shell height 55.9 mm (lectotype) [95, 96]. The teeth structure is unknown. This species shares a triangular shell with a massive, prominent umbo. There is a prominent radial ridge from the umbo to the ventral margin, which is located at the posterior part of the shell and bears a few regular tubercles of ovate or rounded shape. Based on the latter feature, it may represent a shortened form of †*Parreysia mamillatus* (see below). Here, we transfer this species from *Schistodesmus* (Unioninae: Unionini) to *Parreysia* (Parreysiinae: Parreysiini) based on conchological features.

†*Parreysia malcolmsoni* (Hislop, 1860)

=†*Unio tumida* J. Sowerby in Malcolmson (1840): pl. 47, figs 11-12 [unavailable name as a primary homonym] [100]. Type locality: Mekalgandi Ghat, 150 miles S.S.W. of Nagpur city, Hyderabad Territory, British India [95, 100] [near Muthnur Village, 19.5192°N, 78.4657°E, Nirmal Hills, Telangana, India] [100, 101]. The lectotype of this species was collected by Malcolmson at the Mekalgandi Ghat (part of the Nirmal Hills) near Munnoor [now Muthnur] Village [100, 101], which is situated approximately 120 miles SSW of Nagpur. Type strata: Lameta Formation, Deccan Intertrappean Beds, Maastrichtian [95, 96].

=†*Unio malcolmsoni* Hislop (1860): p. 174 [new name for †*Unio tumida* Sowerby in Malcolmson, 1840] [95].

=†*Parreysia malcolmensis* Modell (1969): p. 11 [error for †*Unio malcolmsoni* Hislop, 1860] [97].

Type: Lectotype PIMB 953 (complete shell; designated by Hartman et al., 2008 [96]), Natural History Museum, London, United Kingdom (examined by us using digital photos: Refs [96, 98]).

Comments: Small fossil species: shell length 20.3 mm, shell height 17.8 mm, shell width 16.5 mm (lectotype) [95, 96]. The teeth structure is unknown. It shares a triangular, inequilateral shell with a

slightly tapering posterior margin and a prominent umbo that conchologically corresponds to the genus *Parreysia*. Based on external features, this fossil species clearly resembles the recent *Parreysia rajahensis*.

†*Parreysia mamillatus* (Hislop, 1860) **comb. nov.**

=†*Unio mamillatus* Hislop (1860): p. 175, pl. 7, fig. 26 [95]. Type locality: Karuni, 100 miles S.S.W. of Nagpur city, Hyderabad Territory, British India [near Karanji Village, 19.8567°N, 78.3141°E, Deccan Plateau, Telangana, India] [95]. Type strata: Lameta Formation, Deccan Intertrappean Beds, Maastrichtian [95, 96].

=†*Schistodesmus mamillatus* (Hislop, 1860). – Modell (1969): p. 10 [97].

Type: Lectotype PIMB 953 (complete shell; designated by Hartman et al., 2008 [96]), Natural History Museum, London, United Kingdom (examined by us using digital photos: Refs [96, 98]).

Comments: Large fossil species: shell length 99.1 mm, shell height 58.4 mm, shell width 40.6 mm [95, 96]. The teeth structure is unknown. It is very similar to †*Parreysia imbricatus* based on some conchological features such as a massive, prominent umbo and a radial ridge bearing a few regular tubercles but could be distinguished from it by having a more elongated, ovate shell (vs shorter and triangular). In general, †*Parreysia mamillatus* and †*P. imbricatus* may represent intraspecific conchological varieties, reflecting environment-induced ecophenotypic plasticity, e.g. lacustrine and riverine forms [103, 104, 105, 106]. Here, we transfer this species from *Schistodesmus* (Unioninae: Unionini) to *Parreysia* (Parreysiinae: Parreysiini) based on conchological features.

Supplementary Note 3. Taxonomic review of doubtful and uncertain freshwater mussel taxa from India described by Constantine S. Rafinesque

The nominal genus *Diplasma* Rafinesque, 1831 nom. dub.

Type species: *Diplasma marginata* Rafinesque, 1831 (type locality: uncertain; River Tennessee or Hindostan) (subsequent designation by Frierson, 1914 [107]).

Comments: Rafinesque [20] noted that the type specimen of *Diplasma marginata* is “from the river Tennessee, as stated to Mr. Hembel, but so near the next [i.e. *Diplasma similis* Rafinesque, 1831], that the fact appears doubtful to me; perhaps the locality has been erroneously stated or labelled in Mr. Lea's cabinet, from whence the shell is said to have come, and it may be also a shell from Hindostan”. It was introduced as follows: “shell thin, elliptical, swelled, back horizontal, sloping and truncate obliquely behind; outside very smooth, shining brown, anterior and interior margin yellowish, inside pale incarnate. <...> Lamellar tooth properly curved, the anterior pretty long. Size of the shell over two inches” [20]. Based on this description, the type specimen of *Diplasma marginata* may belong to *Sagittunio subrostratus* (Say, 1831), a widespread Nearctic species, which also occurs in the Tennessee River [9, 108]. If our assumption is true, *Sagittunio* Watters, 2018 becomes a junior synonym of *Diplasma*. In its turn, *Diplasma similis* Rafinesque, 1831 (type locality: River Ganges) [20] appears to be a member of the genus *Lamellidens*, because it was described as having “shell very thin, elliptical, not swelled, back horizontal, truncate obliquely behind, hardly sloping, outside smooth, dark olivaceous, with a pale margin, inside bluish incarnate. <...> teeth <...> forming almost an angle rather than a curved arch, anterior tooth shorter, oblique, the posterior perfectly horizontal. Length nearly two inches” [20]. In all the cases, mentioned above, the final taxonomic decision cannot be made in the absence of the type specimens. Hence, we here consider *Diplasma*, *D. marginata*, and *D. similis* as *nomina dubia* until a more convincing evidence on their taxonomic placement will be available. The associated family-group name Diplasminae Modell, 1942 [109] also becomes a *nomen dubium*.

The nominal genus *Hemisolasma* Rafinesque, 1831 nom. dub.

Type species: *Diplasma* (*Hemisolasma*) *vitrea* Rafinesque, 1831 (type locality: River Jellinghy in Bengal [approx. 23.4356°N, 88.4905°E, Jalangi River, West Bengal, India]) (subsequent designation by Baker, 1964: p. 141 [110]; the same type species was designated again by Graf & Cummings, 2006: p. 393 [111]).

Comments: The type species of this genus was traditionally considered a synonym of *Indonaia olivaria* (Lea, 1831) [77, 110, 111]. Frierson [77] argued that Dr. Burroughs (a shell collector) separated his Indian samples between C. Rafinesque and I. Lea and that the two authors simultaneously described the same species under two different names. However, this hypothesis is not so straightforward, because the nominal species *Diplasma* (*Hemisolasma*) *vitrea* cannot be identified with certainty in the absence of the type shell. Based on the brief protologue, its type specimen could equally be considered a juvenile *Lamellidens*: “Shell very thin and brittle, almost transparent, oval swelled, broader behind, with a slope outside, very smooth, greenish, or fulvescent, inside whitish, teeth subequal. <...> Small, hardly over inch, fine delicate shell” [20]. If so, *Lamellidens* becomes a junior synonym of *Hemisolasma*. Conversely, we cannot propose a final solution on this

taxonomic puzzle without examination of the type shell, and here consider both *Hemisolasma* and its type species as *nomina dubia*. This also applies to the corresponding family-group taxon, the Hemisolasminae Starobogatov, 1970 [112]. In its turn, the nominal species *Diplasma* (*Hemisolasma*) *striata* Rafinesque, 1831 (type locality: River Jellinghy in Bengal [approx. 23.4356°N, 88.4905°E, Jalangi River, West Bengal, India]) is considered here a junior synonym of *Indonaia rugosa* (Gmelin, 1791) **comb. nov.** (see Table 1 in the main text of this paper) based on the original description: “Shell thick, suboval, swelled, behind sloping subtruncate and transversally striated, outside olivaceous greenish, smooth below, but longitudinally striated above; strias in a zigzag form in the middle, inside silvery white, teeth subequal, much curved. <...> Small, hardly one inch” [20].

The nominal genus *Lampsilis* Rafinesque, 1820 (erroneous generic assignment)

Comments: Two new nominal species in this genus were described from the River Ganges: *Lampsilis argyratus* Rafinesque, 1831 and *L. fulgens* Rafinesque, 1831 [20]. The first nominal species is considered here as a junior synonym of *Indonaia caerulea* (Lea, 1831) based on the original description [20]: “Shell thin, elliptical, swelled, attenuated behind, outside laminated greenish, decoricated and silvery at summit, inside bluish iridescent. <...> Size one and a-half inch. Very near to the S. G. *Leptodea*, but teeth as in the last [i.e. *Lampsilis fulgens*], cardinal small crenulate, lamellar less flexuose, not crenulate” (see Table 1 in the main text of this paper). As for the second species, its diagnostic features are rather unclear: “shell thick, elliptical, swelled, attenuated behind, outside nearly smooth, laminated, ferruginous brown; inside of a beautiful metallic incarnate and iridescent. <...> two or three inches, beautiful shell, a true *Lampsilis*, with a long flexuose lamellar tooth subcrenulate; cardinal tooth compressed crenulate” [20]. It could be either a *Parreysia* or an *Indonaia* species. The decision on its taxonomic placement could only be made on the basis of examination of the type, which appears to be lost, and here we consider *Lampsilis fulgens* as *nomen dubium*.

The nominal genus *Loncosilla* Rafinesque, 1831 syn. nov.

Type species: *Loncosilla solenoides* Rafinesque, 1831 (type locality: River Jellinghy in Bengal [approx. 23.4356°N, 88.4905°E, Jalangi River, West Bengal, India]).

Comments: It is a monotypic genus. This taxon was described as follows: “Shell transversal, unequilateral, somewhat gaping, only one muscular impression anteriorly. No teeth as in *Anodonta*, but a hinge with a marginal nerve, or fold anteriorly; <...> A distinct genus of the tribe Anodonta, which had been mistaken for a fluviatile *Solen* by Dr. Burroughs the discoverer of it; but all *Solens* are marine shells. The name means little knife: it is different from all my S.G. of *Anodonta*. <...> both ends rounded and a little gaping, back horizontal, outside and inside smooth and whitish. <...> Small, seldom one inch long” [20]. Based on these diagnostic features, one should conclude that *Loncosilla* is not a unionid mussel but a freshwater clam of the family Pharidae [113, 114]. Based on morphological and geographic evidence, the formal synonymy is proposed here as follows: *Novaculina* Benson, 1830 [=*Loncosilla* Rafinesque, 1831 **syn. nov.**] and *Novaculina gangetica* Benson, 1830 [=*Loncosilla solenoides* Rafinesque, 1831 **syn. nov.**] (Pharidae: Pharellinae). Thiele [115] already proposed the synonymy of the two genera but it was published with a question mark and did not contain any explanatory remarks. The year of *Loncosilla* description in Thiele’s catalogue [115] is given incorrectly (as 1820).

Supplementary References

1. Matzke, N. J. Model selection in historical biogeography reveals that founder-event speciation is a crucial process in island clades. *Systematic Biology* **63**, 951–970; https://doi.org/10.1093/sysbio/syu056 (2014).

2. Matzke, N. J. Probabilistic historical biogeography: new models for founder-event speciation, imperfect detection, and fossils allow improved accuracy and model-testing. *Frontiers of Biogeography* **5**, 242–248; https://doi.org/10.21425/F5FBG19694 (2013).

3. Yu, Y., Blair, C. & He, X. J. RASP 4: Ancestral State Reconstruction Tool for Multiple Genes and Characters. *Molecular Biology and Evolution* **37**, 604–606; https://doi.org/10.1093/molbev/msz257 (2020).

4. Nevill, G. List of the Mollusca brought back by Dr. J. Anderson from Yunnan and Upper Burma, with descriptions of new species. *Journal of the Asiatic Society of Bengal* **46**, 14–41 (1877).

5. Ramakrishna, Dey, A. & Mitra, S. C. Catalogue of type species (Bivalvia, Scaphopoda) present in the Mollusca section of Zoological Survey of India. *Records of the Zoological Survey of India (Occasional Paper)* **228**, 1–97 (2004).

6. Bolotov, I. N. et al. *Indonaia rectangularis* (Tapparone-Canefri, 1889), comb. nov., a forgotten freshwater mussel species from Myanmar. *ZooKeys* 852, 23–30; https://doi.org/10.3897/zookeys.852.33898 (2019).

7. Eydoux, F. Mollusques. *Magasin de Zoologie* **8**, 181–192 (1838).

8. Benson, W. H. Descriptions of Indian and Burmese species of the genus *Unio*, Retz. *Annals and Magazine of Natural History* **10**, 184–195 (1862).

9. Graf, D. L. & Cummings, K. S. A 'big data' approach to global freshwater mussel diversity (Bivalvia: Unionoida), with an updated checklist of genera and species. *Journal of Molluscan Studies* **87**, eyaa034; https://doi.org/10.1093/mollus/eyaa034 (2021).

10. Zieritz, A. et al. Diversity, biogeography and conservation of freshwater mussels (Bivalvia: Unionida) in East and Southeast Asia. *Hydrobiologia* **810**, 29–44; https://doi.org/10.1007/s10750-017-3104-8 (2018).

11. Tapparone-Canefri, C. Viaggio de Leonardo Fea in Birmania e regioni vicine. XVIII. Molluschi terrestri e d'acqua dolce. *Annali del Museo Civico di Storia Naturale de Genova (series 2)* **27**, 295–359 (1889).

12. Annandale, N. & Prashad, B. XXVIII. The aquatic and amphibious Mollusca of Manipur. *Records of the Indian Museum* **22**, 529–631 (1921).

13. Prashad, B. A revision of the Burmese Unionidae. *Records of the Indian Museum* **24**, 91–111 (1922).

14. Prashad, B. Pelecypoda of the Indawgyi Lake and of its connected freshwater areas in the Myitkyina District, Upper Burma. *Records of the Indian Museum* **32**, 247–255 (1930).

15. Subba Rao, N. V. *Handbook. Freshwater molluscs of India* (Zoological Survey of India, 1989).

16. Whelan, N. V., Geneva, A. J. & Graf, D. L. Molecular phylogenetic analysis of tropical freshwater mussels (Mollusca: Bivalvia: Unionoida) resolves the position of *Coelatura* and supports a monophyletic Unionidae. *Molecular Phylogenetics and Evolution* **61**, 504–514; https://doi.org/10.1016/j.ympev.2011.07.016 (2011).

17. Pfeiffer, J.M. & Graf, D.L. Evolution of bilaterally asymmetrical larvae in freshwater mussels (Bivalvia: Unionoida: Unionidae). *Zoological Journal of the Linnean Society* **175**, 307–318; https://doi.org/10.1111/zoj.12282 (2015).

18. Haas, F. Superfamilia Unionacea. *Das Tierreich* **88**, 1–663 (1969).

19. Lea, I. Observations on the Naïades, and descriptions of new species of that and other families. *Transactions of the American Philosophical Society* **4**, 63–121 (1831).

20. Rafinesque, C. S. *Continuation of a monograph of the bivalve shells of the river Ohio and other rivers of the western states. By Prof. C.S. Rafinesque. (Published at Brussels, September, 1820). Containing 46 species, from No. 76 to no. 121. Including an appendix on some bivalve shells of the rivers of Hindostan, with a supplement on the fossil bivalves of the Western States, and the Tulosites, a new genus of fossils* (Philadelphia, 1831).

21. Lea, I. Description of twenty-five new species of exotic uniones. *Proceedings of the Academy of Natural Sciences of Philadelphia* **8**, 92–95 (1856).

22. Lea, I. Description of five new species of the genus *Unio*. *Proceedings of the Academy of Natural Sciences of Philadelphia* **18**, 133 (1866).

23. Nesemann, H. A., Sharma, S. U., Sharma, G. O. & Sinha, R. K. Illustrated checklist of large freshwater bivalves of the Ganga River system (Mollusca: Bivalvia: Solecurtidae, Unionidae, Amblemidae). *Nachrichchtenblatt der Ersten Vorarlberger Malakologischen Gesellschaft* **13**, 1–51 (2005).

24. Nesemann, H. et al. *Aquatic Invertebrates of the Ganga River System: Volume 1 – Mollusca, Annelida, Crustacea (in part)* (Hasko Nesemann and Chandi Press, 2007).

25. Burdi, G. H., Baloch, W. A., Begum, F., Soomro A. N. & Khuhawar M. Y. Ecological studies on freshwater bivalve mussels (Pelecypoda) of Indus River and its canals at Kotri Barrage Sindh, Pakistan. *Sindh University Research Journal* **41**, 31–36 (2009).

26. Gittenberger, E., Leda, P., Gyeltshen, C. & Sherub, S. Distributional patterns of molluscan taxa in Bhutan (Mollusca). *Biodiversität und Naturausstattung im Himalaya* **4**, 143–151 (2018).

27. Preston, H. B. *Mollusca (Freshwater Gastropoda & Pelecypoda). Fauna of British India, including Ceylon and Burma* (Taylor and Francis, 1915).

28. Ramakrishna & Dey, A. *Handbook on Indian freshwater molluscs* (Zoological Survey of India, 2007).

29. Gmelin, J. F. *Systema Naturae per Regna Tria Naturae, Secundum Classes, Ordines, Genera, Species, cum Characteribus, Differentiis, Synonymis, locis* **1(6)**, 3021–3909 (1791).

30. Hanley, S. Appendix, containing descriptions of the shells delineated in the plates, yet not described in the text; with a systematic list of the engravings, etc. in *An Illustrated and Descriptive Catalogue of Recent Bivalve Shells* 335–389 (Williams and Norgate, 1856).

31. Simpson, C. T. Synopsis of the naiades, or pearly fresh-water mussels. *Proceedings of the United States National Museum* **22**, 501–1044 (1900).

32. Sonowal, J., Puzari, M. & Kardong, D. Diversity of freshwater molluscs from the Upper Brahmaputra Basin, Assam, India. *Journal of Threatened Taxa* **13**, 18237–18246; https://doi.org/10.11609/jott.7144.13.5.18237-18246 (2021).

33. Ray, H. C. A new species of freshwater mussel, *Indonaia khadakvaslaensis*, from Poona, Maharashtra, India (Mollusca: Bivalvia: Family Unionidae). *Journal de Conchyliologie* **105**, 226–229 (1966).

34. Martens, E. v. Binnen-Conchylien aus Ober-Birma. *Archiv für Naturgeschichte* **65**, 30–48 (1899).

35. Konopleva, E. S. et al. A new genus and two new species of freshwater mussels (Unionidae) from Western Indochina. *Scientific Reports* **9**, 4106; https://doi.org/10.1038/s41598-019-39365-1 (2019).

36. Preston, H. B. A catalogue of the Asiatic naiades in the collection of the Indian Museum, Calcutta, with descriptions of new species. *Records of the Indian Museum* 7, 279–308 (1912).

37. Annandale, N. & Prashad, B. Some freshwater molluscs from the Bombay Presidency. *Records of the Indian Museum* **16**, 139–152 (1919b).

38. Philippi, R. A. *Unio*. Tab. I. *Abbildungen und Beschreibungen neuer oder wenig gekannter Conchylien* **1**, 19–20 (1843).

39. Lea, I. Descriptions of three new species of exotic uniones. *Proceedings of the Academy of Natural Sciences of Philadelphia* **11**, 331 (1860).

40. Preston, H. B. VIII. Descriptions of new species of marine and freshwater shells in the collection of the Indian Museum, Calcutta. *Records of the Indian Museum* **2**, 45–48 (1908).

41. Blanford, W. T. Contributions to Indian Malacology no VII. List of species of *Unio* and *Anodonta* described as occurring in India, Ceylon and Burma. 1. *Journal of the Asiatic Society of Bengal* **35**, 134–155 (1866).

42. Theobald, W. Descriptions of some new land and freshwater shells from India and Burmah. *Journal of the Asiatic Society of Bengal* **45**, 184–189 (1876).

43. Haas, F. Eine neude indische Najade, *Trapezoideus prashadi*. *Senckenbergiana* **4**, 101–102 (1922).

44. Prashad, B. Notes on lamellibranchs in the Indian Museum. *Records of the Indian Museum* **19**, 165–173 (1920).

45. Madhyastha, N. A. & Mumbrekar, K. D. Two endemic genera of bivalves in the Tunga River of the Western Ghats, Karnataka, India. *Tentacle* **14**, 23–24 (2006).

46. Aravind, N. A. et al. The status and distribution of freshwater molluscs of the Western Ghats. in *The status and distribution of freshwater biodiversity in the Western Ghats, India* (comps Molur, S., Smith, K. G., Daniel, B. A. & Darwall, W. R. T.) 21–42 (IUCN and Zoo Outreach Organisation, 2011).

47. Lea, I. Observations on the Naïades; and descriptions of new species of that and other families. *Transactions of the American Philosophical Society* **5**, 23–119 (1834).

48. Bolotov, I. N. et al. New taxa of freshwater mussels (Unionidae) from a species-rich but overlooked evolutionary hotspot in Southeast Asia. *Scientific Reports* **7**, 11573; https://doi.org/10.1038/s41598-017-11957-9 (2017b).

49. Konopleva E. S., Bolotov, I. N., Vikhrev I. V., Gofarov, M. Y., Kondakov, A. V. An integrative approach underscores the taxonomic status of *Lamellidens exolescens*, a freshwater mussel from the Oriental tropics (Bivalvia: Unionidae). *Systematics and Biodiversity* **15**, 204–217; https://doi.org/10.1080/14772000.2016.1249530 (2017).

50. Konopleva, E. S. et al. A taxonomic review of *Trapezidens* (Bivalvia: Unionidae: Lamellidentini), a freshwater mussel genus endemic to Myanmar, with a description of a new species. *Ecologica Montenegrina* **27**, 45–57; https://doi.org/10.37828/em.2020.27.6 (2020).

51. Bolotov, I. N. et al. Ancient river inference explains exceptional Oriental freshwater mussel radiations. *Scientific Reports* **7**, 2135; https://doi.org/10.1038/s41598-017-02312-z (2017a).

52. Hutton, T. Notices of some land and fresh water shells occurring in Afghanistan. *Journal of the Asiatic Society of Bengal* **18**, 649–661 (1849).

53. Annandale, N. & Prashad, B. The Mollusca of the inland waters of Baluchistan and of Seistan. *Records of the Indian Museum* **18**, 17–62 (1919a).

54. Annandale, N. Aquatic molluscs of the Inlé Lake and connected waters. *Records of the Indian Museum* **14**, 103–182 (1918).

55. Sowerby, G. B. Genus *Unio*. *Conchologica Iconica* **16**, pls. 1, 61–96 (1868).

56. Simpson, C. T. *A descriptive catalogue of the naiades, or pearly fresh-water mussels. Parts I-III* (Bryant Walker, 1914).

57. Graf, D. L. & Cummings, K. S. Review of the systematics and global diversity of freshwater mussel species (Bivalvia: Unionoida). *Journal of Molluscan Studies* **73**, 291–314; https://doi.org/10.1093/mollus/eym029 (2007).

58. Bolotov, I. N. et al. Spreading of the Chinese pond mussel, *Sinanodonta woodiana*, across Wallacea: One or more lineages invade tropical islands and Europe. *Biochemical Systematics and Ecology* **67**, 58–64; https://doi.org/10.1016/j.bse.2016.05.018 (2016).

59. Haas, F. Die Unioniden. *H.C. Küster, Systematisches Conchylien-Cabinet von Martini und Chemnitz* **9**, 257–288 (1919).

60. Haas, F. Die Unioniden. *H.C. Küster, Systematisches Conchylien-Cabinet von Martini und Chemnitz* **9**, 289–304 (1920).

61. Prashad, B. Notes on some little known South Asiatic Unionidæ. *Proceedings of the Malacological Society of London* **20**, 174–178 (1932).

62. Gould, A. A. D. Gould described new shells, received from Rev. Mr. Mason, of Burmah. *Proceedings of the Boston Society of Natural History* **2**, 218–221 (1847).

63. Mendis, A. S., Fernando, C. H. & Kariyawasam, G. D. A guide to the freshwater fauna of Ceylon. *Bulletin of the Fisheries Research Station, Ceylon* **12**, 1–160 (1962).

64. Preece, R.C. et al. William Benson and the Golden Age of Malacology in British India. *Tropical Natural History* **22**, In Press. (2022).

65. Gittenberger et al. *A Field Guide to the Common Molluscs of Bhutan* (National Biodiversity Centre (NBC), Ministry of Agriculture and Forests, 2017).

66. Pasupuleti, R. & Subba Rao, N. V. A report on the extended distribution of a rare Indian freshwater mussel species (Mollusca: Bivalvia: Unionidae). *Advances in Applied Science Research* **6**, 162–165. (2015).

67. Lea, I. Description of new freshwater and land shells. *Transactions of the American Philosophical Society* **6**, 1–154 (1838).

68. Lamarck, J.-B. *Histoire naturelle des animaux sans vertèbres. Vol. 6* (Chez l'Auteur, 1819).

69. Lea, I. Descriptions of twenty-one new species of exotic Unionidae. *Proceedings of the Academy of Natural Sciences of Philadelphia* **11**, 151–154 (1859).

70. Deschamps, E. Description d'une nouvelle espèce d'*Unio* de Ceylan. *Bulletin de la Société Zoologique de France* **17**, 68–69 (1892).

71. Müller, O. F. Testacea. *Vermivm Terrestrium et Fluviatilium, Seu Animalium Infusoriorum, Helminthicorum et Testaceorum, non Marinorum, Succincta Historia* **2**, 1–214 (1774).

72. Schreibers, K. *Versuch einer vollständigen Conchylienkenntniss nach Linnes System. Zweyter Band: von den Muscheln* (Joseph Edlen v. Kurzbeck, 1793).

73. Philippi, R. A. *Unio*. Tab. III. *Abblildungen und Beschreibungen neuer oder wenig gekannter Conchylien* **3**, 45–47 (1847).

74. Lea, I. Descriptions of eleven new species of exotic Unionidae. *Proceedings of the Academy of Natural Sciences of Philadelphia* **15**, 189–190 (1863).

75. Theobald, W. Descriptions of new species of Unionidae. *Journal of the Asiatic Society of Bengal* **42**, 207–209 (1873).

76. Hadl, G. Results of the Austrian-Ceylonese Hydrobiological Mission 1970 of the 1st Zoological Institute of the University of Vienna (Austria) and the Department of Zoology of the Vidyalankara University of Ceylon, Kelaniya. Part XVIII: Freshwater Mussels Bivalvia. *Bulletin of the Fisheries Research Station Sri Lanka (Ceylon)* **25**, 183–188 (1974).

77. Frierson, L. S. *A Classified and Annotated Check List of the North American Naiades* (Baylor University Press, 1927).

78. Sowerby, G. B. Genus *Unio*. *Conchologica Iconica* **16**, pls. 31–54 (1866)

79. Hanley, S. & Theobald, W. *Conchologia Indica: Illustrations of the Land and Freshwater Shells of British India* **3**, 19–28 (1872).

80. Bolotov, I. N. et al. New freshwater mussel taxa discoveries clarify biogeographic division of Southeast Asia. *Scientific Reports* **10**, 6616; https://doi.org/10.1038/s41598-020-63612-5 (2020).

81. Küster, H. C. Die Flussperlmuscheln (*Unio* et *Hyria*). *Systematisches Conchylien-Cabinet von Martini und Chemnitz Fortgesetzt von Hofrath Dr. G. v. Schubert und Professor Dr. J. A. Wagner* **9**, 161–256 (1861).

82. Hanley, S. & Musgrave, S. *Photographic Conchology: A Second, Or Photographic Series, of the Conchological Miscellany. The Photographs, Forming a Sequence to the Index Testaceologicus (Hanley's Edition), Illustrative of Species Either Not Delineated in the More Common Works, Or Distinguished for Rarity* (Willis and Sotheran, 1863).

83. Lea, I. Continuation of paper on fresh water and land shells. *Proceedings of the American Philosophical Society* **2**, 30–34 (1841).

84. Sharma, G., Nesemann, H., Sharma, S. Discovery of a population of a virtually unknown large freshwater bivalve *Parreysia rajahensis* (Lea, 1841) (Unionoidea: Amblemidae) in the River Subernarekha, India. *Journal of Environment and Sociobiology* **6**, 195–204 (2009).

85. Benson, W. H. Descriptive catalogue of a collection of land and fresh-water shells, chiefly contained in the museum of the Asiatic Society. *Journal of the Asiatic Society of Bengal* **5**, 741–750 (1836).

86. Lea, I. *A Synopsis of the Family of Naïades. Fourth Edition, Very Greatly Enlarged and Improved.* (Henry C. Lea, 1870).

87. Prashad, B. Studies on the anatomy of Indian Mollusca. The soft parts of some Indian Unionidae. *Records of the Indian Museum* **16**, 289–296 (1919).

88. Annandale, N. Further note on the burrows of *Solenaia soleniformis*. *Records of the Indian Museum* **16**, 205–206 (1919).

89. Godwin–Austen, H. H. Description of a new species of *Margaritanopsis* (Unionidae) from the Southern Shan States, with notes on *Solenaia soleniformis*. *Records of the Indian Museum* **16**, 203–205 (1919).

90. Pfeiffer, J. M., Breinholt, J. W. & Page, L. M. Unioverse: a phylogenetic resource for reconstructing the evolution of freshwater mussels (Bivalvia, Unionoida). *Molecular Phylogenetics and Evolution* **137**, 114–126; https://doi.org/10.1016/j.ympev.2019.02.016 (2019).

91. Pfeiffer, J. M., Graf, D. L., Cummings, K. S. & Page, L. M. Taxonomic revision of a radiation of South-East Asian freshwater mussels (Unionidae: Gonideinae: Contradentini+ Rectidentini). *Invertebrate Systematics* **35**, 394–470; https://doi.org/10.1071/IS20044 (2021).

92. Huang, X.-C. et al. Towards a global phylogeny of freshwater mussels (Bivalivia: Unionida): Species delimitation of Chinese taxa, mitochondrial phylogenomics, and diversification patterns. *Molecular Phylogenetics and Evolution* **130**, 45–59; https://doi.org/10.1016/j.ympev.2018.09.019 (2019).

93. Lopes-Lima, M. et al. Freshwater mussels (Bivalvia: Unionidae) from the Rising Sun (Far East Asia): phylogeny, systematics, and distribution. *Molecular Phylogenetics and Evolution* **146**, 106755; https://doi.org/10.1016/j.ympev.2020.106755 (2020).

94. Bolotov, I. N., Kondakov, A. V., Konopleva, E. S. & Vikhrev, I. V. A new genus of ultra-elongate freshwater mussels from Vietnam and eastern China (Bivalvia: Unionidae). *Ecologica Montenegrina* **39**, 1–6; https://doi.org/10.37828/em.2021.39.1 (2021).

95. Hislop, S. Description of fossil shells, from the above-described deposits. *The Quarterly Journal of the Geological Society of London* **16**, 166–181 (1860).

96. Hartman, J. H., Erickson, D. N. & Bakken, A. Stephen Hislop and his 1860 Cretaceous continental molluscan new species descriptions in Latin from the Deccan Plateau, India. *Palaeontology* **51**, 1225–1252; https://doi.org/10.1111/j.1475-4983.2008.00807.x (2008).

97. Modell, H. Paläontologische und geologische Untersuchungen im Tertiär von Pakistan. 4. Die tertiären Najaden des Punjab und Vorderindiens. *Abhandlungen der Bayerischen Akademie der Wissenschaften, Mathematisch-naturwissenschaftliche Klasse, neue Folge* **135**, 1–49 (1969).

98. Bingle-Davis, M. J. *Systematics, diversity, and origins of Upper Cretaceous continental molluscan fauna in the infra- and intertrappean strata of the Deccan Plateau, central India (PhD Dissertation)* (University of North Dakota, 2012).

99. Prashad, B. On some Fossil Indian Unionidae. *Records of the Geological Survey of India* **60**, 308–312 (1928).

100. Malcolmson, J. G. XXXVIII. - On the Fossils of the Eastern Portion of the Great Basaltic District of India. *Transactions of the Geological Society of London (2nd series)* **5**, 537–575 (1840).

101. Newbold, C. Summary of the Geology of Southern India. Part V. Fresh-water Limestones and Cherts. *Journal of the Royal Asiatic Society of Great Britain and Ireland* **8**, 219–227 (1846).

102. Prashad, B. On a new fossil unionid from the intertrappean beds of Peninsular India. *Records of the Geological Survey of India* **51**, 368–370 (1921).

103. Hornbach, D. J., Kurth, V. J. & Hove, M. C. Variation in freshwater mussel shell sculpture and shape along a river gradient. *American Midland Naturalist* **164**, 22–36 (2010).

104. Inoue, K., Hayes, D. M., Harris, J. L. & Christian, A. D. Phylogenetic and morphometric analyses reveal ecophenotypic plasticity in freshwater mussels *Obovaria jacksoniana* and *Villosa arkansasensis* (Bivalvia: Unionidae). *Ecology and Evolution* **3**, 2670–2683 (2013).

105. Lyubas, A. A. et al. A taxonomic revision of fossil freshwater pearl mussels (Bivalvia: Unionoida: Margaritiferidae) from Pliocene and Pleistocene deposits of Southeastern Europe. *Ecologica Montenegrina* **21**, 1–16; https://doi.org/10.37828/em.2019.21.1 (2019).

106. Watters, G. T. Form and function of unionoidean shell sculpture and shape (Bivalvia). *American Malacological Bulletin* **11**, 1–20 (1994).

107. Frierson, L. S. Remarks on classification of the Unionidae. *Nautilus* **28**, 6–8 (1914).

108. Watters, G. T. A preliminary review of the nominal genus *Villosa* of freshwater mussels (Bivalvia, Unionidae) in North America. *Visaya, Supplement* **10**, 1–140 (2018).

109. Modell, H. Das natlirliche System der Najaden. *Archiv für Molluskenkunde* **74**, 161–191 (1942).

110. Baker, H. B. Some of Rafinesque's unionid names. *The Nautilus* **77**, 140–142 (1964).

111. Graf, D. L. & Cummings, K. S. Palaeoheterodont diversity (Mollusca: Trigonioida+ Unionoida): what we know and what we wish we knew about freshwater mussel evolution. *Zoological Journal of the Linnean Society* **148**, 343–394; https://doi.org/10.1111/j.1096-3642.2006.00259.x (2006).

112. Starobogatov, Y. I. *Fauna of molluscs and zoogeographic division of continental waterbodies of the globe* (Nauka, 1970).

113. Bolotov, I. N. et al. Discovery of *Novaculina myanmarensis* sp. nov. (Bivalvia: Pharidae: Pharellinae) closes the freshwater razor clams range disjunction in Southeast Asia. *Scientific Reports* **8**, 16325; https://doi.org/10.1038/s41598-018-34491-8 (2018).

114. Than Win et al. Phylogeography and distribution of the freshwater razor clams *Novaculina myanmarensis* and *N. gangetica* in Myanmar, with notes on two doubtful nominal taxa described as *Novaculina* members (Bivalvia: Pharidae). *Ecologica Montenegrina* **40**, 59–67; https://doi.org/10.37828/em.2021.40.4 (2021).

115. Thiele, J. *Handbook of Systematic Malacology. P. 3 (Scaphopoda/Bivalvia/Cephalopoda) and P. 4 (Comparative Morphology/Phylogeny/Geographical Distribution)* (Washington, D.C., 1998).
